# Supplementary material for: Population Substructure Has Implications in Validating Next-Generation Cancer Genomics Studies with TCGA
Source: Int J Mol Sci. 2019 Mar 8;20(5):1192. doi: 10.3390/ijms20051192 (PMC6429328; doi:10.3390/ijms20051192)
Supplement: Supplementary file 1 [file ijms-20-01192-s001.pdf]

Table 1. Q-matrix with 4 cluster solution at the subpopulations structure analysis of all TCGA patients. For each patient there is also information about tumor type and self-declared race and ethnicity.

FID	Tumor type	Ethnicity	Race	Cluster 1	Cluster 2	Cluster 3	Cluster 4
TCGA-2E-A9G8	Endometrial	Non-Hispanic	Black	0.000	0.046	0.954	0.000
TCGA-4E-A92E	Endometrial	Non-Hispanic	Black	0.143	0.000	0.256	0.601
TCGA-5B-A90C	Endometrial	Non-Hispanic	Black	0.000	0.000	1.000	0.000
TCGA-A5-A0G9	Endometrial	Unknown	White	0.014	0.000	0.727	0.258
TCGA-A5-A0GA	Endometrial	Non-Hispanic	White	0.000	0.000	0.338	0.662
TCGA-A5-A0GB	Endometrial	Unknown	White	0.000	0.000	0.344	0.656
TCGA-A5-A0GD	Endometrial	Unknown	White	0.000	0.000	0.372	0.628
TCGA-A5-A0GE	Endometrial	Non-Hispanic	Asian	0.000	0.000	0.530	0.470
TCGA-A5-A0GG	Endometrial	Non-Hispanic	Black	0.000	0.000	0.000	1.000
TCGA-A5-A0GH	Endometrial	Non-Hispanic	White	0.000	0.000	0.183	0.817
TCGA-A5-A0GI	Endometrial	Non-Hispanic	White	0.000	0.000	0.142	0.858
TCGA-A5-A0GJ	Endometrial	Non-Hispanic	White	0.000	0.000	0.000	1.000
TCGA-A5-A0GM	Endometrial	Non-Hispanic	White	0.000	0.000	0.731	0.269
TCGA-A5-A0GN	Endometrial	Non-Hispanic	White	0.000	0.000	0.484	0.516
TCGA-A5-A0GP	Endometrial	Non-Hispanic	Asian	0.000	0.000	1.000	0.000
TCGA-A5-A0GQ	Endometrial	Non-Hispanic	White	0.000	0.000	0.909	0.091
TCGA-A5-A0GR	Endometrial	Non-Hispanic	Black	0.000	0.000	0.189	0.811
TCGA-A5-A0GU	Endometrial	Unknown	White	0.037	0.000	0.510	0.453

TCGA-A5-A0GV	Endometrial	Non-Hispanic	White	0.000	0.000	0.000	1.000
TCGA-A5-A0GW	Endometrial	Non-Hispanic	Asian	0.000	0.000	0.000	1.000
TCGA-A5-A0GX	Endometrial	Unknown	White	0.000	0.000	0.326	0.674
TCGA-A5-A0R7	Endometrial	Non-Hispanic	White	0.000	0.000	0.000	1.000
TCGA-A5-A0R8	Endometrial	Non-Hispanic	White	0.000	0.000	0.250	0.749
TCGA-A5-A0R9	Endometrial	Non-Hispanic	White	0.042	0.000	0.268	0.689
TCGA-A5-A0RA	Endometrial	Non-Hispanic	White	0.000	0.000	1.000	0.000
TCGA-A5-A0VO	Endometrial	Non-Hispanic	White	0.000	0.000	0.488	0.512
TCGA-A5-A0VP	Endometrial	Non-Hispanic	White	0.000	0.000	0.000	1.000
TCGA-A5-A0VQ	Endometrial	Hispanic	Unknown	0.000	0.000	0.398	0.602
TCGA-A5-A1OJ	Endometrial	Non-Hispanic	Asian	0.000	0.000	0.633	0.367
TCGA-A5-A1OK	Endometrial	Non-Hispanic	White	0.000	0.000	0.414	0.586
TCGA-A5-A2K5	Endometrial	Non-Hispanic	White	0.011	0.000	0.000	0.989
TCGA-A5-A2K7	Endometrial	Hispanic	Unknown	0.011	0.000	0.359	0.630
TCGA-A5-A3LO	Endometrial	Non-Hispanic	White	0.162	0.000	0.000	0.838
TCGA-A5-A7WJ	Endometrial	Non-Hispanic	Black	0.006	0.002	0.403	0.589
TCGA-A5-AB3J	Endometrial	Non-Hispanic	Black	0.000	0.071	0.000	0.929
TCGA-AJ-A23O	Endometrial	Non-Hispanic	White	0.031	0.000	0.189	0.781
TCGA-AJ-A2QK	Endometrial	Hispanic	White	0.000	0.000	0.536	0.464
TCGA-AJ-A2QL	Endometrial	Non-Hispanic	White	0.000	0.000	0.694	0.306
TCGA-AJ-A2QN	Endometrial	Unknown	White	0.000	0.100	0.290	0.611

TCGA-AJ-A2QO	Endometrial	Unknown	White	0.000	0.000	0.000	1.000
TCGA-AJ-A3BH	Endometrial	Non-Hispanic	White	0.000	0.000	0.000	1.000
TCGA-AJ-A3BI	Endometrial	Non-Hispanic	White	0.000	0.000	0.000	1.000
TCGA-AJ-A3BK	Endometrial	Non-Hispanic	White	0.037	0.000	0.117	0.846
TCGA-AJ-A3EK	Endometrial	Hispanic	White	0.000	0.000	0.000	1.000
TCGA-AJ-A3EL	Endometrial	Unknown	Pacific	0.050	0.000	0.204	0.746
TCGA-AJ-A3EM	Endometrial	Unknown	White	0.000	0.154	0.000	0.846
TCGA-AJ-A3I9	Endometrial	Non-Hispanic	White	0.020	0.000	0.000	0.980
TCGA-AJ-A3NC	Endometrial	Unknown	White	0.217	0.000	0.000	0.783
TCGA-AJ-A3NE	Endometrial	Non-Hispanic	White	0.000	0.068	0.620	0.312
TCGA-AJ-A3OJ	Endometrial	Non-Hispanic	White	0.000	0.000	0.488	0.512
TCGA-AJ-A3OL	Endometrial	Non-Hispanic	White	0.171	0.000	0.000	0.829
TCGA-AJ-A5DV	Endometrial	Non-Hispanic	Black	0.066	0.000	0.000	0.934
TCGA-AJ-A8CT	Endometrial	Unknown	Black	0.019	0.000	0.332	0.649
TCGA-AJ-A8CV	Endometrial	Unknown	Black	0.014	0.000	0.000	0.986
TCGA-AJ-A8CW	Endometrial	Non-Hispanic	Black	0.069	0.000	0.000	0.931
TCGA-AP-A051	Endometrial	Unknown	White	0.020	0.000	0.334	0.646
TCGA-AP-A053	Endometrial	Non-Hispanic	White	0.000	0.000	0.335	0.665
TCGA-AP-A054	Endometrial	Non-Hispanic	White	0.024	0.000	0.415	0.561
TCGA-AP-A056	Endometrial	Unknown	White	0.000	0.000	0.000	1.000
TCGA-AP-A059	Endometrial	Non-Hispanic	White	0.008	0.000	0.342	0.649

TCGA-AP-A05N	Endometrial	Unknown	White	0.000	0.000	0.000	1.000
TCGA-AP-A05O	Endometrial	Unknown	White	0.017	0.000	0.519	0.465
TCGA-AP-A05P	Endometrial	Non-Hispanic	Asian	0.000	0.000	0.363	0.637
TCGA-AP-A0LD	Endometrial	Unknown	White	0.000	0.000	0.000	1.000
TCGA-AP-A0LE	Endometrial	Unknown	White	0.000	0.000	0.546	0.454
TCGA-AP-A0LF	Endometrial	Non-Hispanic	White	0.000	0.000	1.000	0.000
TCGA-AP-A0LG	Endometrial	Unknown	White	0.015	0.000	0.362	0.623
TCGA-AP-A0LJ	Endometrial	Unknown	White	0.000	0.000	0.681	0.319
TCGA-AP-A0LL	Endometrial	Unknown	White	0.000	0.000	0.000	1.000
TCGA-AP-A0LM	Endometrial	Unknown	Black	0.052	0.000	0.948	0.000
TCGA-AP-A0LN	Endometrial	Unknown	Black	0.014	0.000	0.000	0.986
TCGA-AP-A0LO	Endometrial	Non-Hispanic	White	0.000	0.000	0.000	1.000
TCGA-AP-A0LP	Endometrial	Unknown	White	0.000	0.000	0.526	0.474
TCGA-AP-A0LS	Endometrial	Unknown	White	0.000	0.017	0.298	0.685
TCGA-AP-A0LT	Endometrial	Unknown	White	0.000	0.000	0.000	1.000
TCGA-AP-A0LV	Endometrial	Unknown	White	0.000	0.000	0.230	0.769
TCGA-AP-A1DH	Endometrial	Unknown	White	0.000	0.000	0.000	1.000
TCGA-AP-A1DK	Endometrial	Unknown	White	0.000	0.039	0.000	0.961
TCGA-AP-A1DM	Endometrial	Unknown	White	0.000	0.000	0.443	0.557
TCGA-AP-A1DO	Endometrial	Non-Hispanic	White	0.000	0.000	0.000	1.000
TCGA-AP-A1DP	Endometrial	Unknown	White	0.045	0.000	0.497	0.457

TCGA-AP-A1DR	Endometrial	Unknown	Black	0.000	0.000	0.225	0.775
TCGA-AP-A1DV	Endometrial	Non-Hispanic	White	0.017	0.000	0.000	0.983
TCGA-AP-A1E0	Endometrial	Unknown	White	0.000	0.000	0.620	0.380
TCGA-AP-A1E1	Endometrial	Unknown	White	0.000	0.000	0.000	1.000
TCGA-AP-A1E3	Endometrial	Unknown	White	0.000	0.000	0.064	0.936
TCGA-AP-A1E4	Endometrial	Unknown	White	0.093	0.000	0.000	0.907
TCGA-AW-A1PO	Endometrial	Non-Hispanic	White	0.007	0.000	0.377	0.615
TCGA-AX-A05S	Endometrial	Non-Hispanic	White	0.000	0.000	0.601	0.399
TCGA-AX-A05T	Endometrial	Unknown	White	0.000	0.000	0.000	1.000
TCGA-AX-A05U	Endometrial	Non-Hispanic	American Indian	0.000	0.000	0.559	0.441
TCGA-AX-A05W	Endometrial	Non-Hispanic	White	0.000	0.000	0.475	0.525
TCGA-AX-A05Y	Endometrial	Non-Hispanic	White	0.000	0.000	1.000	0.000
TCGA-AX-A05Z	Endometrial	Hispanic	Unknown	0.000	0.000	0.000	1.000
TCGA-AX-A060	Endometrial	Non-Hispanic	American Indian	0.000	0.000	0.330	0.670
TCGA-AX-A062	Endometrial	Unknown	White	0.000	0.000	0.000	1.000
TCGA-AX-A063	Endometrial	Non-Hispanic	White	0.000	0.000	0.385	0.615
TCGA-AX-A064	Endometrial	Unknown	White	0.000	0.000	0.000	1.000
TCGA-AX-A06B	Endometrial	Non-Hispanic	White	0.000	0.000	0.000	1.000
TCGA-AX-A06D	Endometrial	Unknown	White	0.037	0.000	0.000	0.963
TCGA-AX-A06F	Endometrial	Non-Hispanic	White	0.000	0.000	0.000	1.000
TCGA-AX-A06H	Endometrial	Non-Hispanic	American Indian	0.000	0.000	0.260	0.740

TCGA-AX-A06J	Endometrial	Non-Hispanic	White	0.000	0.000	0.000	1.000
TCGA-AX-A06L	Endometrial	Unknown	White	0.000	0.059	0.941	0.000
TCGA-AX-A0IS	Endometrial	Non-Hispanic	White	0.000	0.000	1.000	0.000
TCGA-AX-A0IZ	Endometrial	Non-Hispanic	White	0.000	0.000	0.384	0.616
TCGA-AX-A0J0	Endometrial	Non-Hispanic	White	0.171	0.000	0.306	0.522
TCGA-AX-A0J1	Endometrial	Non-Hispanic	White	0.000	0.000	0.547	0.453
TCGA-AX-A1C4	Endometrial	Non-Hispanic	White	0.000	0.000	0.363	0.637
TCGA-AX-A1C5	Endometrial	Non-Hispanic	White	0.000	0.000	1.000	0.000
TCGA-AX-A1C9	Endometrial	Non-Hispanic	White	0.000	0.000	0.198	0.802
TCGA-AX-A1CE	Endometrial	Non-Hispanic	White	0.048	0.000	0.446	0.506
TCGA-AX-A1CF	Endometrial	Non-Hispanic	White	0.033	0.000	0.389	0.578
TCGA-AX-A1CI	Endometrial	Non-Hispanic	White	0.000	0.000	1.000	0.000
TCGA-AX-A1CJ	Endometrial	Non-Hispanic	Black	0.000	0.000	0.000	1.000
TCGA-AX-A1CK	Endometrial	Non-Hispanic	White	0.088	0.000	0.394	0.518
TCGA-AX-A1CN	Endometrial	Non-Hispanic	White	0.000	0.000	0.000	1.000
TCGA-AX-A2H7	Endometrial	Non-Hispanic	White	0.021	0.000	0.604	0.375
TCGA-AX-A2H8	Endometrial	Non-Hispanic	White	0.034	0.000	0.459	0.507
TCGA-AX-A2HA	Endometrial	Unknown	White	0.017	0.000	0.000	0.983
TCGA-AX-A2HC	Endometrial	Non-Hispanic	Black	0.000	0.000	0.000	1.000
TCGA-AX-A2HD	Endometrial	Non-Hispanic	White	0.086	0.000	0.343	0.570
TCGA-AX-A2HG	Endometrial	Hispanic	White	0.000	0.000	0.485	0.515

TCGA-AX-A2HH	Endometrial	Non-Hispanic	White	0.022	0.000	0.000	0.978
TCGA-AX-A2HJ	Endometrial	Non-Hispanic	Asian	0.000	0.000	0.177	0.823
TCGA-AX-A2HK	Endometrial	Unknown	White	0.000	0.000	0.633	0.367
TCGA-AX-A2IN	Endometrial	Unknown	White	0.000	0.000	0.000	1.000
TCGA-AX-A3FS	Endometrial	Non-Hispanic	White	0.012	0.000	0.000	0.988
TCGA-AX-A3FT	Endometrial	Non-Hispanic	White	0.000	0.000	0.000	1.000
TCGA-AX-A3FV	Endometrial	Non-Hispanic	White	0.000	0.000	1.000	0.000
TCGA-AX-A3FW	Endometrial	Non-Hispanic	White	0.071	0.000	0.929	0.000
TCGA-AX-A3FX	Endometrial	Non-Hispanic	White	0.016	0.000	0.238	0.747
TCGA-AX-A3FZ	Endometrial	Non-Hispanic	White	0.185	0.000	0.000	0.815
TCGA-AX-A3G1	Endometrial	Non-Hispanic	White	0.000	0.000	0.394	0.606
TCGA-AX-A3G8	Endometrial	Unknown	White	0.000	0.000	0.395	0.605
TCGA-AX-A3G9	Endometrial	Non-Hispanic	White	0.000	0.000	0.544	0.456
TCGA-AX-A3GB	Endometrial	Non-Hispanic	White	0.015	0.000	0.426	0.559
TCGA-B5-A0JR	Endometrial	Unknown	White	0.167	0.000	0.103	0.731
TCGA-B5-A0JS	Endometrial	Unknown	White	0.000	0.000	0.241	0.759
TCGA-B5-A0JT	Endometrial	Unknown	White	0.011	0.000	0.303	0.686
TCGA-B5-A0JV	Endometrial	Unknown	Black	0.000	0.000	1.000	0.000
TCGA-B5-A0JX	Endometrial	Unknown	White	0.000	0.000	1.000	0.000
TCGA-B5-A0JY	Endometrial	Unknown	White	0.000	0.000	0.000	1.000
TCGA-B5-A0JZ	Endometrial	Unknown	White	0.000	0.000	0.450	0.550

TCGA-B5-A0K0	Endometrial	Unknown	White	0.000	0.000	0.494	0.506
TCGA-B5-A0K1	Endometrial	Unknown	White	0.016	0.000	0.000	0.984
TCGA-B5-A0K2	Endometrial	Unknown	White	0.000	0.000	0.544	0.456
TCGA-B5-A0K3	Endometrial	Unknown	Black	0.000	0.051	0.000	0.949
TCGA-B5-A0K4	Endometrial	Unknown	Unknown	0.000	0.000	0.402	0.598
TCGA-B5-A0K6	Endometrial	Unknown	White	0.000	0.000	0.000	1.000
TCGA-B5-A0K7	Endometrial	Unknown	White	0.008	0.029	0.748	0.216
TCGA-B5-A0K9	Endometrial	Unknown	White	0.000	0.000	0.000	1.000
TCGA-B5-A11E	Endometrial	Unknown	White	0.000	0.000	1.000	0.000
TCGA-B5-A11F	Endometrial	Unknown	White	0.000	0.000	1.000	0.000
TCGA-B5-A11G	Endometrial	Unknown	Black	0.000	0.000	0.534	0.466
TCGA-B5-A11H	Endometrial	Unknown	White	0.000	0.026	0.000	0.974
TCGA-B5-A11I	Endometrial	Unknown	Black	0.000	0.146	0.580	0.274
TCGA-B5-A11J	Endometrial	Unknown	White	0.000	0.000	1.000	0.000
TCGA-B5-A11M	Endometrial	Unknown	White	0.124	0.000	0.209	0.667
TCGA-B5-A11N	Endometrial	Unknown	White	0.000	0.000	1.000	0.000
TCGA-B5-A11O	Endometrial	Unknown	White	0.000	0.000	0.146	0.854
TCGA-B5-A11Q	Endometrial	Unknown	White	0.000	0.000	0.000	1.000
TCGA-B5-A11R	Endometrial	Unknown	Black	0.000	0.001	0.000	0.999
TCGA-B5-A11S	Endometrial	Unknown	White	0.000	0.000	0.442	0.558
TCGA-B5-A11U	Endometrial	Unknown	Black	0.016	0.000	0.559	0.425

TCGA-B5-A11V	Endometrial	Unknown	Black	0.000	0.000	0.677	0.323
TCGA-B5-A11W	Endometrial	Unknown	White	0.000	0.022	0.000	0.978
TCGA-B5-A11X	Endometrial	Unknown	Black	0.000	0.000	1.000	0.000
TCGA-B5-A11Y	Endometrial	Unknown	White	0.017	0.000	0.983	0.000
TCGA-B5-A11Z	Endometrial	Unknown	White	0.063	0.000	0.000	0.937
TCGA-B5-A121	Endometrial	Unknown	White	0.033	0.000	0.000	0.967
TCGA-B5-A1MR	Endometrial	Unknown	White	0.000	0.034	0.507	0.459
TCGA-B5-A1MV	Endometrial	Unknown	White	0.000	0.000	0.000	1.000
TCGA-B5-A1MW	Endometrial	Unknown	White	0.188	0.000	0.473	0.340
TCGA-B5-A1MX	Endometrial	Unknown	White	0.000	0.000	0.000	1.000
TCGA-B5-A1MZ	Endometrial	Unknown	White	0.000	0.000	0.542	0.458
TCGA-B5-A3F9	Endometrial	Unknown	Black	0.067	0.000	0.197	0.736
TCGA-B5-A3FA	Endometrial	Unknown	White	0.000	0.000	0.000	1.000
TCGA-B5-A3FB	Endometrial	Unknown	Black	0.008	0.000	0.544	0.448
TCGA-B5-A3FC	Endometrial	Non-Hispanic	Black	0.022	0.000	0.252	0.727
TCGA-B5-A3FD	Endometrial	Unknown	Black	0.000	0.000	1.000	0.000
TCGA-B5-A3FH	Endometrial	Unknown	Black	0.000	0.000	0.427	0.573
TCGA-B5-A5OC	Endometrial	Unknown	Black	0.038	0.035	0.000	0.927
TCGA-BG-A0LW	Endometrial	Non-Hispanic	White	0.000	0.000	0.308	0.692
TCGA-BG-A0LX	Endometrial	Non-Hispanic	White	0.009	0.000	0.000	0.991
TCGA-BG-A0M0	Endometrial	Non-Hispanic	White	0.051	0.000	0.547	0.401

TCGA-BG-A0M2	Endometrial	Non-Hispanic	White	0.000	0.000	0.000	1.000
TCGA-BG-A0M3	Endometrial	Non-Hispanic	White	0.000	0.000	0.000	1.000
TCGA-BG-A0M4	Endometrial	Non-Hispanic	White	0.000	0.000	0.000	1.000
TCGA-BG-A0M7	Endometrial	Non-Hispanic	White	0.000	0.000	0.240	0.760
TCGA-BG-A0M8	Endometrial	Non-Hispanic	White	0.000	0.000	0.000	1.000
TCGA-BG-A0M9	Endometrial	Non-Hispanic	White	0.000	0.000	0.455	0.545
TCGA-BG-A0MA	Endometrial	Non-Hispanic	White	0.000	0.000	0.509	0.491
TCGA-BG-A0MC	Endometrial	Non-Hispanic	White	0.000	0.000	0.000	1.000
TCGA-BG-A0MG	Endometrial	Non-Hispanic	White	0.000	0.000	0.000	1.000
TCGA-BG-A0MH	Endometrial	Unknown	White	0.000	0.000	0.259	0.741
TCGA-BG-A0MI	Endometrial	Non-Hispanic	White	0.000	0.000	0.235	0.765
TCGA-BG-A0MK	Endometrial	Non-Hispanic	Black	0.000	0.000	1.000	0.000
TCGA-BG-A0MO	Endometrial	Non-Hispanic	White	0.000	0.000	0.510	0.490
TCGA-BG-A0MQ	Endometrial	Non-Hispanic	White	0.000	0.000	1.000	0.000
TCGA-BG-A0MS	Endometrial	Non-Hispanic	White	0.000	0.000	0.000	1.000
TCGA-BG-A0MT	Endometrial	Non-Hispanic	White	0.021	0.000	0.979	0.000
TCGA-BG-A0MU	Endometrial	Non-Hispanic	White	0.030	0.000	0.460	0.510
TCGA-BG-A0RY	Endometrial	Non-Hispanic	White	0.000	0.000	0.253	0.747
TCGA-BG-A0VT	Endometrial	Non-Hispanic	White	0.000	0.000	0.724	0.276
TCGA-BG-A0VV	Endometrial	Non-Hispanic	White	0.000	0.000	0.305	0.695
TCGA-BG-A0VW	Endometrial	Non-Hispanic	White	0.000	0.000	0.739	0.261

TCGA-BG-A0VX	Endometrial	Non-Hispanic	White	0.000	0.000	0.813	0.187
TCGA-BG-A0VZ	Endometrial	Non-Hispanic	White	0.013	0.000	0.630	0.357
TCGA-BG-A0W1	Endometrial	Non-Hispanic	White	0.000	0.000	1.000	0.000
TCGA-BG-A0W2	Endometrial	Unknown	White	0.010	0.000	0.296	0.694
TCGA-BG-A0YU	Endometrial	Non-Hispanic	White	0.000	0.000	1.000	0.000
TCGA-BG-A186	Endometrial	Non-Hispanic	Black	0.000	0.000	0.535	0.465
TCGA-BG-A187	Endometrial	Non-Hispanic	White	0.001	0.026	0.166	0.807
TCGA-BG-A18A	Endometrial	Non-Hispanic	White	0.000	0.000	0.086	0.914
TCGA-BG-A18B	Endometrial	Non-Hispanic	White	0.000	0.000	0.000	1.000
TCGA-BG-A18C	Endometrial	Non-Hispanic	White	0.000	0.096	0.904	0.000
TCGA-BG-A220	Endometrial	Non-Hispanic	White	0.000	0.000	0.585	0.415
TCGA-BG-A221	Endometrial	Non-Hispanic	White	0.000	0.001	0.697	0.302
TCGA-BG-A222	Endometrial	Non-Hispanic	White	0.037	0.000	0.000	0.963
TCGA-BG-A2AD	Endometrial	Non-Hispanic	White	0.000	0.000	1.000	0.000
TCGA-BG-A2AE	Endometrial	Non-Hispanic	White	0.000	0.000	1.000	0.000
TCGA-BG-A2L7	Endometrial	Non-Hispanic	White	0.000	0.000	0.343	0.657
TCGA-BG-A3EW	Endometrial	Non-Hispanic	White	0.264	0.000	0.000	0.735
TCGA-BK-A0C9	Endometrial	Non-Hispanic	White	0.000	0.000	0.324	0.676
TCGA-BK-A0CB	Endometrial	Non-Hispanic	White	0.000	0.000	0.377	0.623
TCGA-BK-A139	Endometrial	Non-Hispanic	White	0.000	0.000	0.304	0.696
TCGA-BK-A13B	Endometrial	Non-Hispanic	Black	0.067	0.000	0.040	0.893

TCGA-BK-A13C	Endometrial	Non-Hispanic	Black	0.000	0.000	0.362	0.638
TCGA-BK-A4ZD	Endometrial	Non-Hispanic	Black	0.074	0.020	0.668	0.238
TCGA-BK-A56F	Endometrial	Non-Hispanic	Black	0.332	0.000	0.000	0.668
TCGA-BK-A6W3	Endometrial	Non-Hispanic	Black	0.094	0.000	0.385	0.522
TCGA-BK-A6W4	Endometrial	Non-Hispanic	Black	0.000	0.117	0.000	0.883
TCGA-BS-A0T9	Endometrial	Non-Hispanic	Pacific	0.000	0.000	1.000	0.000
TCGA-BS-A0TA	Endometrial	Hispanic	Pacific	0.000	0.000	0.159	0.841
TCGA-BS-A0TC	Endometrial	Non-Hispanic	Asian	0.000	0.000	0.626	0.374
TCGA-BS-A0TD	Endometrial	Non-Hispanic	Asian	0.000	0.000	0.000	1.000
TCGA-BS-A0TE	Endometrial	Non-Hispanic	Asian	0.000	0.000	0.344	0.656
TCGA-BS-A0TG	Endometrial	Non-Hispanic	White	0.000	0.000	0.389	0.611
TCGA-BS-A0TI	Endometrial	Non-Hispanic	White	0.000	0.005	0.000	0.995
TCGA-BS-A0TJ	Endometrial	Non-Hispanic	Asian	0.000	0.000	0.000	1.000
TCGA-BS-A0U5	Endometrial	Non-Hispanic	White	0.067	0.000	0.933	0.000
TCGA-BS-A0U7	Endometrial	Non-Hispanic	White	0.000	0.000	0.351	0.649
TCGA-BS-A0U8	Endometrial	Non-Hispanic	White	0.009	0.000	0.413	0.578
TCGA-BS-A0UA	Endometrial	Non-Hispanic	White	0.000	0.000	0.675	0.325
TCGA-BS-A0UF	Endometrial	Non-Hispanic	Asian	0.000	0.000	0.000	1.000
TCGA-BS-A0UJ	Endometrial	Non-Hispanic	Asian	0.017	0.000	0.000	0.983
TCGA-BS-A0UL	Endometrial	Non-Hispanic	Pacific	0.000	0.000	0.000	1.000
TCGA-BS-A0UM	Endometrial	Non-Hispanic	White	0.000	0.000	0.266	0.734

TCGA-BS-A0UT	Endometrial	Non-Hispanic	Asian	0.000	0.000	0.490	0.510
TCGA-BS-A0UV	Endometrial	Non-Hispanic	Pacific	0.000	0.000	0.200	0.800
TCGA-BS-A0V4	Endometrial	Non-Hispanic	White	0.000	0.016	0.000	0.984
TCGA-BS-A0V6	Endometrial	Non-Hispanic	Asian	0.000	0.000	0.699	0.301
TCGA-BS-A0V7	Endometrial	Non-Hispanic	Pacific	0.000	0.000	0.285	0.715
TCGA-BS-A0V8	Endometrial	Non-Hispanic	Pacific	0.000	0.085	0.402	0.513
TCGA-BS-A0VI	Endometrial	Non-Hispanic	White	0.000	0.000	0.602	0.398
TCGA-BS-A0WQ	Endometrial	Non-Hispanic	Asian	0.070	0.000	0.238	0.692
TCGA-D1-A0ZN	Endometrial	Non-Hispanic	White	0.036	0.000	0.000	0.964
TCGA-D1-A0ZO	Endometrial	Non-Hispanic	White	0.000	0.055	0.000	0.945
TCGA-D1-A0ZQ	Endometrial	Non-Hispanic	White	0.038	0.000	0.000	0.962
TCGA-D1-A0ZR	Endometrial	Non-Hispanic	White	0.040	0.000	0.366	0.594
TCGA-D1-A0ZS	Endometrial	Non-Hispanic	White	0.027	0.000	0.256	0.717
TCGA-D1-A0ZU	Endometrial	Non-Hispanic	White	0.000	0.000	0.000	1.000
TCGA-D1-A0ZV	Endometrial	Non-Hispanic	White	0.000	0.019	0.465	0.516
TCGA-D1-A101	Endometrial	Non-Hispanic	White	0.000	0.000	0.759	0.241
TCGA-D1-A102	Endometrial	Non-Hispanic	White	0.000	0.038	0.000	0.962
TCGA-D1-A103	Endometrial	Non-Hispanic	White	0.103	0.000	0.401	0.496
TCGA-D1-A15W	Endometrial	Non-Hispanic	White	0.057	0.000	0.366	0.577
TCGA-D1-A15Z	Endometrial	Non-Hispanic	White	0.000	0.000	1.000	0.000
TCGA-D1-A160	Endometrial	Non-Hispanic	White	0.000	0.000	1.000	0.000

TCGA-D1-A161	Endometrial	Non-Hispanic	White	0.021	0.000	0.199	0.780
TCGA-D1-A162	Endometrial	Non-Hispanic	White	0.006	0.000	0.236	0.758
TCGA-D1-A163	Endometrial	Non-Hispanic	White	0.000	0.000	0.452	0.548
TCGA-D1-A165	Endometrial	Non-Hispanic	White	0.078	0.000	0.363	0.558
TCGA-D1-A167	Endometrial	Non-Hispanic	White	0.101	0.000	0.712	0.187
TCGA-D1-A168	Endometrial	Non-Hispanic	White	0.000	0.000	0.098	0.902
TCGA-D1-A169	Endometrial	Non-Hispanic	White	0.000	0.008	0.000	0.992
TCGA-D1-A16B	Endometrial	Non-Hispanic	White	0.000	0.000	0.305	0.695
TCGA-D1-A16D	Endometrial	Non-Hispanic	White	0.000	0.000	0.679	0.321
TCGA-D1-A16E	Endometrial	Non-Hispanic	White	0.000	0.000	0.399	0.601
TCGA-D1-A16F	Endometrial	Non-Hispanic	White	0.000	0.000	0.000	1.000
TCGA-D1-A16J	Endometrial	Non-Hispanic	White	0.000	0.000	0.245	0.755
TCGA-D1-A16N	Endometrial	Non-Hispanic	White	0.000	0.000	0.000	1.000
TCGA-D1-A16O	Endometrial	Non-Hispanic	White	0.000	0.000	0.000	1.000
TCGA-D1-A16Q	Endometrial	Non-Hispanic	White	0.000	0.000	0.196	0.804
TCGA-D1-A16R	Endometrial	Non-Hispanic	White	0.000	0.000	0.198	0.802
TCGA-D1-A16V	Endometrial	Non-Hispanic	White	0.000	0.000	0.000	1.000
TCGA-D1-A16X	Endometrial	Non-Hispanic	White	0.000	0.000	0.436	0.564
TCGA-D1-A16Y	Endometrial	Non-Hispanic	White	0.000	0.000	0.302	0.698
TCGA-D1-A174	Endometrial	Non-Hispanic	White	0.000	0.000	0.345	0.655
TCGA-D1-A175	Endometrial	Non-Hispanic	White	0.068	0.058	0.556	0.318

TCGA-D1-A176	Endometrial	Non-Hispanic	White	0.000	0.000	0.304	0.696
TCGA-D1-A177	Endometrial	Non-Hispanic	White	0.000	0.000	0.556	0.444
TCGA-D1-A17A	Endometrial	Non-Hispanic	White	0.000	0.014	0.000	0.986
TCGA-D1-A17B	Endometrial	Non-Hispanic	White	0.000	0.000	0.427	0.573
TCGA-D1-A17C	Endometrial	Non-Hispanic	White	0.022	0.000	0.000	0.978
TCGA-D1-A17D	Endometrial	Non-Hispanic	White	0.000	0.000	1.000	0.000
TCGA-D1-A17F	Endometrial	Non-Hispanic	White	0.000	0.000	0.150	0.850
TCGA-D1-A17H	Endometrial	Non-Hispanic	White	0.000	0.000	0.280	0.720
TCGA-D1-A17K	Endometrial	Non-Hispanic	White	0.000	0.031	0.081	0.888
TCGA-D1-A17L	Endometrial	Non-Hispanic	White	0.000	0.000	0.398	0.602
TCGA-D1-A17M	Endometrial	Non-Hispanic	White	0.000	0.000	0.665	0.335
TCGA-D1-A17N	Endometrial	Non-Hispanic	Unknown	0.000	0.000	0.000	1.000
TCGA-D1-A17Q	Endometrial	Non-Hispanic	White	0.000	0.000	0.331	0.669
TCGA-D1-A17R	Endometrial	Non-Hispanic	White	0.012	0.000	0.540	0.448
TCGA-D1-A17S	Endometrial	Non-Hispanic	White	0.000	0.000	0.680	0.320
TCGA-D1-A17T	Endometrial	Non-Hispanic	White	0.000	0.000	0.634	0.366
TCGA-D1-A17U	Endometrial	Non-Hispanic	White	0.000	0.000	0.290	0.710
TCGA-D1-A1NS	Endometrial	Non-Hispanic	White	0.000	0.000	0.297	0.703
TCGA-D1-A1NY	Endometrial	Non-Hispanic	White	0.000	0.000	0.000	1.000
TCGA-D1-A1NZ	Endometrial	Non-Hispanic	White	0.000	0.000	0.448	0.552
TCGA-D1-A1O0	Endometrial	Non-Hispanic	White	0.020	0.000	0.417	0.563

TCGA-D1-A1O5	Endometrial	Non-Hispanic	White	0.000	0.011	0.271	0.718
TCGA-D1-A1O7	Endometrial	Non-Hispanic	White	0.017	0.000	0.983	0.000
TCGA-D1-A1O8	Endometrial	Non-Hispanic	White	0.004	0.000	0.628	0.368
TCGA-D1-A2G5	Endometrial	Non-Hispanic	White	0.000	0.000	0.000	1.000
TCGA-D1-A2G6	Endometrial	Non-Hispanic	White	0.000	0.000	0.277	0.723
TCGA-D1-A3DA	Endometrial	Non-Hispanic	White	0.143	0.000	0.000	0.857
TCGA-D1-A3DG	Endometrial	Non-Hispanic	White	0.006	0.000	0.730	0.263
TCGA-D1-A3DH	Endometrial	Non-Hispanic	White	0.055	0.000	0.000	0.945
TCGA-DF-A2KN	Endometrial	Unknown	Unknown	0.057	0.000	0.360	0.583
TCGA-DF-A2KS	Endometrial	Unknown	Unknown	0.000	0.000	0.000	1.000
TCGA-DF-A2KU	Endometrial	Unknown	Unknown	0.000	0.000	0.000	1.000
TCGA-DF-A2KV	Endometrial	Unknown	Unknown	0.076	0.000	0.184	0.740
TCGA-DF-A2KY	Endometrial	Unknown	Unknown	0.000	0.019	0.000	0.981
TCGA-DF-A2KZ	Endometrial	Unknown	Unknown	0.000	0.000	0.074	0.926
TCGA-DF-A2L0	Endometrial	Unknown	Unknown	0.000	0.000	0.000	1.000
TCGA-DI-A0WH	Endometrial	Non-Hispanic	White	0.072	0.000	0.379	0.548
TCGA-DI-A1BY	Endometrial	Non-Hispanic	White	0.000	0.000	0.413	0.587
TCGA-DI-A1NO	Endometrial	Non-Hispanic	Black	0.000	0.098	0.222	0.679
TCGA-DI-A2QU	Endometrial	Non-Hispanic	White	0.000	0.042	0.697	0.261
TCGA-E6-A1LX	Endometrial	Non-Hispanic	White	0.000	0.000	0.995	0.005
TCGA-E6-A1M0	Endometrial	Non-Hispanic	White	0.000	0.000	0.133	0.867

TCGA-E6-A2P9	Endometrial	Non-Hispanic	White	0.012	0.000	0.000	0.988
TCGA-EC-A1NJ	Endometrial	Non-Hispanic	White	0.000	0.000	0.000	1.000
TCGA-EC-A1QX	Endometrial	Non-Hispanic	White	0.000	0.000	0.620	0.380
TCGA-EC-A24G	Endometrial	Non-Hispanic	White	0.000	0.000	0.000	1.000
TCGA-EO-A1Y7	Endometrial	Unknown	Unknown	0.000	0.015	0.360	0.625
TCGA-EO-A22R	Endometrial	Unknown	Unknown	0.000	0.000	0.479	0.521
TCGA-EO-A22S	Endometrial	Unknown	Unknown	0.000	0.000	0.204	0.796
TCGA-EO-A22T	Endometrial	Non-Hispanic	Asian	0.000	0.000	0.000	1.000
TCGA-EO-A22U	Endometrial	Unknown	White	0.002	0.076	0.257	0.665
TCGA-EO-A22X	Endometrial	Unknown	Unknown	0.062	0.000	0.203	0.735
TCGA-EO-A22Y	Endometrial	Non-Hispanic	White	0.244	0.000	0.000	0.756
TCGA-EO-A3AS	Endometrial	Unknown	Unknown	0.140	0.000	0.000	0.860
TCGA-EO-A3AU	Endometrial	Unknown	Unknown	0.000	0.000	0.289	0.711
TCGA-EO-A3AY	Endometrial	Unknown	Unknown	0.000	0.000	0.097	0.902
TCGA-EO-A3B0	Endometrial	Unknown	Asian	0.000	0.044	0.000	0.956
TCGA-EO-A3KX	Endometrial	Hispanic	White	0.143	0.000	0.036	0.820
TCGA-EO-A3L0	Endometrial	Non-Hispanic	White	0.127	0.000	0.000	0.873
TCGA-EY-A1G7	Endometrial	Non-Hispanic	White	0.000	0.000	0.335	0.665
TCGA-EY-A1G8	Endometrial	Non-Hispanic	White	0.000	0.000	0.420	0.579
TCGA-EY-A1GC	Endometrial	Non-Hispanic	White	0.000	0.000	0.625	0.375
TCGA-EY-A1GD	Endometrial	Non-Hispanic	Black	0.000	0.000	1.000	0.000

TCGA-EY-A1GE	Endometrial	Non-Hispanic	White	0.063	0.000	0.937	0.000
TCGA-EY-A1GF	Endometrial	Non-Hispanic	Black	0.048	0.000	0.778	0.174
TCGA-EY-A1GH	Endometrial	Non-Hispanic	White	0.000	0.044	0.520	0.437
TCGA-EY-A1GI	Endometrial	Non-Hispanic	White	0.017	0.000	0.552	0.431
TCGA-EY-A1GK	Endometrial	Non-Hispanic	White	0.000	0.000	0.856	0.144
TCGA-EY-A1GL	Endometrial	Unknown	White	0.055	0.022	0.000	0.922
TCGA-EY-A1GP	Endometrial	Non-Hispanic	Black	0.209	0.000	0.000	0.791
TCGA-EY-A1GQ	Endometrial	Non-Hispanic	White	0.000	0.000	0.584	0.416
TCGA-EY-A1GR	Endometrial	Non-Hispanic	Black	0.000	0.000	0.583	0.417
TCGA-EY-A1GT	Endometrial	Non-Hispanic	White	0.000	0.000	1.000	0.000
TCGA-EY-A1GU	Endometrial	Non-Hispanic	Black	0.000	0.000	0.000	1.000
TCGA-EY-A1GW	Endometrial	Hispanic	White	0.000	0.000	0.119	0.881
TCGA-EY-A1GX	Endometrial	Non-Hispanic	White	0.176	0.000	0.000	0.824
TCGA-EY-A1H0	Endometrial	Non-Hispanic	Black	0.000	0.000	0.337	0.663
TCGA-EY-A214	Endometrial	Unknown	White	0.000	0.000	0.000	1.000
TCGA-EY-A215	Endometrial	Non-Hispanic	White	0.062	0.000	0.382	0.556
TCGA-EY-A2OM	Endometrial	Non-Hispanic	White	0.000	0.000	0.384	0.616
TCGA-EY-A2OP	Endometrial	Non-Hispanic	White	0.147	0.000	0.330	0.522
TCGA-EY-A2OQ	Endometrial	Non-Hispanic	White	0.090	0.000	0.444	0.466
TCGA-EY-A548	Endometrial	Non-Hispanic	Black	0.219	0.000	0.000	0.781
TCGA-EY-A549	Endometrial	Non-Hispanic	Black	0.138	0.000	0.319	0.543

TCGA-EY-A54A	Endometrial	Non-Hispanic	Black	0.068	0.014	0.000	0.918
TCGA-EY-A5W2	Endometrial	Non-Hispanic	Black	0.000	0.000	0.409	0.591
TCGA-EY-A72D	Endometrial	Non-Hispanic	Black	0.000	0.000	0.099	0.901
TCGA-FI-A2CX	Endometrial	Non-Hispanic	White	0.000	0.000	1.000	0.000
TCGA-FI-A2D0	Endometrial	Non-Hispanic	White	0.052	0.000	0.517	0.431
TCGA-FI-A2D4	Endometrial	Unknown	White	0.000	0.000	0.771	0.229
TCGA-FI-A2D5	Endometrial	Non-Hispanic	White	0.000	0.000	0.000	1.000
TCGA-FI-A2D6	Endometrial	Non-Hispanic	White	0.000	0.000	0.241	0.759
TCGA-FI-A2F4	Endometrial	Non-Hispanic	Black	0.000	0.000	1.000	0.000
TCGA-FI-A2F9	Endometrial	Non-Hispanic	White	0.000	0.089	0.654	0.257
TCGA-H5-A2HR	Endometrial	Non-Hispanic	Black	0.000	0.000	0.723	0.277
TCGA-PG-A916	Endometrial	Non-Hispanic	Black	0.000	0.000	0.193	0.807
TCGA-PG-A917	Endometrial	Non-Hispanic	Black	0.000	0.000	0.333	0.667
TCGA-QF-A5YT	Endometrial	Non-Hispanic	Black	0.000	0.000	0.000	1.000
TCGA-QS-A5YQ	Endometrial	Non-Hispanic	Black	0.000	0.000	1.000	0.000
TCGA-QS-A744	Endometrial	Non-Hispanic	Black	0.146	0.077	0.512	0.264
TCGA-SJ-A6ZI	Endometrial	Non-Hispanic	Black	0.072	0.000	0.358	0.570
TCGA-SJ-A6ZJ	Endometrial	Non-Hispanic	Black	0.041	0.000	0.000	0.959
TCGA-SL-A6J9	Endometrial	Non-Hispanic	Black	0.000	0.086	0.201	0.714
TCGA-SL-A6JA	Endometrial	Non-Hispanic	Black	0.075	0.000	0.321	0.604
TCGA-04-1331	Ovary	Non-Hispanic	White	0.000	0.703	0.072	0.224

TCGA-04-1332	Ovary	Non-Hispanic	White	0.000	0.493	0.238	0.269
TCGA-04-1338	Ovary	Non-Hispanic	White	0.000	0.134	0.193	0.673
TCGA-04-1341	Ovary	Non-Hispanic	White	0.000	0.316	0.412	0.272
TCGA-04-1343	Ovary	Non-Hispanic	White	0.032	0.699	0.000	0.269
TCGA-04-1347	Ovary	Non-Hispanic	White	0.008	0.350	0.000	0.642
TCGA-04-1350	Ovary	Unknown	White	0.078	0.102	0.450	0.370
TCGA-04-1356	Ovary	Hispanic	White	0.365	0.184	0.000	0.450
TCGA-04-1357	Ovary	Unknown	Unknown	0.048	0.247	0.705	0.000
TCGA-04-1361	Ovary	Unknown	White	0.079	0.691	0.000	0.230
TCGA-04-1362	Ovary	Unknown	White	0.655	0.000	0.000	0.345
TCGA-04-1364	Ovary	Non-Hispanic	White	0.000	0.360	0.309	0.331
TCGA-04-1365	Ovary	Non-Hispanic	White	0.116	0.459	0.253	0.172
TCGA-04-1514	Ovary	Non-Hispanic	White	0.847	0.000	0.153	0.000
TCGA-04-1519	Ovary	Unknown	Unknown	0.390	0.097	0.513	0.000
TCGA-04-1530	Ovary	Non-Hispanic	White	0.313	0.266	0.000	0.420
TCGA-04-1536	Ovary	Unknown	Black	0.120	0.092	0.441	0.346
TCGA-04-1648	Ovary	Unknown	White	0.603	0.000	0.000	0.397
TCGA-04-1651	Ovary	Non-Hispanic	White	0.484	0.000	0.516	0.000
TCGA-04-1655	Ovary	Unknown	White	0.288	0.000	0.168	0.544
TCGA-09-0364	Ovary	Non-Hispanic	White	0.506	0.000	0.000	0.494
TCGA-09-0366	Ovary	Unknown	White	0.355	0.619	0.000	0.025
TCGA-09-0367	Ovary	Non-Hispanic	Pacific	0.000	0.056	0.613	0.331
TCGA-09-0369	Ovary	Non-Hispanic	White	0.006	0.167	0.301	0.526
TCGA-09-1659	Ovary	Non-Hispanic	White	0.000	0.518	0.000	0.482
TCGA-09-1661	Ovary	Non-Hispanic	White	0.042	0.089	0.416	0.453
TCGA-09-1662	Ovary	Non-Hispanic	White	0.719	0.000	0.281	0.000

TCGA-09-1665	Ovary	Non-Hispanic	White	0.000	1.000	0.000	0.000
TCGA-09-1666	Ovary	Non-Hispanic	White	0.415	0.000	0.035	0.550
TCGA-09-1667	Ovary	Non-Hispanic	White	0.092	0.000	0.908	0.000
TCGA-09-1668	Ovary	Non-Hispanic	White	0.139	0.310	0.000	0.550
TCGA-09-1669	Ovary	Non-Hispanic	White	0.350	0.301	0.349	0.000
TCGA-09-1670	Ovary	Unknown	White	0.131	0.170	0.532	0.168
TCGA-09-1673	Ovary	Non-Hispanic	White	0.284	0.000	0.049	0.667
TCGA-09-2045	Ovary	Non-Hispanic	Asian	0.284	0.000	0.394	0.322
TCGA-09-2048	Ovary	Non-Hispanic	White	0.000	0.786	0.214	0.000
TCGA-09-2053	Ovary	Non-Hispanic	White	0.195	0.192	0.612	0.000
TCGA-09-2054	Ovary	Non-Hispanic	Black	0.000	0.276	0.557	0.166
TCGA-09-2056	Ovary	Hispanic	White	0.136	0.137	0.399	0.328
TCGA-10-0927	Ovary	Hispanic	Unknown	0.000	0.038	0.000	0.962
TCGA-10-0928	Ovary	Unknown	White	0.229	0.000	0.771	0.000
TCGA-10-0931	Ovary	Unknown	White	0.254	0.165	0.581	0.000
TCGA-10-0933	Ovary	Non-Hispanic	White	0.000	0.426	0.136	0.438
TCGA-10-0936	Ovary	Non-Hispanic	Black	0.000	0.590	0.410	0.000
TCGA-10-0937	Ovary	Unknown	White	0.069	0.000	0.568	0.363
TCGA-10-0938	Ovary	Unknown	White	0.346	0.128	0.214	0.312
TCGA-13-0714	Ovary	Non-Hispanic	White	0.000	0.105	0.444	0.451
TCGA-13-0720	Ovary	Non-Hispanic	White	0.161	0.124	0.456	0.258
TCGA-13-0724	Ovary	Hispanic	White	0.000	0.440	0.246	0.314
TCGA-13-0725	Ovary	Hispanic	White	0.154	0.101	0.709	0.037
TCGA-13-0726	Ovary	Non-Hispanic	White	0.105	0.295	0.000	0.599
TCGA-13-0727	Ovary	Non-Hispanic	White	0.184	0.137	0.442	0.237
TCGA-13-0730	Ovary	Non-Hispanic	White	0.000	0.105	0.895	0.000

TCGA-13-0762	Ovary	Non-Hispanic	White	0.388	0.282	0.330	0.000
TCGA-13-0765	Ovary	Non-Hispanic	White	0.109	0.425	0.000	0.466
TCGA-13-0766	Ovary	Non-Hispanic	White	0.000	0.116	0.441	0.443
TCGA-13-0768	Ovary	Non-Hispanic	White	0.315	0.218	0.466	0.000
TCGA-13-0795	Ovary	Non-Hispanic	White	0.000	0.312	0.000	0.688
TCGA-13-0797	Ovary	Non-Hispanic	White	0.000	0.080	0.566	0.354
TCGA-13-0800	Ovary	Non-Hispanic	White	0.000	0.516	0.484	0.000
TCGA-13-0804	Ovary	Non-Hispanic	White	0.000	0.446	0.177	0.377
TCGA-13-0883	Ovary	Non-Hispanic	White	0.409	0.000	0.591	0.000
TCGA-13-0884	Ovary	Non-Hispanic	White	0.536	0.000	0.000	0.464
TCGA-13-0885	Ovary	Non-Hispanic	White	0.035	0.200	0.573	0.192
TCGA-13-0886	Ovary	Non-Hispanic	White	0.282	0.175	0.543	0.000
TCGA-13-0887	Ovary	Non-Hispanic	White	0.000	0.204	0.796	0.000
TCGA-13-0888	Ovary	Non-Hispanic	White	0.000	0.380	0.620	0.000
TCGA-13-0893	Ovary	Non-Hispanic	Black	0.099	0.209	0.321	0.371
TCGA-13-0897	Ovary	Non-Hispanic	White	0.028	0.074	0.506	0.392
TCGA-13-0900	Ovary	Non-Hispanic	White	0.000	0.435	0.000	0.565
TCGA-13-0901	Ovary	Non-Hispanic	Asian	0.015	0.330	0.000	0.655
TCGA-13-0905	Ovary	Non-Hispanic	White	0.816	0.000	0.000	0.184
TCGA-13-0906	Ovary	Non-Hispanic	White	0.177	0.305	0.429	0.090
TCGA-13-0908	Ovary	Non-Hispanic	White	1.000	0.000	0.000	0.000

TCGA-13-0911	Ovary	Non-Hispanic	White	0.015	0.150	0.437	0.398
TCGA-13-0913	Ovary	Non-Hispanic	White	0.000	0.085	0.512	0.403
TCGA-13-0916	Ovary	Non-Hispanic	White	0.391	0.150	0.459	0.000
TCGA-13-0920	Ovary	Non-Hispanic	White	0.068	0.387	0.000	0.545
TCGA-13-0923	Ovary	Non-Hispanic	White	0.000	0.122	0.878	0.000
TCGA-13-0924	Ovary	Non-Hispanic	White	0.144	0.000	0.169	0.687
TCGA-13-1403	Ovary	Non-Hispanic	White	0.159	0.249	0.592	0.000
TCGA-13-1404	Ovary	Non-Hispanic	White	0.370	0.083	0.454	0.093
TCGA-13-1405	Ovary	Non-Hispanic	White	0.144	0.081	0.775	0.000
TCGA-13-1407	Ovary	Non-Hispanic	White	0.000	1.000	0.000	0.000
TCGA-13-1408	Ovary	Non-Hispanic	White	0.499	0.501	0.000	0.000
TCGA-13-1409	Ovary	Non-Hispanic	White	0.548	0.086	0.366	0.000
TCGA-13-1410	Ovary	Non-Hispanic	White	0.101	0.166	0.540	0.193
TCGA-13-1411	Ovary	Non-Hispanic	White	0.496	0.267	0.000	0.236
TCGA-13-1477	Ovary	Non-Hispanic	White	0.563	0.000	0.437	0.000
TCGA-13-1483	Ovary	Non-Hispanic	White	0.072	0.542	0.260	0.126
TCGA-13-1485	Ovary	Non-Hispanic	White	0.431	0.000	0.000	0.569
TCGA-13-1487	Ovary	Non-Hispanic	White	0.000	0.390	0.610	0.000
TCGA-13-1488	Ovary	Non-Hispanic	White	0.055	0.258	0.461	0.226
TCGA-13-1489	Ovary	Non-Hispanic	White	0.251	0.269	0.441	0.039
TCGA-13-1492	Ovary	Non-Hispanic	White	0.000	0.645	0.000	0.355

TCGA-13-1495	Ovary	Non-Hispanic	White	0.375	0.245	0.000	0.380
TCGA-13-1496	Ovary	Non-Hispanic	White	0.101	0.336	0.563	0.000
TCGA-13-1497	Ovary	Non-Hispanic	White	0.000	0.319	0.000	0.681
TCGA-13-1498	Ovary	Non-Hispanic	White	0.427	0.387	0.186	0.000
TCGA-13-1499	Ovary	Non-Hispanic	White	0.216	0.371	0.000	0.413
TCGA-13-1501	Ovary	Non-Hispanic	White	0.000	0.304	0.696	0.000
TCGA-13-1505	Ovary	Non-Hispanic	White	0.000	0.401	0.599	0.000
TCGA-13-1506	Ovary	Non-Hispanic	White	0.142	0.380	0.000	0.478
TCGA-13-1509	Ovary	Non-Hispanic	White	0.454	0.000	0.000	0.546
TCGA-13-1510	Ovary	Non-Hispanic	White	0.272	0.031	0.442	0.255
TCGA-13-1511	Ovary	Non-Hispanic	Asian	0.019	0.323	0.305	0.353
TCGA-13-1512	Ovary	Non-Hispanic	White	0.042	0.240	0.413	0.305
TCGA-13-2060	Ovary	Unknown	White	0.079	0.309	0.263	0.349
TCGA-13-A5FT	Ovary	Unknown	Black	0.038	0.573	0.000	0.389
TCGA-20-0987	Ovary	Non-Hispanic	White	0.000	0.140	0.498	0.363
TCGA-20-0991	Ovary	Non-Hispanic	White	0.000	0.214	0.279	0.507
TCGA-20-1682	Ovary	Non-Hispanic	White	0.025	0.082	0.893	0.000
TCGA-20-1683	Ovary	Non-Hispanic	White	0.544	0.000	0.000	0.456
TCGA-20-1686	Ovary	Non-Hispanic	White	0.001	0.219	0.552	0.228
TCGA-20-1687	Ovary	Non-Hispanic	White	0.017	0.091	0.892	0.000
TCGA-23-1021	Ovary	Non-Hispanic	White	0.000	0.040	0.446	0.514
TCGA-23-1022	Ovary	Non-Hispanic	White	0.000	0.249	0.411	0.340

TCGA-23-1023	Ovary	Non-Hispanic	White	0.113	0.310	0.558	0.019
TCGA-23-1024	Ovary	Non-Hispanic	White	0.435	0.447	0.000	0.118
TCGA-23-1026	Ovary	Non-Hispanic	White	0.261	0.165	0.179	0.396
TCGA-23-1027	Ovary	Non-Hispanic	White	0.393	0.000	0.607	0.000
TCGA-23-1028	Ovary	Hispanic	White	0.071	0.145	0.000	0.784
TCGA-23-1029	Ovary	Non-Hispanic	White	0.084	0.171	0.745	0.000
TCGA-23-1030	Ovary	Non-Hispanic	White	0.040	0.268	0.161	0.530
TCGA-23-1107	Ovary	Non-Hispanic	White	0.000	0.055	0.352	0.594
TCGA-23-1109	Ovary	Non-Hispanic	White	0.382	0.104	0.000	0.514
TCGA-23-1110	Ovary	Hispanic	White	0.000	0.149	0.000	0.851
TCGA-23-1111	Ovary	Unknown	White	0.319	0.169	0.121	0.392
TCGA-23-1113	Ovary	Non-Hispanic	White	0.000	0.140	0.349	0.512
TCGA-23-1114	Ovary	Non-Hispanic	White	0.000	1.000	0.000	0.000
TCGA-23-1116	Ovary	Non-Hispanic	White	0.000	0.000	1.000	0.000
TCGA-23-1118	Ovary	Non-Hispanic	White	0.349	0.468	0.000	0.183
TCGA-23-1119	Ovary	Non-Hispanic	White	0.011	0.194	0.368	0.427
TCGA-23-1120	Ovary	Non-Hispanic	White	0.565	0.251	0.000	0.184
TCGA-23-1123	Ovary	Non-Hispanic	White	0.112	0.268	0.282	0.339
TCGA-23-1809	Ovary	Non-Hispanic	White	0.000	0.897	0.000	0.103
TCGA-23-2077	Ovary	Non-Hispanic	White	0.000	0.513	0.487	0.000
TCGA-23-2078	Ovary	Non-Hispanic	White	0.410	0.329	0.000	0.262
TCGA-23-2084	Ovary	Non-Hispanic	White	0.196	0.188	0.005	0.611
TCGA-24-0966	Ovary	Non-Hispanic	Black	0.007	0.236	0.450	0.308

TCGA-24-0968	Ovary	Unknown	White	0.000	0.467	0.217	0.315
TCGA-24-0970	Ovary	Unknown	White	0.000	0.228	0.772	0.000
TCGA-24-0979	Ovary	Unknown	White	0.013	0.288	0.699	0.000
TCGA-24-0982	Ovary	Unknown	White	0.255	0.092	0.594	0.059
TCGA-24-1103	Ovary	Unknown	Black	0.000	0.222	0.176	0.602
TCGA-24-1104	Ovary	Unknown	White	0.152	0.319	0.529	0.000
TCGA-24-1105	Ovary	Unknown	White	0.113	0.697	0.190	0.000
TCGA-24-1413	Ovary	Unknown	White	0.277	0.185	0.000	0.537
TCGA-24-1416	Ovary	Unknown	White	0.000	0.167	0.187	0.646
TCGA-24-1417	Ovary	Unknown	White	0.340	0.000	0.660	0.000
TCGA-24-1418	Ovary	Unknown	White	0.564	0.000	0.436	0.000
TCGA-24-1419	Ovary	Unknown	White	0.097	0.321	0.330	0.252
TCGA-24-1422	Ovary	Unknown	Black	0.186	0.462	0.351	0.000
TCGA-24-1423	Ovary	Unknown	White	0.000	0.516	0.483	0.001
TCGA-24-1425	Ovary	Unknown	White	0.360	0.450	0.190	0.000
TCGA-24-1426	Ovary	Unknown	White	1.000	0.000	0.000	0.000
TCGA-24-1427	Ovary	Unknown	Unknown	0.734	0.000	0.000	0.266
TCGA-24-1428	Ovary	Unknown	White	0.340	0.660	0.000	0.000
TCGA-24-1430	Ovary	Unknown	White	1.000	0.000	0.000	0.000
TCGA-24-1431	Ovary	Unknown	White	0.000	0.072	0.000	0.928
TCGA-24-1434	Ovary	Unknown	White	0.000	0.663	0.337	0.000
TCGA-24-1464	Ovary	Unknown	White	0.000	0.321	0.679	0.000
TCGA-24-1467	Ovary	Unknown	White	0.092	0.151	0.196	0.560
TCGA-24-1469	Ovary	Unknown	White	0.360	0.542	0.000	0.098
TCGA-24-1470	Ovary	Unknown	White	1.000	0.000	0.000	0.000
TCGA-24-1471	Ovary	Unknown	White	0.029	0.371	0.000	0.600
TCGA-24-1474	Ovary	Unknown	Black	0.141	0.290	0.254	0.315
TCGA-24-1544	Ovary	Unknown	Black	0.000	0.135	0.076	0.789
TCGA-24-1546	Ovary	Unknown	White	0.288	0.425	0.287	0.000
TCGA-24-1550	Ovary	Unknown	White	0.000	0.733	0.000	0.267
TCGA-24-1551	Ovary	Unknown	White	0.153	0.099	0.441	0.307
TCGA-24-1552	Ovary	Unknown	White	0.073	0.140	0.376	0.411
TCGA-24-1553	Ovary	Unknown	White	0.155	0.507	0.082	0.256
TCGA-24-1557	Ovary	Unknown	White	0.113	0.000	0.301	0.586
TCGA-24-1560	Ovary	Unknown	White	0.414	0.265	0.321	0.000
TCGA-24-1562	Ovary	Unknown	White	0.000	0.223	0.433	0.344
TCGA-24-1563	Ovary	Unknown	Black	0.000	0.364	0.340	0.296
TCGA-24-1565	Ovary	Unknown	White	0.315	0.547	0.138	0.000
TCGA-24-1567	Ovary	Unknown	White	0.041	0.354	0.293	0.313
TCGA-24-1603	Ovary	Unknown	White	0.142	0.339	0.519	0.000
TCGA-24-1604	Ovary	Unknown	White	0.736	0.000	0.264	0.000
TCGA-24-1616	Ovary	Unknown	White	0.000	0.106	0.328	0.566

TCGA-24-1842	Ovary	Unknown	White	0.272	0.196	0.248	0.284
TCGA-24-1843	Ovary	Unknown	White	0.000	0.862	0.138	0.000
TCGA-24-1844	Ovary	Unknown	White	0.154	0.538	0.308	0.000
TCGA-24-1846	Ovary	Unknown	Unknown	0.217	0.362	0.000	0.421
TCGA-24-1847	Ovary	Unknown	White	0.110	0.327	0.563	0.000
TCGA-24-1850	Ovary	Unknown	White	0.000	0.233	0.761	0.005
TCGA-24-1923	Ovary	Unknown	White	0.142	0.496	0.000	0.362
TCGA-24-1924	Ovary	Unknown	White	0.114	0.206	0.680	0.000
TCGA-24-1928	Ovary	Unknown	White	0.241	0.264	0.065	0.429
TCGA-24-1930	Ovary	Unknown	White	0.000	0.308	0.479	0.212
TCGA-24-2020	Ovary	Unknown	White	0.158	0.097	0.397	0.349
TCGA-24-2023	Ovary	Unknown	White	0.059	0.441	0.366	0.134
TCGA-24-2024	Ovary	Unknown	Black	0.562	0.438	0.000	0.000
TCGA-24-2026	Ovary	Unknown	White	0.000	1.000	0.000	0.000
TCGA-24-2027	Ovary	Unknown	White	0.693	0.000	0.307	0.000
TCGA-24-2033	Ovary	Unknown	White	0.068	0.270	0.662	0.000
TCGA-24-2035	Ovary	Unknown	White	0.000	0.317	0.369	0.313
TCGA-24-2036	Ovary	Unknown	White	1.000	0.000	0.000	0.000
TCGA-24-2038	Ovary	Unknown	White	0.513	0.072	0.000	0.416
TCGA-24-2254	Ovary	Unknown	White	0.303	0.237	0.000	0.460
TCGA-24-2267	Ovary	Unknown	White	0.312	0.000	0.688	0.000
TCGA-24-2271	Ovary	Unknown	Asian	0.078	0.025	0.644	0.254
TCGA-24-2280	Ovary	Unknown	White	0.000	1.000	0.000	0.000
TCGA-24-2288	Ovary	Unknown	White	0.000	0.284	0.716	0.000
TCGA-24-2289	Ovary	Unknown	White	0.102	0.642	0.000	0.256
TCGA-24-2290	Ovary	Unknown	White	0.229	0.053	0.472	0.246
TCGA-24-2293	Ovary	Unknown	White	0.108	0.892	0.000	0.000
TCGA-24-2297	Ovary	Unknown	White	0.464	0.472	0.064	0.000
TCGA-24-2298	Ovary	Unknown	White	0.680	0.000	0.000	0.320
TCGA-25-1312	Ovary	Non-Hispanic	White	0.133	0.204	0.293	0.370
TCGA-25-1313	Ovary	Non-Hispanic	White	0.177	0.000	0.362	0.461
TCGA-25-1315	Ovary	Non-Hispanic	White	0.589	0.251	0.000	0.160
TCGA-25-1316	Ovary	Non-Hispanic	White	0.315	0.103	0.126	0.456
TCGA-25-1318	Ovary	Non-Hispanic	White	0.128	0.198	0.000	0.673
TCGA-25-1319	Ovary	Non-Hispanic	White	0.000	0.218	0.782	0.000

TCGA-25-1320	Ovary	Non-Hispanic	White	0.342	0.658	0.000	0.000
TCGA-25-1321	Ovary	Non-Hispanic	White	0.298	0.403	0.299	0.000
TCGA-25-1322	Ovary	Non-Hispanic	White	0.098	0.299	0.399	0.203
TCGA-25-1323	Ovary	Non-Hispanic	White	0.256	0.743	0.000	0.000
TCGA-25-1326	Ovary	Non-Hispanic	White	0.263	0.202	0.000	0.535
TCGA-25-1328	Ovary	Non-Hispanic	White	0.526	0.054	0.000	0.420
TCGA-25-1329	Ovary	Non-Hispanic	White	0.388	0.549	0.000	0.063
TCGA-25-1623	Ovary	Non-Hispanic	White	0.281	0.000	0.706	0.013
TCGA-25-1626	Ovary	Non-Hispanic	White	0.458	0.335	0.206	0.000
TCGA-25-1627	Ovary	Non-Hispanic	White	0.000	0.190	0.810	0.000
TCGA-25-1628	Ovary	Non-Hispanic	White	0.748	0.000	0.000	0.252
TCGA-25-1630	Ovary	Non-Hispanic	White	0.114	0.133	0.597	0.157
TCGA-25-1631	Ovary	Non-Hispanic	White	0.237	0.300	0.000	0.464
TCGA-25-1633	Ovary	Non-Hispanic	White	0.121	0.261	0.505	0.114
TCGA-25-1634	Ovary	Non-Hispanic	White	0.000	0.337	0.491	0.172
TCGA-25-1635	Ovary	Non-Hispanic	White	0.286	0.275	0.440	0.000
TCGA-25-1870	Ovary	Non-Hispanic	White	0.629	0.000	0.240	0.131
TCGA-25-1877	Ovary	Non-Hispanic	White	0.049	0.439	0.512	0.000
TCGA-25-2042	Ovary	Non-Hispanic	American Indian	0.000	0.537	0.345	0.118
TCGA-25-2391	Ovary	Non-Hispanic	White	0.130	0.487	0.383	0.000
TCGA-25-2392	Ovary	Non-Hispanic	White	0.059	0.290	0.397	0.254

TCGA-25-2393	Ovary	Non-Hispanic	White	0.000	0.389	0.000	0.611
TCGA-25-2396	Ovary	Non-Hispanic	White	0.052	0.263	0.686	0.000
TCGA-25-2398	Ovary	Non-Hispanic	White	0.188	0.175	0.423	0.214
TCGA-25-2399	Ovary	Non-Hispanic	White	0.358	0.415	0.042	0.184
TCGA-25-2400	Ovary	Non-Hispanic	White	0.028	0.190	0.781	0.000
TCGA-25-2401	Ovary	Non-Hispanic	White	0.000	0.247	0.000	0.753
TCGA-25-2404	Ovary	Non-Hispanic	American Indian	0.169	0.341	0.370	0.120
TCGA-29-1688	Ovary	Unknown	White	0.335	0.000	0.461	0.203
TCGA-29-1690	Ovary	Unknown	White	0.093	0.203	0.504	0.200
TCGA-29-1691	Ovary	Unknown	White	0.012	0.380	0.608	0.000
TCGA-29-1693	Ovary	Unknown	White	0.052	0.269	0.401	0.279
TCGA-29-1694	Ovary	Unknown	White	0.538	0.233	0.229	0.000
TCGA-29-1695	Ovary	Unknown	White	0.242	0.185	0.573	0.000
TCGA-29-1696	Ovary	Unknown	White	0.120	0.177	0.478	0.224
TCGA-29-1697	Ovary	Unknown	White	0.471	0.000	0.529	0.000
TCGA-29-1701	Ovary	Unknown	White	0.034	0.168	0.436	0.362
TCGA-29-1703	Ovary	Unknown	Black	0.000	0.246	0.258	0.496
TCGA-29-1705	Ovary	Unknown	White	0.134	0.135	0.731	0.000
TCGA-29-1707	Ovary	Unknown	White	0.061	0.321	0.229	0.389
TCGA-29-1710	Ovary	Unknown	White	0.057	0.374	0.242	0.327
TCGA-29-1711	Ovary	Unknown	Black	0.000	0.240	0.760	0.000
TCGA-29-1761	Ovary	Unknown	Asian	0.000	0.324	0.031	0.646
TCGA-29-1762	Ovary	Unknown	White	0.338	0.214	0.448	0.000
TCGA-29-1763	Ovary	Unknown	Black	0.591	0.195	0.000	0.214
TCGA-29-1766	Ovary	Unknown	White	0.000	0.373	0.001	0.625
TCGA-29-1768	Ovary	Unknown	White	0.133	0.000	0.867	0.000
TCGA-29-1769	Ovary	Unknown	White	0.000	0.180	0.098	0.722
TCGA-29-1770	Ovary	Unknown	White	0.000	0.219	0.429	0.352
TCGA-29-1774	Ovary	Non-Hispanic	White	0.064	0.153	0.617	0.166
TCGA-29-1776	Ovary	Unknown	Black	0.000	0.229	0.771	0.000
TCGA-29-1777	Ovary	Unknown	White	0.000	0.177	0.217	0.606
TCGA-29-1778	Ovary	Unknown	White	0.256	0.278	0.000	0.466
TCGA-29-1781	Ovary	Unknown	White	0.089	0.159	0.751	0.000
TCGA-29-1783	Ovary	Unknown	White	0.071	0.186	0.379	0.364

TCGA-29-1784	Ovary	Unknown	White	0.000	0.378	0.622	0.000
TCGA-29-1785	Ovary	Unknown	White	0.000	0.207	0.772	0.020
TCGA-29-2414	Ovary	Unknown	White	0.263	0.737	0.000	0.000
TCGA-29-2425	Ovary	Unknown	White	0.000	0.231	0.769	0.000
TCGA-29-2428	Ovary	Unknown	White	0.000	0.334	0.666	0.000
TCGA-29-A5NZ	Ovary	Unknown	Black	0.476	0.473	0.000	0.051
TCGA-30-1714	Ovary	Non-Hispanic	White	0.000	0.293	0.484	0.223
TCGA-30-1718	Ovary	Non-Hispanic	White	0.000	0.271	0.089	0.640
TCGA-30-1853	Ovary	Non-Hispanic	White	0.076	0.086	0.661	0.176
TCGA-30-1857	Ovary	Non-Hispanic	White	0.094	0.180	0.472	0.254
TCGA-30-1860	Ovary	Hispanic	White	0.191	0.000	0.506	0.303
TCGA-30-1861	Ovary	Non-Hispanic	White	0.098	0.345	0.557	0.000
TCGA-30-1862	Ovary	Non-Hispanic	White	0.288	0.299	0.000	0.413
TCGA-30-1866	Ovary	Non-Hispanic	White	0.159	0.380	0.418	0.043
TCGA-30-1891	Ovary	Non-Hispanic	White	0.048	0.045	0.908	0.000
TCGA-30-1892	Ovary	Non-Hispanic	White	0.000	0.061	0.939	0.000
TCGA-31-1944	Ovary	Non-Hispanic	White	0.000	0.192	0.000	0.808
TCGA-31-1946	Ovary	Unknown	Unknown	0.000	0.339	0.661	0.000
TCGA-31-1950	Ovary	Non-Hispanic	White	0.000	0.756	0.244	0.000
TCGA-31-1951	Ovary	Non-Hispanic	White	0.457	0.000	0.543	0.000
TCGA-31-1953	Ovary	Non-Hispanic	Asian	0.226	0.171	0.603	0.000
TCGA-31-1956	Ovary	Non-Hispanic	White	0.028	0.191	0.781	0.000
TCGA-31-1959	Ovary	Non-Hispanic	White	0.035	0.233	0.000	0.731
TCGA-36-1568	Ovary	Unknown	Unknown	0.087	0.375	0.000	0.538
TCGA-36-1569	Ovary	Unknown	White	0.050	0.409	0.497	0.044
TCGA-36-1570	Ovary	Unknown	White	0.554	0.293	0.008	0.145
TCGA-36-1571	Ovary	Unknown	White	0.083	0.280	0.327	0.310

TCGA-36-1574	Ovary	Unknown	Asian	0.535	0.000	0.000	0.465
TCGA-36-1576	Ovary	Unknown	Unknown	0.015	0.310	0.321	0.354
TCGA-36-1577	Ovary	Unknown	Asian	0.572	0.000	0.293	0.135
TCGA-36-1580	Ovary	Unknown	Unknown	0.181	0.242	0.577	0.000
TCGA-36-1581	Ovary	Unknown	White	0.045	0.723	0.232	0.000
TCGA-57-1582	Ovary	Unknown	White	0.197	0.171	0.146	0.486
TCGA-57-1583	Ovary	Unknown	Black	0.000	0.359	0.000	0.641
TCGA-57-1584	Ovary	Non-Hispanic	White	0.172	0.007	0.390	0.431
TCGA-57-1585	Ovary	Non-Hispanic	White	0.116	0.683	0.201	0.000
TCGA-57-1586	Ovary	Non-Hispanic	White	0.000	0.399	0.601	0.000
TCGA-57-1994	Ovary	Unknown	White	0.038	0.815	0.000	0.148
TCGA-59-2350	Ovary	Unknown	Unknown	0.658	0.000	0.342	0.000
TCGA-59-2351	Ovary	Non-Hispanic	White	0.100	0.445	0.000	0.455
TCGA-59-2354	Ovary	Non-Hispanic	White	0.095	0.379	0.348	0.177
TCGA-59-2355	Ovary	Non-Hispanic	White	0.206	0.197	0.597	0.000
TCGA-59-2363	Ovary	Non-Hispanic	Asian	0.240	0.283	0.083	0.395
TCGA-59-A5PD	Ovary	Non-Hispanic	Black	0.204	0.145	0.367	0.283
TCGA-5X-AA5U	Ovary	Unknown	Black	0.000	0.885	0.115	0.000
TCGA-61-1721	Ovary	Non-Hispanic	White	0.000	0.210	0.410	0.380
TCGA-61-1724	Ovary	Non-Hispanic	White	0.255	0.236	0.060	0.449
TCGA-61-1725	Ovary	Non-Hispanic	White	0.123	0.090	0.460	0.327
TCGA-61-1728	Ovary	Non-Hispanic	White	0.000	0.561	0.439	0.000
TCGA-61-1736	Ovary	Non-Hispanic	White	0.263	0.071	0.341	0.326
TCGA-61-1737	Ovary	Non-Hispanic	White	0.293	0.217	0.376	0.114
TCGA-61-1738	Ovary	Unknown	Black	0.000	0.378	0.622	0.000
TCGA-61-1741	Ovary	Non-Hispanic	White	0.562	0.167	0.000	0.270

TCGA-61-1900	Ovary	Non-Hispanic	Black	0.332	0.528	0.140	0.000
TCGA-61-1907	Ovary	Non-Hispanic	White	0.185	0.000	0.529	0.286
TCGA-61-1910	Ovary	Non-Hispanic	White	0.090	0.292	0.109	0.508
TCGA-61-1911	Ovary	Non-Hispanic	White	0.000	0.046	0.272	0.682
TCGA-61-1914	Ovary	Unknown	Unknown	0.037	0.250	0.047	0.667
TCGA-61-1918	Ovary	Non-Hispanic	White	0.020	0.328	0.652	0.000
TCGA-61-1919	Ovary	Non-Hispanic	White	0.000	0.334	0.279	0.387
TCGA-61-1995	Ovary	Non-Hispanic	White	0.200	0.386	0.414	0.000
TCGA-61-1998	Ovary	Non-Hispanic	White	0.090	0.256	0.479	0.174
TCGA-61-2000	Ovary	Non-Hispanic	White	0.000	0.270	0.730	0.000
TCGA-61-2002	Ovary	Non-Hispanic	White	0.054	0.461	0.298	0.188
TCGA-61-2003	Ovary	Non-Hispanic	White	0.241	0.250	0.000	0.510
TCGA-61-2008	Ovary	Non-Hispanic	Asian	0.503	0.000	0.497	0.000
TCGA-61-2009	Ovary	Non-Hispanic	White	0.071	0.273	0.357	0.300
TCGA-61-2012	Ovary	Non-Hispanic	White	0.000	0.723	0.134	0.143
TCGA-61-2088	Ovary	Non-Hispanic	White	0.000	0.388	0.532	0.080
TCGA-61-2092	Ovary	Unknown	White	0.000	0.368	0.000	0.632
TCGA-61-2097	Ovary	Unknown	White	0.213	0.000	0.041	0.746
TCGA-61-2098	Ovary	Unknown	White	0.000	1.000	0.000	0.000
TCGA-61-2101	Ovary	Non-Hispanic	White	0.000	0.656	0.344	0.000
TCGA-61-2102	Ovary	Unknown	White	0.000	0.170	0.370	0.460
TCGA-61-2104	Ovary	Unknown	White	0.000	0.627	0.000	0.373
TCGA-61-2113	Ovary	Non-Hispanic	White	0.000	0.582	0.000	0.418
TCGA-OY-A56Q	Ovary	Non-Hispanic	Black	0.302	0.142	0.000	0.556

TCGA-VG-A8LO	Ovary	Non-Hispanic	Black	0.350	0.300	0.000	0.351
TCGA-WR-A838	Ovary	Non-Hispanic	Black	0.748	0.000	0.000	0.252

Table 2. Q-matrix with 6 cluster solution at the subpopulations structure analysis of all TCGA patients. For each patient there is also information about tumor type and self-declared race and ethnicity.

FID	Tumor type	Ethnicity	Race	Cluster 1	Cluster 2	Cluster 3	Cluster 4	Cluster 5	Cluster 6
TCGA-2E-A9G8	Endometrial	Non-Hispanic	Black	0.000	0.000	0.000	0.965	0.000	0.035
TCGA-4E-A92E	Endometrial	Non-Hispanic	Black	0.454	0.000	0.106	0.440	0.000	0.000
TCGA-5B-A90C	Endometrial	Non-Hispanic	Black	1.000	0.000	0.000	0.000	0.000	0.000
TCGA-A5-A0G9	Endometrial	Unknown	White	0.299	0.000	0.001	0.526	0.173	0.000
TCGA-A5-A0GA	Endometrial	Non-Hispanic	White	0.629	0.000	0.000	0.041	0.329	0.000
TCGA-A5-A0GB	Endometrial	Unknown	White	0.608	0.000	0.000	0.135	0.257	0.000
TCGA-A5-A0GD	Endometrial	Unknown	White	0.632	0.000	0.000	0.368	0.000	0.000
TCGA-A5-A0GE	Endometrial	Non-Hispanic	Asian	0.230	0.000	0.000	0.770	0.000	0.000
TCGA-A5-A0GG	Endometrial	Non-Hispanic	Black	0.372	0.000	0.000	0.014	0.614	0.000
TCGA-A5-A0GH	Endometrial	Non-Hispanic	White	0.000	0.000	0.000	0.333	0.667	0.000
TCGA-A5-A0GI	Endometrial	Non-Hispanic	White	0.370	0.000	0.000	0.046	0.584	0.000
TCGA-A5-A0GJ	Endometrial	Non-Hispanic	White	0.888	0.000	0.000	0.000	0.112	0.000
TCGA-A5-A0GM	Endometrial	Non-Hispanic	White	0.000	0.068	0.000	0.512	0.420	0.000
TCGA-A5-A0GN	Endometrial	Non-Hispanic	White	0.181	0.000	0.000	0.377	0.442	0.000
TCGA-A5-A0GP	Endometrial	Non-Hispanic	Asian	0.000	0.000	0.000	0.505	0.495	0.000
TCGA-A5-A0GQ	Endometrial	Non-Hispanic	White	0.392	0.000	0.000	0.361	0.246	0.000
TCGA-A5-A0GR	Endometrial	Non-Hispanic	Black	0.000	0.016	0.000	0.190	0.794	0.000
TCGA-A5-A0GU	Endometrial	Unknown	White	0.164	0.000	0.008	0.236	0.592	0.000
TCGA-A5-A0GV	Endometrial	Non-Hispanic	White	0.310	0.000	0.000	0.186	0.504	0.000
TCGA-A5-A0GW	Endometrial	Non-Hispanic	Asian	0.436	0.000	0.000	0.000	0.564	0.000
TCGA-A5-A0GX	Endometrial	Unknown	White	0.677	0.000	0.000	0.323	0.000	0.000
TCGA-A5-A0R7	Endometrial	Non-Hispanic	White	1.000	0.000	0.000	0.000	0.000	0.000
TCGA-A5-A0R8	Endometrial	Non-Hispanic	White	0.679	0.000	0.000	0.321	0.000	0.000
TCGA-A5-A0R9	Endometrial	Non-Hispanic	White	0.000	0.000	0.016	0.433	0.551	0.000
TCGA-A5-A0RA	Endometrial	Non-Hispanic	White	0.000	0.166	0.000	0.834	0.000	0.000
TCGA-A5-A0VO	Endometrial	Non-Hispanic	White	0.000	0.000	0.000	0.699	0.300	0.000
TCGA-A5-A0VP	Endometrial	Non-Hispanic	White	0.512	0.000	0.000	0.000	0.488	0.000
TCGA-A5-A0VQ	Endometrial	Hispanic	Unknown	0.485	0.046	0.000	0.469	0.000	0.000
TCGA-A5-A1OJ	Endometrial	Non-Hispanic	Asian	0.367	0.032	0.000	0.393	0.208	0.000
TCGA-A5-A1OK	Endometrial	Non-Hispanic	White	0.355	0.000	0.000	0.645	0.000	0.000
TCGA-A5-A2K5	Endometrial	Non-Hispanic	White	0.294	0.000	0.000	0.000	0.706	0.000
TCGA-A5-A2K7	Endometrial	Hispanic	Unknown	0.349	0.000	0.001	0.273	0.377	0.000

TCGA-A5-A3LO	Endometrial	Non-Hispanic	White	0.000	0.000	0.114	0.000	0.886	0.000
TCGA-A5-A7WJ	Endometrial	Non-Hispanic	Black	0.504	0.000	0.000	0.115	0.380	0.000
TCGA-A5-AB3J	Endometrial	Non-Hispanic	Black	1.000	0.000	0.000	0.000	0.000	0.000
TCGA-AJ-A23O	Endometrial	Non-Hispanic	White	0.341	0.000	0.016	0.205	0.438	0.000
TCGA-AJ-A2QK	Endometrial	Hispanic	White	0.000	0.000	0.000	0.000	1.000	0.000
TCGA-AJ-A2QL	Endometrial	Non-Hispanic	White	1.000	0.000	0.000	0.000	0.000	0.000
TCGA-AJ-A2QN	Endometrial	Unknown	White	0.540	0.000	0.000	0.000	0.381	0.079
TCGA-AJ-A2QO	Endometrial	Unknown	White	0.328	0.000	0.000	0.271	0.401	0.000
TCGA-AJ-A3BH	Endometrial	Non-Hispanic	White	1.000	0.000	0.000	0.000	0.000	0.000
TCGA-AJ-A3BI	Endometrial	Non-Hispanic	White	0.000	0.000	0.000	0.000	1.000	0.000
TCGA-AJ-A3BK	Endometrial	Non-Hispanic	White	0.271	0.000	0.023	0.365	0.341	0.000
TCGA-AJ-A3EK	Endometrial	Hispanic	White	0.740	0.000	0.000	0.104	0.156	0.000
TCGA-AJ-A3EL	Endometrial	Unknown	Pacific	0.358	0.000	0.037	0.367	0.238	0.000
TCGA-AJ-A3EM	Endometrial	Unknown	White	0.543	0.000	0.000	0.000	0.339	0.118
TCGA-AJ-A3I9	Endometrial	Non-Hispanic	White	0.295	0.000	0.019	0.380	0.306	0.000
TCGA-AJ-A3NC	Endometrial	Unknown	White	0.627	0.000	0.195	0.067	0.111	0.000
TCGA-AJ-A3NE	Endometrial	Non-Hispanic	White	0.000	0.000	0.000	0.924	0.000	0.076
TCGA-AJ-A3OJ	Endometrial	Non-Hispanic	White	1.000	0.000	0.000	0.000	0.000	0.000
TCGA-AJ-A3OL	Endometrial	Non-Hispanic	White	0.759	0.000	0.168	0.000	0.073	0.000
TCGA-AJ-A5DV	Endometrial	Non-Hispanic	Black	0.492	0.000	0.066	0.055	0.387	0.000
TCGA-AJ-A8CT	Endometrial	Unknown	Black	0.352	0.000	0.019	0.154	0.475	0.000
TCGA-AJ-A8CV	Endometrial	Unknown	Black	0.000	0.000	0.000	0.000	1.000	0.000
TCGA-AJ-A8CW	Endometrial	Non-Hispanic	Black	0.610	0.000	0.057	0.083	0.250	0.000
TCGA-AP-A051	Endometrial	Unknown	White	0.478	0.000	0.000	0.000	0.522	0.000
TCGA-AP-A053	Endometrial	Non-Hispanic	White	0.754	0.000	0.000	0.000	0.246	0.000
TCGA-AP-A054	Endometrial	Non-Hispanic	White	0.000	0.000	0.000	0.623	0.377	0.000
TCGA-AP-A056	Endometrial	Unknown	White	0.000	0.000	0.000	0.000	1.000	0.000
TCGA-AP-A059	Endometrial	Non-Hispanic	White	0.000	0.000	0.000	1.000	0.000	0.000
TCGA-AP-A05N	Endometrial	Unknown	White	0.327	0.000	0.000	0.119	0.554	0.000
TCGA-AP-A05O	Endometrial	Unknown	White	0.490	0.000	0.017	0.493	0.000	0.000
TCGA-AP-A05P	Endometrial	Non-Hispanic	Asian	0.000	0.000	0.000	0.478	0.522	0.000
TCGA-AP-A0LD	Endometrial	Unknown	White	0.179	0.000	0.000	0.000	0.821	0.000
TCGA-AP-A0LE	Endometrial	Unknown	White	0.000	0.000	0.000	0.000	1.000	0.000
TCGA-AP-A0LF	Endometrial	Non-Hispanic	White	0.000	0.469	0.000	0.531	0.000	0.000
TCGA-AP-A0LG	Endometrial	Unknown	White	0.000	0.000	0.000	1.000	0.000	0.000
TCGA-AP-A0LJ	Endometrial	Unknown	White	0.000	0.122	0.000	0.681	0.198	0.000
TCGA-AP-A0LL	Endometrial	Unknown	White	0.000	0.000	0.000	0.000	1.000	0.000
TCGA-AP-A0LM	Endometrial	Unknown	Black	0.000	0.249	0.010	0.428	0.313	0.000
TCGA-AP-A0LN	Endometrial	Unknown	Black	0.366	0.000	0.006	0.100	0.528	0.000
TCGA-AP-A0LO	Endometrial	Non-Hispanic	White	0.495	0.000	0.000	0.265	0.240	0.000
TCGA-AP-A0LP	Endometrial	Unknown	White	0.478	0.028	0.000	0.494	0.000	0.000
TCGA-AP-A0LS	Endometrial	Unknown	White	0.234	0.056	0.000	0.299	0.411	0.000

TCGA-AP-A0LT	Endometrial	Unknown	White	0.261	0.000	0.000	0.257	0.483	0.000
TCGA-AP-A0LV	Endometrial	Unknown	White	0.000	0.000	0.000	0.631	0.369	0.000
TCGA-AP-A1DH	Endometrial	Unknown	White	0.000	0.000	0.000	0.224	0.776	0.000
TCGA-AP-A1DK	Endometrial	Unknown	White	0.166	0.000	0.000	0.201	0.582	0.052
TCGA-AP-A1DM	Endometrial	Unknown	White	0.435	0.000	0.000	0.000	0.565	0.000
TCGA-AP-A1DO	Endometrial	Non-Hispanic	White	1.000	0.000	0.000	0.000	0.000	0.000
TCGA-AP-A1DP	Endometrial	Unknown	White	0.332	0.000	0.036	0.632	0.000	0.000
TCGA-AP-A1DR	Endometrial	Unknown	Black	0.423	0.000	0.000	0.000	0.577	0.000
TCGA-AP-A1DV	Endometrial	Non-Hispanic	White	0.000	0.000	0.000	0.000	1.000	0.000
TCGA-AP-A1E0	Endometrial	Unknown	White	0.334	0.218	0.000	0.000	0.447	0.000
TCGA-AP-A1E1	Endometrial	Unknown	White	0.158	0.000	0.000	0.374	0.467	0.000
TCGA-AP-A1E3	Endometrial	Unknown	White	0.513	0.019	0.000	0.252	0.215	0.000
TCGA-AP-A1E4	Endometrial	Unknown	White	0.191	0.000	0.070	0.084	0.655	0.000
TCGA-AW-A1PO	Endometrial	Non-Hispanic	White	0.393	0.000	0.000	0.607	0.000	0.000
TCGA-AX-A05S	Endometrial	Non-Hispanic	White	0.169	0.000	0.000	0.508	0.323	0.000
TCGA-AX-A05T	Endometrial	Unknown	White	0.000	0.000	0.000	0.000	1.000	0.000
TCGA-AX-A05U	Endometrial	Non-Hispanic	American Indian	0.327	0.000	0.000	0.448	0.226	0.000
TCGA-AX-A05W	Endometrial	Non-Hispanic	White	0.635	0.000	0.000	0.365	0.000	0.000
TCGA-AX-A05Y	Endometrial	Non-Hispanic	White	0.389	0.000	0.000	0.410	0.201	0.000
TCGA-AX-A05Z	Endometrial	Hispanic	Unknown	0.263	0.000	0.000	0.287	0.451	0.000
TCGA-AX-A060	Endometrial	Non-Hispanic	American Indian	0.523	0.000	0.000	0.236	0.241	0.000
TCGA-AX-A062	Endometrial	Unknown	White	0.383	0.000	0.000	0.185	0.432	0.000
TCGA-AX-A063	Endometrial	Non-Hispanic	White	0.161	0.000	0.000	0.375	0.463	0.000
TCGA-AX-A064	Endometrial	Unknown	White	0.000	0.000	0.000	0.363	0.637	0.000
TCGA-AX-A06B	Endometrial	Non-Hispanic	White	0.672	0.000	0.000	0.203	0.124	0.000
TCGA-AX-A06D	Endometrial	Unknown	White	0.470	0.000	0.013	0.355	0.162	0.000
TCGA-AX-A06F	Endometrial	Non-Hispanic	White	0.000	0.000	0.000	0.358	0.642	0.000
TCGA-AX-A06H	Endometrial	Non-Hispanic	American Indian	0.000	0.000	0.000	0.553	0.447	0.000
TCGA-AX-A06J	Endometrial	Non-Hispanic	White	0.000	0.000	0.000	0.282	0.718	0.000
TCGA-AX-A06L	Endometrial	Unknown	White	0.000	0.283	0.000	0.619	0.082	0.015
TCGA-AX-A0IS	Endometrial	Non-Hispanic	White	0.027	0.356	0.000	0.617	0.000	0.000
TCGA-AX-A0IZ	Endometrial	Non-Hispanic	White	0.000	0.000	0.000	0.436	0.554	0.010
TCGA-AX-A0J0	Endometrial	Non-Hispanic	White	0.309	0.058	0.140	0.230	0.263	0.000
TCGA-AX-A0J1	Endometrial	Non-Hispanic	White	0.198	0.000	0.000	0.530	0.272	0.000
TCGA-AX-A1C4	Endometrial	Non-Hispanic	White	0.000	0.026	0.000	0.000	0.974	0.000
TCGA-AX-A1C5	Endometrial	Non-Hispanic	White	0.000	0.000	0.000	1.000	0.000	0.000
TCGA-AX-A1C9	Endometrial	Non-Hispanic	White	0.137	0.000	0.000	0.393	0.470	0.000
TCGA-AX-A1CE	Endometrial	Non-Hispanic	White	0.222	0.000	0.033	0.370	0.375	0.000
TCGA-AX-A1CF	Endometrial	Non-Hispanic	White	0.315	0.000	0.026	0.168	0.491	0.000

TCGA-AX-A1CI	Endometrial	Non-Hispanic	White	0.000	0.230	0.000	0.223	0.547	0.000
TCGA-AX-A1CJ	Endometrial	Non-Hispanic	Black	0.455	0.000	0.000	0.119	0.426	0.000
TCGA-AX-A1CK	Endometrial	Non-Hispanic	White	0.871	0.000	0.098	0.030	0.000	0.000
TCGA-AX-A1CN	Endometrial	Non-Hispanic	White	0.447	0.000	0.000	0.000	0.553	0.000
TCGA-AX-A2H7	Endometrial	Non-Hispanic	White	0.662	0.031	0.006	0.302	0.000	0.000
TCGA-AX-A2H8	Endometrial	Non-Hispanic	White	0.498	0.000	0.020	0.222	0.252	0.009
TCGA-AX-A2HA	Endometrial	Unknown	White	0.000	0.000	0.013	0.000	0.987	0.000
TCGA-AX-A2HC	Endometrial	Non-Hispanic	Black	0.000	0.000	0.000	0.446	0.554	0.000
TCGA-AX-A2HD	Endometrial	Non-Hispanic	White	0.000	0.000	0.016	0.984	0.000	0.000
TCGA-AX-A2HG	Endometrial	Hispanic	White	0.422	0.020	0.000	0.340	0.219	0.000
TCGA-AX-A2HH	Endometrial	Non-Hispanic	White	0.273	0.000	0.000	0.000	0.727	0.000
TCGA-AX-A2HJ	Endometrial	Non-Hispanic	Asian	0.383	0.000	0.000	0.175	0.442	0.000
TCGA-AX-A2HK	Endometrial	Unknown	White	0.233	0.000	0.000	0.472	0.294	0.000
TCGA-AX-A2IN	Endometrial	Unknown	White	0.137	0.000	0.000	0.210	0.653	0.000
TCGA-AX-A3FS	Endometrial	Non-Hispanic	White	0.633	0.000	0.009	0.000	0.357	0.000
TCGA-AX-A3FT	Endometrial	Non-Hispanic	White	1.000	0.000	0.000	0.000	0.000	0.000
TCGA-AX-A3FV	Endometrial	Non-Hispanic	White	0.000	0.000	0.000	1.000	0.000	0.000
TCGA-AX-A3FW	Endometrial	Non-Hispanic	White	0.000	0.000	0.038	0.962	0.000	0.000
TCGA-AX-A3FX	Endometrial	Non-Hispanic	White	0.383	0.000	0.014	0.482	0.121	0.000
TCGA-AX-A3FZ	Endometrial	Non-Hispanic	White	0.593	0.000	0.160	0.016	0.231	0.000
TCGA-AX-A3G1	Endometrial	Non-Hispanic	White	0.000	0.000	0.000	0.556	0.444	0.000
TCGA-AX-A3G8	Endometrial	Unknown	White	0.521	0.074	0.000	0.150	0.255	0.000
TCGA-AX-A3G9	Endometrial	Non-Hispanic	White	1.000	0.000	0.000	0.000	0.000	0.000
TCGA-AX-A3GB	Endometrial	Non-Hispanic	White	0.465	0.000	0.010	0.253	0.272	0.000
TCGA-B5-A0JR	Endometrial	Unknown	White	0.370	0.000	0.142	0.179	0.308	0.000
TCGA-B5-A0JS	Endometrial	Unknown	White	0.000	0.026	0.000	0.000	0.974	0.000
TCGA-B5-A0JT	Endometrial	Unknown	White	0.216	0.011	0.000	0.485	0.289	0.000
TCGA-B5-A0JV	Endometrial	Unknown	Black	0.000	0.020	0.000	0.980	0.000	0.000
TCGA-B5-A0JX	Endometrial	Unknown	White	0.000	0.000	0.000	1.000	0.000	0.000
TCGA-B5-A0JY	Endometrial	Unknown	White	1.000	0.000	0.000	0.000	0.000	0.000
TCGA-B5-A0JZ	Endometrial	Unknown	White	0.300	0.000	0.000	0.000	0.700	0.000
TCGA-B5-A0K0	Endometrial	Unknown	White	0.479	0.000	0.000	0.521	0.000	0.000
TCGA-B5-A0K1	Endometrial	Unknown	White	0.000	0.000	0.000	0.399	0.601	0.000
TCGA-B5-A0K2	Endometrial	Unknown	White	0.631	0.160	0.000	0.000	0.209	0.000
TCGA-B5-A0K3	Endometrial	Unknown	Black	0.386	0.102	0.000	0.123	0.389	0.000
TCGA-B5-A0K4	Endometrial	Unknown	Unknown	0.000	0.000	0.000	0.505	0.495	0.000
TCGA-B5-A0K6	Endometrial	Unknown	White	0.337	0.000	0.000	0.663	0.000	0.000
TCGA-B5-A0K7	Endometrial	Unknown	White	0.000	0.337	0.000	0.255	0.408	0.000
TCGA-B5-A0K9	Endometrial	Unknown	White	0.433	0.000	0.000	0.279	0.288	0.000
TCGA-B5-A11E	Endometrial	Unknown	White	0.000	0.294	0.000	0.479	0.227	0.000
TCGA-B5-A11F	Endometrial	Unknown	White	0.137	0.346	0.000	0.402	0.115	0.000
TCGA-B5-A11G	Endometrial	Unknown	Black	0.302	0.000	0.000	0.377	0.321	0.000

TCGA-B5-A11H	Endometrial	Unknown	White	0.534	0.000	0.000	0.000	0.450	0.016
TCGA-B5-A11I	Endometrial	Unknown	Black	0.288	0.349	0.000	0.000	0.329	0.034
TCGA-B5-A11J	Endometrial	Unknown	White	0.193	0.000	0.000	0.429	0.378	0.000
TCGA-B5-A11M	Endometrial	Unknown	White	0.200	0.000	0.093	0.000	0.706	0.000
TCGA-B5-A11N	Endometrial	Unknown	White	0.536	0.042	0.000	0.422	0.000	0.000
TCGA-B5-A11O	Endometrial	Unknown	White	0.904	0.000	0.000	0.096	0.000	0.000
TCGA-B5-A11Q	Endometrial	Unknown	White	0.390	0.000	0.000	0.000	0.610	0.000
TCGA-B5-A11R	Endometrial	Unknown	Black	1.000	0.000	0.000	0.000	0.000	0.000
TCGA-B5-A11S	Endometrial	Unknown	White	0.532	0.000	0.000	0.308	0.160	0.000
TCGA-B5-A11U	Endometrial	Unknown	Black	0.247	0.083	0.000	0.000	0.670	0.000
TCGA-B5-A11V	Endometrial	Unknown	Black	0.142	0.315	0.000	0.156	0.387	0.000
TCGA-B5-A11W	Endometrial	Unknown	White	0.372	0.019	0.000	0.167	0.431	0.012
TCGA-B5-A11X	Endometrial	Unknown	Black	0.683	0.029	0.000	0.000	0.260	0.028
TCGA-B5-A11Y	Endometrial	Unknown	White	0.106	0.429	0.011	0.454	0.000	0.000
TCGA-B5-A11Z	Endometrial	Unknown	White	0.273	0.000	0.040	0.000	0.686	0.000
TCGA-B5-A121	Endometrial	Unknown	White	0.000	0.000	0.000	1.000	0.000	0.000
TCGA-B5-A1MR	Endometrial	Unknown	White	0.470	0.000	0.000	0.284	0.212	0.034
TCGA-B5-A1MV	Endometrial	Unknown	White	0.000	0.000	0.000	0.000	1.000	0.000
TCGA-B5-A1MW	Endometrial	Unknown	White	0.000	0.000	0.137	0.862	0.000	0.000
TCGA-B5-A1MX	Endometrial	Unknown	White	0.062	0.000	0.000	0.677	0.260	0.000
TCGA-B5-A1MZ	Endometrial	Unknown	White	0.264	0.000	0.000	0.457	0.279	0.000
TCGA-B5-A3F9	Endometrial	Unknown	Black	0.513	0.000	0.056	0.289	0.141	0.000
TCGA-B5-A3FA	Endometrial	Unknown	White	0.470	0.000	0.000	0.000	0.530	0.000
TCGA-B5-A3FB	Endometrial	Unknown	Black	0.433	0.000	0.000	0.567	0.000	0.000
TCGA-B5-A3FC	Endometrial	Non-Hispanic	Black	0.493	0.000	0.017	0.173	0.317	0.000
TCGA-B5-A3FD	Endometrial	Unknown	Black	0.000	0.000	0.000	0.725	0.275	0.000
TCGA-B5-A3FH	Endometrial	Unknown	Black	0.531	0.018	0.000	0.451	0.000	0.000
TCGA-B5-A5OC	Endometrial	Unknown	Black	0.984	0.000	0.016	0.000	0.000	0.000
TCGA-BG-A0LW	Endometrial	Non-Hispanic	White	0.559	0.000	0.000	0.441	0.000	0.000
TCGA-BG-A0LX	Endometrial	Non-Hispanic	White	0.000	0.000	0.000	0.000	1.000	0.000
TCGA-BG-A0M0	Endometrial	Non-Hispanic	White	0.241	0.073	0.028	0.333	0.325	0.000
TCGA-BG-A0M2	Endometrial	Non-Hispanic	White	0.000	0.000	0.000	0.339	0.661	0.000
TCGA-BG-A0M3	Endometrial	Non-Hispanic	White	0.248	0.000	0.000	0.000	0.752	0.000
TCGA-BG-A0M4	Endometrial	Non-Hispanic	White	0.712	0.000	0.000	0.000	0.288	0.000
TCGA-BG-A0M7	Endometrial	Non-Hispanic	White	0.192	0.000	0.000	0.478	0.329	0.000
TCGA-BG-A0M8	Endometrial	Non-Hispanic	White	0.259	0.000	0.000	0.412	0.328	0.000
TCGA-BG-A0M9	Endometrial	Non-Hispanic	White	0.473	0.000	0.000	0.527	0.000	0.000
TCGA-BG-A0MA	Endometrial	Non-Hispanic	White	0.402	0.000	0.000	0.406	0.192	0.000
TCGA-BG-A0MC	Endometrial	Non-Hispanic	White	1.000	0.000	0.000	0.000	0.000	0.000
TCGA-BG-A0MG	Endometrial	Non-Hispanic	White	1.000	0.000	0.000	0.000	0.000	0.000
TCGA-BG-A0MH	Endometrial	Unknown	White	1.000	0.000	0.000	0.000	0.000	0.000
TCGA-BG-A0MI	Endometrial	Non-Hispanic	White	0.285	0.000	0.000	0.185	0.530	0.000

TCGA-BG-A0MK	Endometrial	Non-Hispanic	Black	0.381	0.000	0.000	0.619	0.000	0.000
TCGA-BG-A0MO	Endometrial	Non-Hispanic	White	1.000	0.000	0.000	0.000	0.000	0.000
TCGA-BG-A0MQ	Endometrial	Non-Hispanic	White	0.225	0.144	0.000	0.537	0.094	0.000
TCGA-BG-A0MS	Endometrial	Non-Hispanic	White	1.000	0.000	0.000	0.000	0.000	0.000
TCGA-BG-A0MT	Endometrial	Non-Hispanic	White	0.000	0.000	0.017	0.983	0.000	0.000
TCGA-BG-A0MU	Endometrial	Non-Hispanic	White	0.326	0.000	0.025	0.358	0.291	0.000
TCGA-BG-A0RY	Endometrial	Non-Hispanic	White	0.265	0.000	0.000	0.496	0.239	0.000
TCGA-BG-A0VT	Endometrial	Non-Hispanic	White	0.570	0.060	0.000	0.370	0.000	0.000
TCGA-BG-A0VV	Endometrial	Non-Hispanic	White	0.607	0.098	0.000	0.000	0.295	0.000
TCGA-BG-A0VW	Endometrial	Non-Hispanic	White	0.000	0.236	0.000	0.426	0.339	0.000
TCGA-BG-A0VX	Endometrial	Non-Hispanic	White	0.241	0.094	0.000	0.229	0.436	0.000
TCGA-BG-A0VZ	Endometrial	Non-Hispanic	White	0.000	0.000	0.000	1.000	0.000	0.000
TCGA-BG-A0W1	Endometrial	Non-Hispanic	White	0.000	0.205	0.000	0.795	0.000	0.000
TCGA-BG-A0W2	Endometrial	Unknown	White	0.368	0.007	0.000	0.410	0.215	0.000
TCGA-BG-A0YU	Endometrial	Non-Hispanic	White	0.268	0.185	0.000	0.547	0.000	0.000
TCGA-BG-A186	Endometrial	Non-Hispanic	Black	0.395	0.000	0.000	0.455	0.151	0.000
TCGA-BG-A187	Endometrial	Non-Hispanic	White	0.350	0.000	0.000	0.226	0.399	0.026
TCGA-BG-A18A	Endometrial	Non-Hispanic	White	0.358	0.000	0.000	0.420	0.222	0.000
TCGA-BG-A18B	Endometrial	Non-Hispanic	White	0.000	0.000	0.000	0.301	0.699	0.000
TCGA-BG-A18C	Endometrial	Non-Hispanic	White	0.885	0.000	0.000	0.000	0.000	0.115
TCGA-BG-A220	Endometrial	Non-Hispanic	White	0.337	0.052	0.000	0.315	0.296	0.000
TCGA-BG-A221	Endometrial	Non-Hispanic	White	0.391	0.109	0.000	0.288	0.213	0.000
TCGA-BG-A222	Endometrial	Non-Hispanic	White	0.000	0.000	0.000	0.000	1.000	0.000
TCGA-BG-A2AD	Endometrial	Non-Hispanic	White	0.000	0.000	0.000	1.000	0.000	0.000
TCGA-BG-A2AE	Endometrial	Non-Hispanic	White	0.000	0.000	0.000	1.000	0.000	0.000
TCGA-BG-A2L7	Endometrial	Non-Hispanic	White	0.676	0.000	0.000	0.324	0.000	0.000
TCGA-BG-A3EW	Endometrial	Non-Hispanic	White	0.064	0.000	0.252	0.000	0.685	0.000
TCGA-BK-A0C9	Endometrial	Non-Hispanic	White	0.510	0.000	0.000	0.000	0.490	0.000
TCGA-BK-A0CB	Endometrial	Non-Hispanic	White	0.275	0.000	0.000	0.558	0.166	0.000
TCGA-BK-A139	Endometrial	Non-Hispanic	White	0.158	0.000	0.000	0.191	0.651	0.000
TCGA-BK-A13B	Endometrial	Non-Hispanic	Black	0.549	0.000	0.059	0.000	0.392	0.000
TCGA-BK-A13C	Endometrial	Non-Hispanic	Black	0.199	0.000	0.000	0.559	0.242	0.000
TCGA-BK-A4ZD	Endometrial	Non-Hispanic	Black	0.559	0.000	0.022	0.329	0.000	0.089
TCGA-BK-A56F	Endometrial	Non-Hispanic	Black	0.654	0.000	0.308	0.037	0.000	0.000
TCGA-BK-A6W3	Endometrial	Non-Hispanic	Black	0.000	0.000	0.076	0.197	0.727	0.000
TCGA-BK-A6W4	Endometrial	Non-Hispanic	Black	0.434	0.139	0.000	0.000	0.402	0.026
TCGA-BS-A0T9	Endometrial	Non-Hispanic	Pacific	0.491	0.000	0.000	0.509	0.000	0.000
TCGA-BS-A0TA	Endometrial	Hispanic	Pacific	0.687	0.000	0.000	0.073	0.241	0.000
TCGA-BS-A0TC	Endometrial	Non-Hispanic	Asian	0.000	0.000	0.000	0.619	0.381	0.000
TCGA-BS-A0TD	Endometrial	Non-Hispanic	Asian	0.741	0.000	0.000	0.000	0.259	0.000
TCGA-BS-A0TE	Endometrial	Non-Hispanic	Asian	1.000	0.000	0.000	0.000	0.000	0.000
TCGA-BS-A0TG	Endometrial	Non-Hispanic	White	0.525	0.000	0.000	0.475	0.000	0.000

TCGA-BS-A0TI	Endometrial	Non-Hispanic	White	0.186	0.009	0.000	0.311	0.493	0.000
TCGA-BS-A0TJ	Endometrial	Non-Hispanic	Asian	0.000	0.000	0.000	0.000	1.000	0.000
TCGA-BS-A0U5	Endometrial	Non-Hispanic	White	0.000	0.000	0.050	0.950	0.000	0.000
TCGA-BS-A0U7	Endometrial	Non-Hispanic	White	0.310	0.020	0.000	0.000	0.670	0.000
TCGA-BS-A0U8	Endometrial	Non-Hispanic	White	0.197	0.000	0.000	0.662	0.140	0.000
TCGA-BS-A0UA	Endometrial	Non-Hispanic	White	0.334	0.000	0.000	0.666	0.000	0.000
TCGA-BS-A0UF	Endometrial	Non-Hispanic	Asian	0.467	0.000	0.000	0.169	0.364	0.000
TCGA-BS-A0UJ	Endometrial	Non-Hispanic	Asian	0.000	0.000	0.007	0.000	0.993	0.000
TCGA-BS-A0UL	Endometrial	Non-Hispanic	Pacific	0.228	0.000	0.000	0.000	0.772	0.000
TCGA-BS-A0UM	Endometrial	Non-Hispanic	White	0.000	0.000	0.000	0.597	0.403	0.000
TCGA-BS-A0UT	Endometrial	Non-Hispanic	Asian	0.526	0.000	0.000	0.213	0.261	0.000
TCGA-BS-A0UV	Endometrial	Non-Hispanic	Pacific	0.468	0.000	0.000	0.340	0.191	0.000
TCGA-BS-A0V4	Endometrial	Non-Hispanic	White	0.784	0.000	0.000	0.000	0.216	0.000
TCGA-BS-A0V6	Endometrial	Non-Hispanic	Asian	0.308	0.000	0.000	0.692	0.000	0.000
TCGA-BS-A0V7	Endometrial	Non-Hispanic	Pacific	0.000	0.028	0.000	0.000	0.972	0.000
TCGA-BS-A0V8	Endometrial	Non-Hispanic	Pacific	0.205	0.000	0.000	0.237	0.482	0.076
TCGA-BS-A0VI	Endometrial	Non-Hispanic	White	1.000	0.000	0.000	0.000	0.000	0.000
TCGA-BS-A0WQ	Endometrial	Non-Hispanic	Asian	0.709	0.000	0.057	0.000	0.234	0.000
TCGA-D1-A0ZN	Endometrial	Non-Hispanic	White	0.429	0.000	0.024	0.000	0.548	0.000
TCGA-D1-A0ZO	Endometrial	Non-Hispanic	White	0.130	0.000	0.000	0.428	0.385	0.057
TCGA-D1-A0ZQ	Endometrial	Non-Hispanic	White	0.000	0.000	0.000	0.000	1.000	0.000
TCGA-D1-A0ZR	Endometrial	Non-Hispanic	White	0.629	0.000	0.027	0.062	0.281	0.000
TCGA-D1-A0ZS	Endometrial	Non-Hispanic	White	0.706	0.000	0.032	0.262	0.000	0.000
TCGA-D1-A0ZU	Endometrial	Non-Hispanic	White	0.083	0.000	0.000	0.203	0.715	0.000
TCGA-D1-A0ZV	Endometrial	Non-Hispanic	White	0.133	0.030	0.000	0.620	0.217	0.000
TCGA-D1-A101	Endometrial	Non-Hispanic	White	0.092	0.350	0.000	0.000	0.558	0.000
TCGA-D1-A102	Endometrial	Non-Hispanic	White	0.137	0.000	0.023	0.328	0.512	0.000
TCGA-D1-A103	Endometrial	Non-Hispanic	White	0.376	0.000	0.086	0.102	0.436	0.000
TCGA-D1-A15W	Endometrial	Non-Hispanic	White	0.105	0.185	0.000	0.000	0.710	0.000
TCGA-D1-A15Z	Endometrial	Non-Hispanic	White	0.000	0.000	0.000	1.000	0.000	0.000
TCGA-D1-A160	Endometrial	Non-Hispanic	White	0.000	0.230	0.000	0.000	0.770	0.000
TCGA-D1-A161	Endometrial	Non-Hispanic	White	0.000	0.000	0.000	0.374	0.626	0.000
TCGA-D1-A162	Endometrial	Non-Hispanic	White	0.000	0.000	0.000	0.324	0.676	0.000
TCGA-D1-A163	Endometrial	Non-Hispanic	White	0.288	0.000	0.000	0.712	0.000	0.000
TCGA-D1-A165	Endometrial	Non-Hispanic	White	0.285	0.000	0.026	0.236	0.379	0.074
TCGA-D1-A167	Endometrial	Non-Hispanic	White	0.205	0.000	0.079	0.599	0.117	0.000
TCGA-D1-A168	Endometrial	Non-Hispanic	White	0.000	0.000	0.000	0.483	0.517	0.000
TCGA-D1-A169	Endometrial	Non-Hispanic	White	0.000	0.000	0.000	0.434	0.557	0.010
TCGA-D1-A16B	Endometrial	Non-Hispanic	White	0.579	0.000	0.000	0.245	0.176	0.000
TCGA-D1-A16D	Endometrial	Non-Hispanic	White	0.216	0.000	0.000	0.784	0.000	0.000
TCGA-D1-A16E	Endometrial	Non-Hispanic	White	0.639	0.000	0.000	0.361	0.000	0.000
TCGA-D1-A16F	Endometrial	Non-Hispanic	White	0.656	0.000	0.000	0.000	0.344	0.000

TCGA-D1-A16J	Endometrial	Non-Hispanic	White	0.000	0.000	0.000	0.690	0.310	0.000
TCGA-D1-A16N	Endometrial	Non-Hispanic	White	0.634	0.000	0.000	0.000	0.366	0.000
TCGA-D1-A16O	Endometrial	Non-Hispanic	White	0.118	0.000	0.000	0.554	0.328	0.000
TCGA-D1-A16Q	Endometrial	Non-Hispanic	White	0.000	0.000	0.000	0.625	0.375	0.000
TCGA-D1-A16R	Endometrial	Non-Hispanic	White	0.485	0.000	0.000	0.000	0.515	0.000
TCGA-D1-A16V	Endometrial	Non-Hispanic	White	0.128	0.000	0.000	0.345	0.526	0.000
TCGA-D1-A16X	Endometrial	Non-Hispanic	White	0.000	0.000	0.000	0.769	0.231	0.000
TCGA-D1-A16Y	Endometrial	Non-Hispanic	White	0.000	0.000	0.000	0.148	0.852	0.000
TCGA-D1-A174	Endometrial	Non-Hispanic	White	0.000	0.026	0.000	0.000	0.974	0.000
TCGA-D1-A175	Endometrial	Non-Hispanic	White	0.471	0.000	0.087	0.219	0.223	0.000
TCGA-D1-A176	Endometrial	Non-Hispanic	White	0.284	0.000	0.000	0.572	0.144	0.000
TCGA-D1-A177	Endometrial	Non-Hispanic	White	0.000	0.000	0.000	0.706	0.294	0.000
TCGA-D1-A17A	Endometrial	Non-Hispanic	White	0.781	0.000	0.000	0.000	0.219	0.000
TCGA-D1-A17B	Endometrial	Non-Hispanic	White	0.116	0.000	0.000	0.460	0.424	0.000
TCGA-D1-A17C	Endometrial	Non-Hispanic	White	0.207	0.000	0.011	0.199	0.583	0.000
TCGA-D1-A17D	Endometrial	Non-Hispanic	White	0.000	0.044	0.000	0.710	0.246	0.000
TCGA-D1-A17F	Endometrial	Non-Hispanic	White	0.521	0.000	0.000	0.328	0.151	0.000
TCGA-D1-A17H	Endometrial	Non-Hispanic	White	0.319	0.000	0.000	0.152	0.529	0.000
TCGA-D1-A17K	Endometrial	Non-Hispanic	White	0.554	0.000	0.000	0.000	0.419	0.027
TCGA-D1-A17L	Endometrial	Non-Hispanic	White	0.177	0.000	0.000	0.000	0.823	0.000
TCGA-D1-A17M	Endometrial	Non-Hispanic	White	0.686	0.173	0.000	0.141	0.000	0.000
TCGA-D1-A17N	Endometrial	Non-Hispanic	Unknown	0.361	0.000	0.000	0.265	0.374	0.000
TCGA-D1-A17Q	Endometrial	Non-Hispanic	White	0.241	0.025	0.000	0.421	0.312	0.000
TCGA-D1-A17R	Endometrial	Non-Hispanic	White	0.606	0.000	0.013	0.381	0.000	0.000
TCGA-D1-A17S	Endometrial	Non-Hispanic	White	0.105	0.086	0.000	0.235	0.574	0.000
TCGA-D1-A17T	Endometrial	Non-Hispanic	White	0.415	0.000	0.000	0.455	0.130	0.000
TCGA-D1-A17U	Endometrial	Non-Hispanic	White	1.000	0.000	0.000	0.000	0.000	0.000
TCGA-D1-A1NS	Endometrial	Non-Hispanic	White	0.484	0.078	0.000	0.240	0.198	0.000
TCGA-D1-A1NY	Endometrial	Non-Hispanic	White	0.000	0.000	0.000	0.000	1.000	0.000
TCGA-D1-A1NZ	Endometrial	Non-Hispanic	White	1.000	0.000	0.000	0.000	0.000	0.000
TCGA-D1-A1O0	Endometrial	Non-Hispanic	White	0.305	0.052	0.000	0.141	0.502	0.000
TCGA-D1-A1O5	Endometrial	Non-Hispanic	White	0.279	0.049	0.000	0.173	0.492	0.009
TCGA-D1-A1O7	Endometrial	Non-Hispanic	White	0.000	0.018	0.000	0.651	0.331	0.000
TCGA-D1-A1O8	Endometrial	Non-Hispanic	White	0.241	0.000	0.000	0.508	0.251	0.000
TCGA-D1-A2G5	Endometrial	Non-Hispanic	White	1.000	0.000	0.000	0.000	0.000	0.000
TCGA-D1-A2G6	Endometrial	Non-Hispanic	White	0.000	0.000	0.000	0.326	0.674	0.000
TCGA-D1-A3DA	Endometrial	Non-Hispanic	White	0.000	0.000	0.102	0.000	0.898	0.000
TCGA-D1-A3DG	Endometrial	Non-Hispanic	White	0.000	0.000	0.000	1.000	0.000	0.000
TCGA-D1-A3DH	Endometrial	Non-Hispanic	White	0.000	0.000	0.018	0.388	0.593	0.000
TCGA-DF-A2KN	Endometrial	Unknown	Unknown	0.296	0.000	0.037	0.000	0.667	0.000
TCGA-DF-A2KS	Endometrial	Unknown	Unknown	0.000	0.000	0.000	0.000	1.000	0.000
TCGA-DF-A2KU	Endometrial	Unknown	Unknown	0.377	0.000	0.000	0.158	0.465	0.000

TCGA-DF-A2KV	Endometrial	Unknown	Unknown	0.000	0.000	0.052	0.395	0.553	0.000
TCGA-DF-A2KY	Endometrial	Unknown	Unknown	0.000	0.000	0.000	0.000	1.000	0.000
TCGA-DF-A2KZ	Endometrial	Unknown	Unknown	0.602	0.000	0.000	0.220	0.177	0.000
TCGA-DF-A2L0	Endometrial	Unknown	Unknown	0.334	0.000	0.000	0.000	0.666	0.000
TCGA-DI-A0WH	Endometrial	Non-Hispanic	White	0.000	0.000	0.044	0.364	0.592	0.000
TCGA-DI-A1BY	Endometrial	Non-Hispanic	White	0.611	0.000	0.000	0.330	0.059	0.000
TCGA-DI-A1NO	Endometrial	Non-Hispanic	Black	0.934	0.000	0.000	0.000	0.000	0.066
TCGA-DI-A2QU	Endometrial	Non-Hispanic	White	0.373	0.000	0.000	0.302	0.279	0.046
TCGA-E6-A1LX	Endometrial	Non-Hispanic	White	0.329	0.144	0.000	0.526	0.000	0.000
TCGA-E6-A1M0	Endometrial	Non-Hispanic	White	0.434	0.015	0.000	0.104	0.448	0.000
TCGA-E6-A2P9	Endometrial	Non-Hispanic	White	1.000	0.000	0.000	0.000	0.000	0.000
TCGA-EC-A1NJ	Endometrial	Non-Hispanic	White	0.000	0.000	0.000	0.160	0.840	0.000
TCGA-EC-A1QX	Endometrial	Non-Hispanic	White	0.000	0.000	0.000	0.510	0.490	0.000
TCGA-EC-A24G	Endometrial	Non-Hispanic	White	0.000	0.000	0.000	0.462	0.538	0.000
TCGA-EO-A1Y7	Endometrial	Unknown	Unknown	0.757	0.000	0.000	0.000	0.243	0.000
TCGA-EO-A22R	Endometrial	Unknown	Unknown	0.000	0.000	0.000	0.753	0.247	0.000
TCGA-EO-A22S	Endometrial	Unknown	Unknown	0.566	0.000	0.000	0.000	0.434	0.000
TCGA-EO-A22T	Endometrial	Non-Hispanic	Asian	0.420	0.000	0.000	0.179	0.401	0.000
TCGA-EO-A22U	Endometrial	Unknown	White	0.705	0.000	0.000	0.236	0.000	0.059
TCGA-EO-A22X	Endometrial	Unknown	Unknown	0.000	0.000	0.038	0.320	0.640	0.002
TCGA-EO-A22Y	Endometrial	Non-Hispanic	White	0.615	0.000	0.224	0.000	0.161	0.000
TCGA-EO-A3AS	Endometrial	Unknown	Unknown	0.378	0.000	0.118	0.191	0.313	0.000
TCGA-EO-A3AU	Endometrial	Unknown	Unknown	0.000	0.000	0.000	0.690	0.310	0.000
TCGA-EO-A3AY	Endometrial	Unknown	Unknown	0.316	0.011	0.000	0.000	0.672	0.000
TCGA-EO-A3B0	Endometrial	Unknown	Asian	0.000	0.000	0.000	0.000	0.970	0.030
TCGA-EO-A3KX	Endometrial	Hispanic	White	0.208	0.000	0.120	0.268	0.405	0.000
TCGA-EO-A3L0	Endometrial	Non-Hispanic	White	0.000	0.000	0.091	0.909	0.000	0.000
TCGA-EY-A1G7	Endometrial	Non-Hispanic	White	0.379	0.000	0.000	0.416	0.205	0.000
TCGA-EY-A1G8	Endometrial	Non-Hispanic	White	0.403	0.000	0.000	0.417	0.180	0.000
TCGA-EY-A1GC	Endometrial	Non-Hispanic	White	0.341	0.042	0.000	0.499	0.118	0.000
TCGA-EY-A1GD	Endometrial	Non-Hispanic	Black	0.153	0.205	0.000	0.430	0.211	0.000
TCGA-EY-A1GE	Endometrial	Non-Hispanic	White	0.367	0.000	0.050	0.582	0.000	0.000
TCGA-EY-A1GF	Endometrial	Non-Hispanic	Black	0.000	0.000	0.035	0.965	0.000	0.000
TCGA-EY-A1GH	Endometrial	Non-Hispanic	White	1.000	0.000	0.000	0.000	0.000	0.000
TCGA-EY-A1GI	Endometrial	Non-Hispanic	White	0.280	0.000	0.000	0.369	0.351	0.000
TCGA-EY-A1GK	Endometrial	Non-Hispanic	White	0.855	0.145	0.000	0.000	0.000	0.000
TCGA-EY-A1GL	Endometrial	Unknown	White	0.395	0.000	0.031	0.000	0.554	0.020
TCGA-EY-A1GP	Endometrial	Non-Hispanic	Black	0.497	0.000	0.189	0.147	0.167	0.000
TCGA-EY-A1GQ	Endometrial	Non-Hispanic	White	0.260	0.000	0.000	0.223	0.517	0.000
TCGA-EY-A1GR	Endometrial	Non-Hispanic	Black	0.194	0.053	0.000	0.452	0.300	0.000
TCGA-EY-A1GT	Endometrial	Non-Hispanic	White	0.000	0.000	0.000	0.576	0.424	0.000
TCGA-EY-A1GU	Endometrial	Non-Hispanic	Black	1.000	0.000	0.000	0.000	0.000	0.000

TCGA-EY-A1GW	Endometrial	Hispanic	White	0.695	0.000	0.000	0.305	0.000	0.000
TCGA-EY-A1GX	Endometrial	Non-Hispanic	White	0.538	0.000	0.155	0.000	0.307	0.000
TCGA-EY-A1H0	Endometrial	Non-Hispanic	Black	0.311	0.000	0.000	0.689	0.000	0.000
TCGA-EY-A214	Endometrial	Unknown	White	0.000	0.000	0.000	0.000	1.000	0.000
TCGA-EY-A215	Endometrial	Non-Hispanic	White	0.000	0.000	0.025	0.975	0.000	0.000
TCGA-EY-A2OM	Endometrial	Non-Hispanic	White	0.434	0.000	0.000	0.430	0.136	0.000
TCGA-EY-A2OP	Endometrial	Non-Hispanic	White	0.000	0.000	0.113	0.468	0.419	0.000
TCGA-EY-A2OQ	Endometrial	Non-Hispanic	White	0.313	0.000	0.074	0.462	0.152	0.000
TCGA-EY-A548	Endometrial	Non-Hispanic	Black	0.235	0.000	0.172	0.000	0.592	0.000
TCGA-EY-A549	Endometrial	Non-Hispanic	Black	0.683	0.000	0.118	0.000	0.199	0.000
TCGA-EY-A54A	Endometrial	Non-Hispanic	Black	0.000	0.000	0.050	0.000	0.950	0.000
TCGA-EY-A5W2	Endometrial	Non-Hispanic	Black	0.273	0.000	0.000	0.528	0.199	0.000
TCGA-EY-A72D	Endometrial	Non-Hispanic	Black	0.000	0.000	0.000	0.565	0.435	0.000
TCGA-FI-A2CX	Endometrial	Non-Hispanic	White	0.964	0.036	0.000	0.000	0.000	0.000
TCGA-FI-A2D0	Endometrial	Non-Hispanic	White	0.200	0.116	0.028	0.427	0.229	0.000
TCGA-FI-A2D4	Endometrial	Unknown	White	0.000	0.254	0.000	0.608	0.139	0.000
TCGA-FI-A2D5	Endometrial	Non-Hispanic	White	0.000	0.000	0.000	0.737	0.263	0.000
TCGA-FI-A2D6	Endometrial	Non-Hispanic	White	0.800	0.000	0.000	0.000	0.200	0.000
TCGA-FI-A2F4	Endometrial	Non-Hispanic	Black	0.226	0.079	0.000	0.437	0.258	0.000
TCGA-FI-A2F9	Endometrial	Non-Hispanic	White	0.476	0.000	0.000	0.432	0.000	0.092
TCGA-H5-A2HR	Endometrial	Non-Hispanic	Black	0.642	0.000	0.000	0.000	0.358	0.000
TCGA-PG-A916	Endometrial	Non-Hispanic	Black	0.691	0.000	0.000	0.309	0.000	0.000
TCGA-PG-A917	Endometrial	Non-Hispanic	Black	0.336	0.000	0.000	0.108	0.557	0.000
TCGA-QF-A5YT	Endometrial	Non-Hispanic	Black	0.488	0.000	0.000	0.284	0.228	0.000
TCGA-QS-A5YQ	Endometrial	Non-Hispanic	Black	0.687	0.000	0.000	0.213	0.100	0.000
TCGA-QS-A744	Endometrial	Non-Hispanic	Black	0.325	0.000	0.112	0.487	0.000	0.076
TCGA-SJ-A6ZI	Endometrial	Non-Hispanic	Black	0.340	0.000	0.062	0.221	0.377	0.000
TCGA-SJ-A6ZJ	Endometrial	Non-Hispanic	Black	0.000	0.000	0.032	0.325	0.643	0.000
TCGA-SL-A6J9	Endometrial	Non-Hispanic	Black	0.540	0.000	0.000	0.386	0.000	0.074
TCGA-SL-A6JA	Endometrial	Non-Hispanic	Black	0.237	0.004	0.060	0.000	0.698	0.000
TCGA-04-1331	Ovary	Non-Hispanic	White	0.000	0.736	0.000	0.000	0.000	0.264
TCGA-04-1332	Ovary	Non-Hispanic	White	0.000	0.553	0.000	0.253	0.000	0.194
TCGA-04-1338	Ovary	Non-Hispanic	White	0.378	0.000	0.000	0.000	0.497	0.125
TCGA-04-1341	Ovary	Non-Hispanic	White	0.603	0.167	0.000	0.000	0.000	0.230
TCGA-04-1343	Ovary	Non-Hispanic	White	0.000	0.126	0.038	0.000	0.320	0.515
TCGA-04-1347	Ovary	Non-Hispanic	White	0.106	0.101	0.000	0.145	0.357	0.291
TCGA-04-1350	Ovary	Unknown	White	0.000	0.319	0.018	0.203	0.459	0.000
TCGA-04-1356	Ovary	Hispanic	White	0.541	0.050	0.383	0.000	0.000	0.026
TCGA-04-1357	Ovary	Unknown	Unknown	0.226	0.755	0.018	0.000	0.000	0.000
TCGA-04-1361	Ovary	Unknown	White	0.000	0.321	0.079	0.000	0.125	0.476
TCGA-04-1362	Ovary	Unknown	White	0.000	0.000	0.583	0.000	0.417	0.000
TCGA-04-1364	Ovary	Non-Hispanic	White	0.000	0.000	0.000	0.136	0.473	0.392

TCGA-04-1365	Ovary	Non-Hispanic	White	0.167	0.097	0.088	0.000	0.208	0.440
TCGA-04-1514	Ovary	Non-Hispanic	White	0.000	0.000	0.758	0.071	0.170	0.000
TCGA-04-1519	Ovary	Unknown	Unknown	0.000	0.184	0.337	0.000	0.479	0.000
TCGA-04-1530	Ovary	Non-Hispanic	White	0.003	0.406	0.287	0.000	0.304	0.000
TCGA-04-1536	Ovary	Unknown	Black	0.497	0.276	0.095	0.066	0.067	0.000
TCGA-04-1648	Ovary	Unknown	White	0.000	0.000	0.510	0.272	0.218	0.000
TCGA-04-1651	Ovary	Non-Hispanic	White	0.000	0.000	0.458	0.542	0.000	0.000
TCGA-04-1655	Ovary	Unknown	White	0.158	0.000	0.245	0.388	0.210	0.000
TCGA-09-0364	Ovary	Non-Hispanic	White	0.185	0.000	0.431	0.080	0.286	0.018
TCGA-09-0366	Ovary	Unknown	White	0.000	0.000	0.358	0.000	0.117	0.525
TCGA-09-0367	Ovary	Non-Hispanic	Pacific	0.000	0.002	0.000	0.447	0.480	0.072
TCGA-09-0369	Ovary	Non-Hispanic	White	0.000	0.446	0.000	0.185	0.369	0.000
TCGA-09-1659	Ovary	Non-Hispanic	White	0.499	0.000	0.000	0.000	0.000	0.501
TCGA-09-1661	Ovary	Non-Hispanic	White	0.311	0.157	0.037	0.000	0.495	0.000
TCGA-09-1662	Ovary	Non-Hispanic	White	0.335	0.000	0.665	0.000	0.000	0.000
TCGA-09-1665	Ovary	Non-Hispanic	White	0.000	0.000	0.000	0.000	0.000	1.000
TCGA-09-1666	Ovary	Non-Hispanic	White	0.565	0.000	0.355	0.080	0.000	0.000
TCGA-09-1667	Ovary	Non-Hispanic	White	0.307	0.016	0.065	0.612	0.000	0.000
TCGA-09-1668	Ovary	Non-Hispanic	White	0.000	0.231	0.112	0.000	0.472	0.185
TCGA-09-1669	Ovary	Non-Hispanic	White	0.000	0.361	0.291	0.232	0.000	0.116
TCGA-09-1670	Ovary	Unknown	White	0.000	0.369	0.066	0.172	0.334	0.060
TCGA-09-1673	Ovary	Non-Hispanic	White	0.000	0.238	0.205	0.000	0.558	0.000
TCGA-09-2045	Ovary	Non-Hispanic	Asian	0.303	0.000	0.259	0.296	0.143	0.000
TCGA-09-2048	Ovary	Non-Hispanic	White	0.000	0.324	0.000	0.144	0.000	0.532
TCGA-09-2053	Ovary	Non-Hispanic	White	0.105	0.287	0.173	0.344	0.000	0.091
TCGA-09-2054	Ovary	Non-Hispanic	Black	0.621	0.061	0.000	0.000	0.072	0.245
TCGA-09-2056	Ovary	Hispanic	White	0.168	0.391	0.082	0.000	0.360	0.000
TCGA-10-0927	Ovary	Hispanic	Unknown	0.374	0.048	0.000	0.000	0.578	0.000
TCGA-10-0928	Ovary	Unknown	White	0.651	0.137	0.190	0.022	0.000	0.000
TCGA-10-0931	Ovary	Unknown	White	0.000	0.437	0.187	0.297	0.000	0.078
TCGA-10-0933	Ovary	Non-Hispanic	White	0.000	1.000	0.000	0.000	0.000	0.000
TCGA-10-0936	Ovary	Non-Hispanic	Black	0.427	0.000	0.000	0.000	0.000	0.573
TCGA-10-0937	Ovary	Unknown	White	0.000	0.093	0.039	0.331	0.537	0.000
TCGA-10-0938	Ovary	Unknown	White	0.430	0.138	0.311	0.000	0.057	0.064
TCGA-13-0714	Ovary	Non-Hispanic	White	0.000	0.224	0.000	0.511	0.265	0.000
TCGA-13-0720	Ovary	Non-Hispanic	White	0.040	0.236	0.151	0.317	0.256	0.000
TCGA-13-0724	Ovary	Hispanic	White	0.406	0.287	0.000	0.000	0.000	0.307
TCGA-13-0725	Ovary	Hispanic	White	0.000	0.283	0.112	0.605	0.000	0.000
TCGA-13-0726	Ovary	Non-Hispanic	White	0.000	0.488	0.078	0.000	0.434	0.000
TCGA-13-0727	Ovary	Non-Hispanic	White	0.187	0.479	0.126	0.000	0.208	0.000
TCGA-13-0730	Ovary	Non-Hispanic	White	0.473	0.201	0.000	0.326	0.000	0.000
TCGA-13-0762	Ovary	Non-Hispanic	White	0.233	0.246	0.355	0.039	0.000	0.127

TCGA-13-0765	Ovary	Non-Hispanic	White	0.000	0.217	0.084	0.000	0.410	0.288
TCGA-13-0766	Ovary	Non-Hispanic	White	0.672	0.328	0.000	0.000	0.000	0.000
TCGA-13-0768	Ovary	Non-Hispanic	White	0.000	0.311	0.245	0.144	0.177	0.124
TCGA-13-0795	Ovary	Non-Hispanic	White	0.310	0.118	0.000	0.000	0.339	0.233
TCGA-13-0797	Ovary	Non-Hispanic	White	0.219	0.276	0.000	0.199	0.306	0.000
TCGA-13-0800	Ovary	Non-Hispanic	White	0.030	0.754	0.000	0.000	0.000	0.215
TCGA-13-0804	Ovary	Non-Hispanic	White	0.237	0.304	0.000	0.218	0.000	0.242
TCGA-13-0883	Ovary	Non-Hispanic	White	0.000	0.105	0.333	0.193	0.368	0.000
TCGA-13-0884	Ovary	Non-Hispanic	White	0.519	0.000	0.481	0.000	0.000	0.000
TCGA-13-0885	Ovary	Non-Hispanic	White	0.000	0.448	0.000	0.212	0.314	0.026
TCGA-13-0886	Ovary	Non-Hispanic	White	0.000	0.474	0.224	0.302	0.000	0.000
TCGA-13-0887	Ovary	Non-Hispanic	White	0.060	0.451	0.000	0.104	0.385	0.000
TCGA-13-0888	Ovary	Non-Hispanic	White	0.000	0.509	0.000	0.295	0.072	0.124
TCGA-13-0893	Ovary	Non-Hispanic	Black	0.000	0.979	0.021	0.000	0.000	0.000
TCGA-13-0897	Ovary	Non-Hispanic	White	0.125	0.327	0.007	0.306	0.235	0.000
TCGA-13-0900	Ovary	Non-Hispanic	White	0.093	0.323	0.000	0.000	0.342	0.242
TCGA-13-0901	Ovary	Non-Hispanic	Asian	0.668	0.000	0.012	0.000	0.000	0.320
TCGA-13-0905	Ovary	Non-Hispanic	White	0.000	0.000	0.681	0.000	0.319	0.000
TCGA-13-0906	Ovary	Non-Hispanic	White	0.354	0.285	0.151	0.057	0.000	0.153
TCGA-13-0908	Ovary	Non-Hispanic	White	0.000	0.000	0.962	0.037	0.000	0.000
TCGA-13-0911	Ovary	Non-Hispanic	White	0.535	0.182	0.011	0.202	0.000	0.070
TCGA-13-0913	Ovary	Non-Hispanic	White	0.715	0.000	0.000	0.029	0.180	0.076
TCGA-13-0916	Ovary	Non-Hispanic	White	0.000	0.470	0.285	0.245	0.000	0.000
TCGA-13-0920	Ovary	Non-Hispanic	White	0.000	0.183	0.035	0.215	0.294	0.273
TCGA-13-0923	Ovary	Non-Hispanic	White	0.339	0.407	0.000	0.254	0.000	0.000
TCGA-13-0924	Ovary	Non-Hispanic	White	0.441	0.000	0.120	0.395	0.043	0.000
TCGA-13-1403	Ovary	Non-Hispanic	White	0.000	0.311	0.131	0.437	0.000	0.121
TCGA-13-1404	Ovary	Non-Hispanic	White	0.000	0.024	0.322	0.582	0.000	0.072
TCGA-13-1405	Ovary	Non-Hispanic	White	0.000	0.306	0.106	0.506	0.081	0.000
TCGA-13-1407	Ovary	Non-Hispanic	White	0.000	0.000	0.000	0.012	0.000	0.988
TCGA-13-1408	Ovary	Non-Hispanic	White	0.000	0.000	0.447	0.000	0.000	0.553
TCGA-13-1409	Ovary	Non-Hispanic	White	0.000	0.265	0.474	0.260	0.000	0.000
TCGA-13-1410	Ovary	Non-Hispanic	White	0.000	0.560	0.029	0.000	0.410	0.000
TCGA-13-1411	Ovary	Non-Hispanic	White	0.000	0.000	0.435	0.000	0.336	0.230
TCGA-13-1477	Ovary	Non-Hispanic	White	0.000	0.438	0.406	0.000	0.157	0.000
TCGA-13-1483	Ovary	Non-Hispanic	White	0.000	0.501	0.064	0.180	0.000	0.254
TCGA-13-1485	Ovary	Non-Hispanic	White	0.000	0.000	0.398	0.602	0.000	0.000
TCGA-13-1487	Ovary	Non-Hispanic	White	0.318	0.000	0.000	0.316	0.000	0.366
TCGA-13-1488	Ovary	Non-Hispanic	White	0.183	0.133	0.044	0.142	0.292	0.206
TCGA-13-1489	Ovary	Non-Hispanic	White	0.000	0.000	0.234	0.518	0.000	0.248
TCGA-13-1492	Ovary	Non-Hispanic	White	0.355	0.358	0.174	0.000	0.000	0.114
TCGA-13-1495	Ovary	Non-Hispanic	White	0.000	0.373	0.317	0.000	0.294	0.015

TCGA-13-1496	Ovary	Non-Hispanic	White	0.190	0.004	0.097	0.105	0.290	0.312
TCGA-13-1497	Ovary	Non-Hispanic	White	0.472	0.265	0.000	0.109	0.000	0.153
TCGA-13-1498	Ovary	Non-Hispanic	White	0.290	0.000	0.432	0.000	0.000	0.279
TCGA-13-1499	Ovary	Non-Hispanic	White	0.308	0.269	0.194	0.000	0.000	0.229
TCGA-13-1501	Ovary	Non-Hispanic	White	0.000	0.000	0.000	0.685	0.000	0.315
TCGA-13-1505	Ovary	Non-Hispanic	White	0.000	0.202	0.000	0.492	0.000	0.305
TCGA-13-1506	Ovary	Non-Hispanic	White	0.167	0.132	0.134	0.000	0.297	0.270
TCGA-13-1509	Ovary	Non-Hispanic	White	0.000	0.120	0.385	0.000	0.495	0.000
TCGA-13-1510	Ovary	Non-Hispanic	White	0.276	0.168	0.222	0.335	0.000	0.000
TCGA-13-1511	Ovary	Non-Hispanic	Asian	0.000	0.601	0.000	0.252	0.147	0.000
TCGA-13-1512	Ovary	Non-Hispanic	White	0.096	0.135	0.029	0.285	0.279	0.175
TCGA-13-2060	Ovary	Unknown	White	0.402	0.243	0.057	0.000	0.088	0.210
TCGA-13-A5FT	Ovary	Unknown	Black	0.000	0.000	0.000	0.000	0.377	0.623
TCGA-20-0987	Ovary	Non-Hispanic	White	0.509	0.000	0.000	0.355	0.000	0.136
TCGA-20-0991	Ovary	Non-Hispanic	White	0.339	0.253	0.000	0.000	0.329	0.079
TCGA-20-1682	Ovary	Non-Hispanic	White	0.020	0.308	0.012	0.660	0.000	0.000
TCGA-20-1683	Ovary	Non-Hispanic	White	0.000	0.202	0.401	0.000	0.397	0.000
TCGA-20-1686	Ovary	Non-Hispanic	White	0.000	0.335	0.000	0.310	0.239	0.117
TCGA-20-1687	Ovary	Non-Hispanic	White	0.000	0.224	0.000	0.508	0.217	0.051
TCGA-23-1021	Ovary	Non-Hispanic	White	0.747	0.041	0.000	0.212	0.000	0.000
TCGA-23-1022	Ovary	Non-Hispanic	White	0.386	0.614	0.000	0.000	0.000	0.000
TCGA-23-1023	Ovary	Non-Hispanic	White	0.309	0.361	0.091	0.000	0.132	0.107
TCGA-23-1024	Ovary	Non-Hispanic	White	0.000	0.160	0.399	0.000	0.130	0.311
TCGA-23-1026	Ovary	Non-Hispanic	White	0.000	0.568	0.182	0.000	0.250	0.000
TCGA-23-1027	Ovary	Non-Hispanic	White	0.000	0.265	0.290	0.006	0.439	0.000
TCGA-23-1028	Ovary	Hispanic	White	0.331	0.307	0.046	0.207	0.109	0.000
TCGA-23-1029	Ovary	Non-Hispanic	White	0.403	0.565	0.031	0.000	0.000	0.000
TCGA-23-1030	Ovary	Non-Hispanic	White	0.291	0.147	0.043	0.076	0.269	0.174
TCGA-23-1107	Ovary	Non-Hispanic	White	0.614	0.000	0.000	0.254	0.081	0.052
TCGA-23-1109	Ovary	Non-Hispanic	White	0.255	0.068	0.352	0.000	0.255	0.069
TCGA-23-1110	Ovary	Hispanic	White	0.888	0.000	0.014	0.000	0.000	0.098
TCGA-23-1111	Ovary	Unknown	White	0.000	0.346	0.239	0.078	0.267	0.070
TCGA-23-1113	Ovary	Non-Hispanic	White	0.114	0.290	0.000	0.275	0.321	0.000
TCGA-23-1114	Ovary	Non-Hispanic	White	0.000	0.000	0.000	0.000	0.000	1.000
TCGA-23-1116	Ovary	Non-Hispanic	White	0.242	0.126	0.000	0.242	0.389	0.000
TCGA-23-1118	Ovary	Non-Hispanic	White	0.000	0.000	0.331	0.206	0.010	0.453
TCGA-23-1119	Ovary	Non-Hispanic	White	0.136	0.047	0.000	0.317	0.318	0.183
TCGA-23-1120	Ovary	Non-Hispanic	White	0.000	0.110	0.511	0.218	0.000	0.161
TCGA-23-1123	Ovary	Non-Hispanic	White	0.000	0.664	0.056	0.279	0.000	0.000
TCGA-23-1809	Ovary	Non-Hispanic	White	0.000	0.000	0.000	0.000	0.000	1.000
TCGA-23-2077	Ovary	Non-Hispanic	White	0.000	0.183	0.000	0.000	0.425	0.392
TCGA-23-2078	Ovary	Non-Hispanic	White	0.211	0.192	0.378	0.079	0.000	0.140

TCGA-23-2084	Ovary	Non-Hispanic	White	0.000	0.472	0.119	0.000	0.380	0.029
TCGA-24-0966	Ovary	Non-Hispanic	Black	0.535	0.399	0.010	0.000	0.000	0.057
TCGA-24-0968	Ovary	Unknown	White	0.000	0.400	0.000	0.000	0.359	0.241
TCGA-24-0970	Ovary	Unknown	White	0.000	0.507	0.000	0.475	0.000	0.018
TCGA-24-0979	Ovary	Unknown	White	0.000	0.734	0.000	0.228	0.000	0.037
TCGA-24-0982	Ovary	Unknown	White	0.041	0.531	0.163	0.226	0.038	0.000
TCGA-24-1103	Ovary	Unknown	Black	0.236	0.152	0.000	0.000	0.481	0.130
TCGA-24-1104	Ovary	Unknown	White	0.227	0.280	0.131	0.077	0.084	0.202
TCGA-24-1105	Ovary	Unknown	White	0.000	0.000	0.041	0.000	0.000	0.959
TCGA-24-1413	Ovary	Unknown	White	0.170	0.000	0.232	0.000	0.430	0.169
TCGA-24-1416	Ovary	Unknown	White	0.222	0.000	0.000	0.245	0.375	0.159
TCGA-24-1417	Ovary	Unknown	White	0.000	0.362	0.181	0.457	0.000	0.000
TCGA-24-1418	Ovary	Unknown	White	0.000	0.349	0.411	0.000	0.240	0.000
TCGA-24-1419	Ovary	Unknown	White	0.000	1.000	0.000	0.000	0.000	0.000
TCGA-24-1422	Ovary	Unknown	Black	0.075	0.504	0.187	0.057	0.000	0.177
TCGA-24-1423	Ovary	Unknown	White	0.033	0.470	0.000	0.243	0.000	0.255
TCGA-24-1425	Ovary	Unknown	White	0.202	0.184	0.340	0.000	0.000	0.274
TCGA-24-1426	Ovary	Unknown	White	0.000	0.000	1.000	0.000	0.000	0.000
TCGA-24-1427	Ovary	Unknown	Unknown	0.000	0.000	0.631	0.369	0.000	0.000
TCGA-24-1428	Ovary	Unknown	White	0.000	0.173	0.369	0.040	0.000	0.418
TCGA-24-1430	Ovary	Unknown	White	0.000	0.000	0.880	0.120	0.000	0.000
TCGA-24-1431	Ovary	Unknown	White	0.941	0.059	0.000	0.000	0.000	0.000
TCGA-24-1434	Ovary	Unknown	White	0.178	0.482	0.000	0.000	0.000	0.340
TCGA-24-1464	Ovary	Unknown	White	0.241	0.759	0.000	0.000	0.000	0.000
TCGA-24-1467	Ovary	Unknown	White	0.000	0.000	0.032	0.770	0.000	0.198
TCGA-24-1469	Ovary	Unknown	White	0.000	0.081	0.311	0.000	0.179	0.429
TCGA-24-1470	Ovary	Unknown	White	0.000	0.000	0.888	0.000	0.000	0.112
TCGA-24-1471	Ovary	Unknown	White	0.000	0.631	0.000	0.146	0.223	0.000
TCGA-24-1474	Ovary	Unknown	Black	0.209	0.000	0.130	0.156	0.221	0.285
TCGA-24-1544	Ovary	Unknown	Black	0.429	0.278	0.000	0.000	0.276	0.016
TCGA-24-1546	Ovary	Unknown	White	0.000	0.756	0.244	0.000	0.000	0.000
TCGA-24-1550	Ovary	Unknown	White	0.000	0.596	0.000	0.000	0.000	0.404
TCGA-24-1551	Ovary	Unknown	White	0.021	0.190	0.126	0.285	0.378	0.000
TCGA-24-1552	Ovary	Unknown	White	0.206	0.161	0.065	0.345	0.178	0.046
TCGA-24-1553	Ovary	Unknown	White	0.000	0.212	0.139	0.000	0.321	0.328
TCGA-24-1557	Ovary	Unknown	White	0.051	0.114	0.062	0.364	0.409	0.000
TCGA-24-1560	Ovary	Unknown	White	0.000	0.492	0.392	0.116	0.000	0.000
TCGA-24-1562	Ovary	Unknown	White	0.632	0.368	0.000	0.000	0.000	0.000
TCGA-24-1563	Ovary	Unknown	Black	0.000	0.486	0.000	0.000	0.378	0.136
TCGA-24-1565	Ovary	Unknown	White	0.009	0.000	0.272	0.135	0.000	0.584
TCGA-24-1567	Ovary	Unknown	White	0.000	0.683	0.000	0.000	0.317	0.000
TCGA-24-1603	Ovary	Unknown	White	0.000	0.329	0.119	0.354	0.000	0.198

TCGA-24-1604	Ovary	Unknown	White	0.000	0.000	0.669	0.331	0.000	0.000
TCGA-24-1616	Ovary	Unknown	White	0.000	0.151	0.000	0.673	0.176	0.000
TCGA-24-1842	Ovary	Unknown	White	0.673	0.002	0.325	0.000	0.000	0.000
TCGA-24-1843	Ovary	Unknown	White	0.000	0.075	0.000	0.000	0.185	0.740
TCGA-24-1844	Ovary	Unknown	White	0.000	0.279	0.142	0.234	0.000	0.345
TCGA-24-1846	Ovary	Unknown	Unknown	0.318	0.441	0.233	0.000	0.009	0.000
TCGA-24-1847	Ovary	Unknown	White	0.113	0.438	0.135	0.315	0.000	0.000
TCGA-24-1850	Ovary	Unknown	White	0.000	0.179	0.000	0.241	0.404	0.177
TCGA-24-1923	Ovary	Unknown	White	0.128	0.360	0.136	0.000	0.118	0.259
TCGA-24-1924	Ovary	Unknown	White	0.000	0.532	0.073	0.395	0.000	0.000
TCGA-24-1928	Ovary	Unknown	White	0.405	0.289	0.253	0.000	0.000	0.053
TCGA-24-1930	Ovary	Unknown	White	0.000	0.296	0.000	0.012	0.511	0.180
TCGA-24-2020	Ovary	Unknown	White	0.175	0.693	0.000	0.000	0.132	0.000
TCGA-24-2023	Ovary	Unknown	White	0.171	0.187	0.052	0.266	0.000	0.323
TCGA-24-2024	Ovary	Unknown	Black	0.000	0.000	0.493	0.000	0.000	0.507
TCGA-24-2026	Ovary	Unknown	White	0.000	0.000	0.000	0.000	0.000	1.000
TCGA-24-2027	Ovary	Unknown	White	0.000	0.196	0.559	0.245	0.000	0.000
TCGA-24-2033	Ovary	Unknown	White	0.121	0.467	0.092	0.305	0.000	0.015
TCGA-24-2035	Ovary	Unknown	White	0.000	0.215	0.000	0.324	0.238	0.223
TCGA-24-2036	Ovary	Unknown	White	0.000	0.000	1.000	0.000	0.000	0.000
TCGA-24-2038	Ovary	Unknown	White	0.000	0.231	0.443	0.058	0.268	0.000
TCGA-24-2254	Ovary	Unknown	White	0.356	0.370	0.274	0.000	0.000	0.000
TCGA-24-2267	Ovary	Unknown	White	0.000	0.470	0.173	0.227	0.129	0.000
TCGA-24-2271	Ovary	Unknown	Asian	0.555	0.165	0.050	0.178	0.052	0.000
TCGA-24-2280	Ovary	Unknown	White	0.000	0.000	0.000	0.000	0.000	1.000
TCGA-24-2288	Ovary	Unknown	White	0.342	0.280	0.000	0.197	0.000	0.181
TCGA-24-2289	Ovary	Unknown	White	0.000	0.000	0.099	0.287	0.000	0.614
TCGA-24-2290	Ovary	Unknown	White	0.000	0.331	0.135	0.533	0.000	0.000
TCGA-24-2293	Ovary	Unknown	White	0.000	0.000	0.107	0.000	0.000	0.893
TCGA-24-2297	Ovary	Unknown	White	0.000	0.274	0.390	0.007	0.000	0.330
TCGA-24-2298	Ovary	Unknown	White	0.180	0.020	0.590	0.000	0.209	0.000
TCGA-25-1312	Ovary	Non-Hispanic	White	0.247	0.196	0.107	0.354	0.000	0.095
TCGA-25-1313	Ovary	Non-Hispanic	White	0.259	0.114	0.119	0.262	0.247	0.000
TCGA-25-1315	Ovary	Non-Hispanic	White	0.000	0.012	0.503	0.000	0.248	0.237
TCGA-25-1316	Ovary	Non-Hispanic	White	0.000	0.388	0.225	0.130	0.257	0.000
TCGA-25-1318	Ovary	Non-Hispanic	White	0.000	0.425	0.089	0.000	0.485	0.000
TCGA-25-1319	Ovary	Non-Hispanic	White	0.000	0.637	0.000	0.363	0.000	0.000
TCGA-25-1320	Ovary	Non-Hispanic	White	0.000	0.308	0.340	0.000	0.000	0.352
TCGA-25-1321	Ovary	Non-Hispanic	White	0.119	0.000	0.261	0.139	0.055	0.425
TCGA-25-1322	Ovary	Non-Hispanic	White	0.000	1.000	0.000	0.000	0.000	0.000
TCGA-25-1323	Ovary	Non-Hispanic	White	0.000	0.000	0.237	0.000	0.000	0.763
TCGA-25-1326	Ovary	Non-Hispanic	White	0.000	0.139	0.214	0.000	0.535	0.112

TCGA-25-1328	Ovary	Non-Hispanic	White	0.000	0.144	0.466	0.000	0.390	0.000
TCGA-25-1329	Ovary	Non-Hispanic	White	0.000	0.000	0.383	0.199	0.000	0.418
TCGA-25-1623	Ovary	Non-Hispanic	White	0.000	0.119	0.191	0.000	0.678	0.013
TCGA-25-1626	Ovary	Non-Hispanic	White	0.000	0.380	0.394	0.017	0.000	0.209
TCGA-25-1627	Ovary	Non-Hispanic	White	0.000	0.482	0.000	0.518	0.000	0.000
TCGA-25-1628	Ovary	Non-Hispanic	White	0.000	0.000	0.643	0.000	0.357	0.000
TCGA-25-1630	Ovary	Non-Hispanic	White	0.265	0.298	0.095	0.178	0.164	0.000
TCGA-25-1631	Ovary	Non-Hispanic	White	0.108	0.279	0.194	0.000	0.285	0.134
TCGA-25-1633	Ovary	Non-Hispanic	White	0.000	0.000	0.103	0.641	0.000	0.256
TCGA-25-1634	Ovary	Non-Hispanic	White	0.000	0.617	0.000	0.383	0.000	0.000
TCGA-25-1635	Ovary	Non-Hispanic	White	0.000	0.301	0.243	0.231	0.062	0.163
TCGA-25-1870	Ovary	Non-Hispanic	White	0.450	0.000	0.550	0.000	0.000	0.000
TCGA-25-1877	Ovary	Non-Hispanic	White	0.000	0.319	0.032	0.362	0.000	0.287
TCGA-25-2042	Ovary	Non-Hispanic	American Indian	0.000	1.000	0.000	0.000	0.000	0.000
TCGA-25-2391	Ovary	Non-Hispanic	White	0.000	0.300	0.106	0.321	0.000	0.273
TCGA-25-2392	Ovary	Non-Hispanic	White	0.316	0.588	0.044	0.052	0.000	0.000
TCGA-25-2393	Ovary	Non-Hispanic	White	0.496	0.130	0.000	0.000	0.086	0.287
TCGA-25-2396	Ovary	Non-Hispanic	White	0.000	0.478	0.034	0.455	0.000	0.032
TCGA-25-2398	Ovary	Non-Hispanic	White	0.036	0.326	0.166	0.000	0.471	0.000
TCGA-25-2399	Ovary	Non-Hispanic	White	0.000	0.299	0.275	0.000	0.185	0.241
TCGA-25-2400	Ovary	Non-Hispanic	White	0.000	0.361	0.000	0.639	0.000	0.000
TCGA-25-2401	Ovary	Non-Hispanic	White	0.612	0.388	0.000	0.000	0.000	0.000
TCGA-25-2404	Ovary	Non-Hispanic	American Indian	0.266	0.585	0.149	0.000	0.000	0.000
TCGA-29-1688	Ovary	Unknown	White	0.639	0.000	0.305	0.000	0.055	0.000
TCGA-29-1690	Ovary	Unknown	White	0.023	0.326	0.092	0.560	0.000	0.000
TCGA-29-1691	Ovary	Unknown	White	0.000	0.524	0.000	0.325	0.000	0.151
TCGA-29-1693	Ovary	Unknown	White	0.380	0.598	0.023	0.000	0.000	0.000
TCGA-29-1694	Ovary	Unknown	White	0.000	0.490	0.450	0.000	0.000	0.060
TCGA-29-1695	Ovary	Unknown	White	0.000	0.882	0.118	0.000	0.000	0.000
TCGA-29-1696	Ovary	Unknown	White	0.386	0.481	0.086	0.000	0.047	0.000
TCGA-29-1697	Ovary	Unknown	White	0.000	0.430	0.309	0.261	0.000	0.000
TCGA-29-1701	Ovary	Unknown	White	0.000	0.570	0.000	0.000	0.430	0.000
TCGA-29-1703	Ovary	Unknown	Black	0.000	0.563	0.000	0.000	0.437	0.000
TCGA-29-1705	Ovary	Unknown	White	0.026	0.448	0.078	0.431	0.000	0.017
TCGA-29-1707	Ovary	Unknown	White	0.183	0.321	0.067	0.000	0.286	0.143
TCGA-29-1710	Ovary	Unknown	White	0.083	0.333	0.041	0.319	0.000	0.225
TCGA-29-1711	Ovary	Unknown	Black	0.264	0.179	0.000	0.373	0.000	0.185
TCGA-29-1761	Ovary	Unknown	Asian	0.000	0.461	0.000	0.000	0.539	0.000
TCGA-29-1762	Ovary	Unknown	White	0.000	0.447	0.286	0.262	0.005	0.000
TCGA-29-1763	Ovary	Unknown	Black	0.000	0.000	0.520	0.000	0.281	0.198

TCGA-29-1766	Ovary	Unknown	White	0.453	0.229	0.000	0.052	0.023	0.242
TCGA-29-1768	Ovary	Unknown	White	0.000	0.268	0.073	0.658	0.000	0.000
TCGA-29-1769	Ovary	Unknown	White	0.000	0.369	0.000	0.000	0.631	0.000
TCGA-29-1770	Ovary	Unknown	White	0.065	0.543	0.000	0.258	0.134	0.000
TCGA-29-1774	Ovary	Non-Hispanic	White	0.089	0.495	0.000	0.075	0.316	0.026
TCGA-29-1776	Ovary	Unknown	Black	0.000	0.386	0.000	0.107	0.507	0.000
TCGA-29-1777	Ovary	Unknown	White	0.000	0.433	0.000	0.000	0.567	0.000
TCGA-29-1778	Ovary	Unknown	White	0.148	0.561	0.229	0.000	0.061	0.000
TCGA-29-1781	Ovary	Unknown	White	0.063	0.463	0.054	0.420	0.000	0.000
TCGA-29-1783	Ovary	Unknown	White	0.000	0.174	0.047	0.393	0.276	0.110
TCGA-29-1784	Ovary	Unknown	White	0.000	0.634	0.000	0.000	0.252	0.115
TCGA-29-1785	Ovary	Unknown	White	0.000	0.269	0.000	0.526	0.129	0.076
TCGA-29-2414	Ovary	Unknown	White	0.000	0.000	0.249	0.000	0.000	0.751
TCGA-29-2425	Ovary	Unknown	White	0.071	0.518	0.000	0.411	0.000	0.000
TCGA-29-2428	Ovary	Unknown	White	0.269	0.210	0.000	0.189	0.034	0.297
TCGA-29-A5NZ	Ovary	Unknown	Black	0.000	0.000	0.392	0.136	0.000	0.472
TCGA-30-1714	Ovary	Non-Hispanic	White	0.730	0.000	0.000	0.000	0.000	0.270
TCGA-30-1718	Ovary	Non-Hispanic	White	0.498	0.285	0.000	0.059	0.104	0.053
TCGA-30-1853	Ovary	Non-Hispanic	White	0.000	0.336	0.045	0.567	0.052	0.000
TCGA-30-1857	Ovary	Non-Hispanic	White	0.000	0.295	0.063	0.400	0.180	0.061
TCGA-30-1860	Ovary	Hispanic	White	0.168	0.295	0.114	0.349	0.075	0.000
TCGA-30-1861	Ovary	Non-Hispanic	White	0.000	0.229	0.056	0.335	0.085	0.295
TCGA-30-1862	Ovary	Non-Hispanic	White	0.525	0.164	0.283	0.000	0.000	0.028
TCGA-30-1866	Ovary	Non-Hispanic	White	0.000	0.211	0.090	0.000	0.386	0.312
TCGA-30-1891	Ovary	Non-Hispanic	White	0.000	0.398	0.000	0.000	0.602	0.000
TCGA-30-1892	Ovary	Non-Hispanic	White	0.000	0.390	0.000	0.610	0.000	0.000
TCGA-31-1944	Ovary	Non-Hispanic	White	0.272	0.271	0.000	0.222	0.235	0.000
TCGA-31-1946	Ovary	Unknown	Unknown	0.392	0.608	0.000	0.000	0.000	0.000
TCGA-31-1950	Ovary	Non-Hispanic	White	0.334	0.000	0.000	0.000	0.000	0.666
TCGA-31-1951	Ovary	Non-Hispanic	White	0.000	0.162	0.370	0.219	0.248	0.000
TCGA-31-1953	Ovary	Non-Hispanic	Asian	0.000	0.547	0.151	0.302	0.000	0.000
TCGA-31-1956	Ovary	Non-Hispanic	White	0.000	0.419	0.004	0.577	0.000	0.000
TCGA-31-1959	Ovary	Non-Hispanic	White	0.000	0.373	0.031	0.000	0.596	0.000
TCGA-36-1568	Ovary	Unknown	Unknown	0.171	0.000	0.085	0.000	0.406	0.338
TCGA-36-1569	Ovary	Unknown	White	0.000	0.411	0.029	0.370	0.000	0.190
TCGA-36-1570	Ovary	Unknown	White	0.222	0.106	0.518	0.000	0.000	0.154
TCGA-36-1571	Ovary	Unknown	White	0.029	0.399	0.107	0.465	0.000	0.000
TCGA-36-1574	Ovary	Unknown	Asian	0.192	0.052	0.457	0.000	0.299	0.000
TCGA-36-1576	Ovary	Unknown	Unknown	0.000	0.300	0.000	0.568	0.000	0.132
TCGA-36-1577	Ovary	Unknown	Asian	0.444	0.054	0.502	0.000	0.000	0.000
TCGA-36-1580	Ovary	Unknown	Unknown	0.000	0.538	0.149	0.312	0.000	0.000
TCGA-36-1581	Ovary	Unknown	White	0.000	0.000	0.000	0.000	0.000	1.000

TCGA-57-1582	Ovary	Unknown	White	0.556	0.244	0.200	0.000	0.000	0.000
TCGA-57-1583	Ovary	Unknown	Black	0.434	0.090	0.000	0.000	0.203	0.272
TCGA-57-1584	Ovary	Non-Hispanic	White	0.491	0.232	0.113	0.000	0.164	0.000
TCGA-57-1585	Ovary	Non-Hispanic	White	0.000	0.532	0.113	0.000	0.000	0.355
TCGA-57-1586	Ovary	Non-Hispanic	White	0.577	0.000	0.000	0.000	0.000	0.423
TCGA-57-1994	Ovary	Unknown	White	0.122	0.384	0.048	0.000	0.000	0.445
TCGA-59-2350	Ovary	Unknown	Unknown	0.394	0.000	0.606	0.000	0.000	0.000
TCGA-59-2351	Ovary	Non-Hispanic	White	0.218	0.000	0.093	0.000	0.268	0.421
TCGA-59-2354	Ovary	Non-Hispanic	White	0.000	0.962	0.038	0.000	0.000	0.000
TCGA-59-2355	Ovary	Non-Hispanic	White	0.000	0.456	0.143	0.359	0.000	0.042
TCGA-59-2363	Ovary	Non-Hispanic	Asian	0.038	0.364	0.233	0.107	0.237	0.020
TCGA-59-A5PD	Ovary	Non-Hispanic	Black	0.275	0.214	0.171	0.095	0.186	0.059
TCGA-5X-AA5U	Ovary	Unknown	Black	0.000	0.000	0.000	0.000	0.000	1.000
TCGA-61-1721	Ovary	Non-Hispanic	White	0.492	0.472	0.000	0.036	0.000	0.000
TCGA-61-1724	Ovary	Non-Hispanic	White	0.000	0.331	0.195	0.000	0.365	0.108
TCGA-61-1725	Ovary	Non-Hispanic	White	0.205	0.212	0.084	0.421	0.078	0.000
TCGA-61-1728	Ovary	Non-Hispanic	White	0.182	0.598	0.000	0.000	0.000	0.220
TCGA-61-1736	Ovary	Non-Hispanic	White	0.395	0.263	0.216	0.115	0.010	0.000
TCGA-61-1737	Ovary	Non-Hispanic	White	0.259	0.341	0.269	0.132	0.000	0.000
TCGA-61-1738	Ovary	Unknown	Black	0.000	0.000	0.000	0.613	0.000	0.386
TCGA-61-1741	Ovary	Non-Hispanic	White	0.265	0.187	0.518	0.000	0.000	0.029
TCGA-61-1900	Ovary	Non-Hispanic	Black	0.000	0.448	0.275	0.000	0.000	0.277
TCGA-61-1907	Ovary	Non-Hispanic	White	0.053	0.175	0.131	0.375	0.266	0.000
TCGA-61-1910	Ovary	Non-Hispanic	White	0.384	0.300	0.096	0.117	0.013	0.089
TCGA-61-1911	Ovary	Non-Hispanic	White	0.374	0.000	0.000	0.375	0.208	0.043
TCGA-61-1914	Ovary	Unknown	Unknown	0.000	0.425	0.015	0.231	0.329	0.000
TCGA-61-1918	Ovary	Non-Hispanic	White	0.000	0.640	0.000	0.360	0.000	0.000
TCGA-61-1919	Ovary	Non-Hispanic	White	0.000	0.532	0.000	0.000	0.393	0.075
TCGA-61-1995	Ovary	Non-Hispanic	White	0.000	0.473	0.233	0.294	0.000	0.000
TCGA-61-1998	Ovary	Non-Hispanic	White	0.154	0.554	0.067	0.225	0.000	0.000
TCGA-61-2000	Ovary	Non-Hispanic	White	0.457	0.513	0.000	0.000	0.000	0.030
TCGA-61-2002	Ovary	Non-Hispanic	White	0.121	0.406	0.051	0.199	0.000	0.223
TCGA-61-2003	Ovary	Non-Hispanic	White	0.336	0.331	0.212	0.121	0.000	0.000
TCGA-61-2008	Ovary	Non-Hispanic	Asian	0.000	0.540	0.324	0.000	0.136	0.000
TCGA-61-2009	Ovary	Non-Hispanic	White	0.000	1.000	0.000	0.000	0.000	0.000
TCGA-61-2012	Ovary	Non-Hispanic	White	0.000	0.000	0.000	0.150	0.135	0.715
TCGA-61-2088	Ovary	Non-Hispanic	White	0.000	0.578	0.000	0.422	0.000	0.000
TCGA-61-2092	Ovary	Unknown	White	0.000	0.656	0.000	0.000	0.344	0.000
TCGA-61-2097	Ovary	Unknown	White	0.000	0.000	0.178	0.212	0.610	0.000
TCGA-61-2098	Ovary	Unknown	White	0.000	0.000	0.000	0.000	0.000	1.000
TCGA-61-2101	Ovary	Non-Hispanic	White	0.000	0.771	0.000	0.000	0.000	0.229
TCGA-61-2102	Ovary	Unknown	White	0.418	0.000	0.000	0.399	0.000	0.183

TCGA-61-2104	Ovary	Unknown	White	0.000	0.746	0.000	0.000	0.008	0.246
TCGA-61-2113	Ovary	Non-Hispanic	White	0.281	0.342	0.000	0.000	0.000	0.376
TCGA-OY-A56Q	Ovary	Non-Hispanic	Black	0.000	0.000	0.243	0.000	0.563	0.195
TCGA-VG-A8LO	Ovary	Non-Hispanic	Black	0.008	0.000	0.317	0.314	0.075	0.286
TCGA-WR-A838	Ovary	Non-Hispanic	Black	0.000	0.000	0.649	0.000	0.351	0.000

Table 3. Q-matrix with 3 cluster solution at the subpopulations structure analysis of endometrial cancer TCGA patients. For each patient there is also information about tumor type and self-declared race and ethnicity.

ID	Tumor type	Ethnicity	Race	Cluster 1	Cluster 2	Cluster 3
TCGA-2E-A9G8	Endometrial	Non-Hispanic	Black	0.000	0.678	0.322
TCGA-4E-A92E	Endometrial	Non-Hispanic	Black	0.116	0.884	0.000
TCGA-5B-A90C	Endometrial	Non-Hispanic	Black	0.000	1.000	0.000
TCGA-A5-A0G9	Endometrial	Unknown	White	0.248	0.000	0.752
TCGA-A5-A0GA	Endometrial	Non-Hispanic	White	0.883	0.000	0.117
TCGA-A5-A0GB	Endometrial	Unknown	White	0.630	0.000	0.370
TCGA-A5-A0GD	Endometrial	Unknown	White	0.731	0.000	0.269
TCGA-A5-A0GE	Endometrial	Non-Hispanic	Asian	0.096	0.147	0.757
TCGA-A5-A0GG	Endometrial	Non-Hispanic	Black	0.388	0.138	0.475
TCGA-A5-A0GH	Endometrial	Non-Hispanic	White	0.660	0.084	0.256
TCGA-A5-A0GI	Endometrial	Non-Hispanic	White	0.274	0.000	0.726
TCGA-A5-A0GJ	Endometrial	Non-Hispanic	White	0.000	0.000	1.000
TCGA-A5-A0GM	Endometrial	Non-Hispanic	White	0.339	0.000	0.661
TCGA-A5-A0GN	Endometrial	Non-Hispanic	White	0.000	0.000	1.000
TCGA-A5-A0GP	Endometrial	Non-Hispanic	Asian	0.304	0.000	0.696
TCGA-A5-A0GQ	Endometrial	Non-Hispanic	White	0.614	0.000	0.386
TCGA-A5-A0GR	Endometrial	Non-Hispanic	Black	0.000	0.000	1.000
TCGA-A5-A0GU	Endometrial	Unknown	White	0.959	0.000	0.041
TCGA-A5-A0GV	Endometrial	Non-Hispanic	White	0.317	0.000	0.683
TCGA-A5-A0GW	Endometrial	Non-Hispanic	Asian	0.415	0.067	0.518
TCGA-A5-A0GX	Endometrial	Unknown	White	0.000	0.000	1.000
TCGA-A5-A0R7	Endometrial	Non-Hispanic	White	0.273	0.056	0.671
TCGA-A5-A0R8	Endometrial	Non-Hispanic	White	0.288	0.000	0.712
TCGA-A5-A0R9	Endometrial	Non-Hispanic	White	0.367	0.041	0.592
TCGA-A5-A0RA	Endometrial	Non-Hispanic	White	0.000	0.000	1.000
TCGA-A5-A0VO	Endometrial	Non-Hispanic	White	0.846	0.000	0.154
TCGA-A5-A0VP	Endometrial	Non-Hispanic	White	0.942	0.000	0.058
TCGA-A5-A0VQ	Endometrial	Hispanic	Unknown	0.418	0.107	0.475
TCGA-A5-A1OJ	Endometrial	Non-Hispanic	Asian	0.309	0.085	0.606
TCGA-A5-A1OK	Endometrial	Non-Hispanic	White	1.000	0.000	0.000
TCGA-A5-A2K5	Endometrial	Non-Hispanic	White	0.000	0.373	0.627
TCGA-A5-A2K7	Endometrial	Hispanic	Unknown	0.000	0.771	0.229

TCGA-A5-A3LO	Endometrial	Non-Hispanic	White	0.000	0.800	0.200
TCGA-A5-A7WJ	Endometrial	Non-Hispanic	Black	0.000	1.000	0.000
TCGA-A5-AB3J	Endometrial	Non-Hispanic	Black	0.000	1.000	0.000
TCGA-AJ-A23O	Endometrial	Non-Hispanic	White	0.736	0.000	0.264
TCGA-AJ-A2QK	Endometrial	Hispanic	White	0.568	0.000	0.432
TCGA-AJ-A2QL	Endometrial	Non-Hispanic	White	0.721	0.000	0.279
TCGA-AJ-A2QN	Endometrial	Unknown	White	0.724	0.000	0.276
TCGA-AJ-A2QO	Endometrial	Unknown	White	0.218	0.751	0.032
TCGA-AJ-A3BH	Endometrial	Non-Hispanic	White	0.221	0.736	0.043
TCGA-AJ-A3BI	Endometrial	Non-Hispanic	White	0.000	0.000	1.000
TCGA-AJ-A3BK	Endometrial	Non-Hispanic	White	0.000	0.603	0.397
TCGA-AJ-A3EK	Endometrial	Hispanic	White	0.075	0.925	0.000
TCGA-AJ-A3EL	Endometrial	Unknown	Pacific	0.000	0.594	0.406
TCGA-AJ-A3EM	Endometrial	Unknown	White	0.036	0.832	0.132
TCGA-AJ-A3I9	Endometrial	Non-Hispanic	White	0.000	0.923	0.077
TCGA-AJ-A3NC	Endometrial	Unknown	White	0.090	0.910	0.000
TCGA-AJ-A3NE	Endometrial	Non-Hispanic	White	0.178	0.822	0.000
TCGA-AJ-A3OJ	Endometrial	Non-Hispanic	White	0.299	0.672	0.029
TCGA-AJ-A3OL	Endometrial	Non-Hispanic	White	0.064	0.936	0.000
TCGA-AJ-A5DV	Endometrial	Non-Hispanic	Black	0.000	0.956	0.044
TCGA-AJ-A8CT	Endometrial	Unknown	Black	0.000	0.907	0.093
TCGA-AJ-A8CV	Endometrial	Unknown	Black	0.040	0.905	0.055
TCGA-AJ-A8CW	Endometrial	Non-Hispanic	Black	0.073	0.927	0.000
TCGA-AP-A051	Endometrial	Unknown	White	0.918	0.014	0.067
TCGA-AP-A053	Endometrial	Non-Hispanic	White	0.465	0.000	0.535
TCGA-AP-A054	Endometrial	Non-Hispanic	White	0.765	0.207	0.028
TCGA-AP-A056	Endometrial	Unknown	White	0.558	0.000	0.442
TCGA-AP-A059	Endometrial	Non-Hispanic	White	0.965	0.000	0.035
TCGA-AP-A05N	Endometrial	Unknown	White	0.242	0.000	0.758
TCGA-AP-A05O	Endometrial	Unknown	White	0.883	0.000	0.117
TCGA-AP-A05P	Endometrial	Non-Hispanic	Asian	0.797	0.172	0.032
TCGA-AP-A0LD	Endometrial	Unknown	White	0.138	0.041	0.822
TCGA-AP-A0LE	Endometrial	Unknown	White	0.000	0.000	1.000
TCGA-AP-A0LF	Endometrial	Non-Hispanic	White	0.000	0.000	1.000
TCGA-AP-A0LG	Endometrial	Unknown	White	0.795	0.026	0.179
TCGA-AP-A0LJ	Endometrial	Unknown	White	0.000	0.000	1.000
TCGA-AP-A0LL	Endometrial	Unknown	White	0.000	0.000	1.000
TCGA-AP-A0LM	Endometrial	Unknown	Black	0.107	0.145	0.748
TCGA-AP-A0LN	Endometrial	Unknown	Black	0.134	0.000	0.866
TCGA-AP-A0LO	Endometrial	Non-Hispanic	White	0.302	0.000	0.698
TCGA-AP-A0LP	Endometrial	Unknown	White	0.411	0.000	0.589
TCGA-AP-A0LS	Endometrial	Unknown	White	0.649	0.000	0.351

TCGA-AP-A0LT	Endometrial	Unknown	White	0.656	0.000	0.344
TCGA-AP-A0LV	Endometrial	Unknown	White	0.408	0.147	0.445
TCGA-AP-A1DH	Endometrial	Unknown	White	0.247	0.074	0.678
TCGA-AP-A1DK	Endometrial	Unknown	White	0.150	0.000	0.850
TCGA-AP-A1DM	Endometrial	Unknown	White	0.502	0.039	0.459
TCGA-AP-A1DO	Endometrial	Non-Hispanic	White	0.345	0.060	0.596
TCGA-AP-A1DP	Endometrial	Unknown	White	0.314	0.000	0.686
TCGA-AP-A1DR	Endometrial	Unknown	Black	0.252	0.113	0.635
TCGA-AP-A1DV	Endometrial	Non-Hispanic	White	0.973	0.000	0.027
TCGA-AP-A1E0	Endometrial	Unknown	White	0.956	0.000	0.044
TCGA-AP-A1E1	Endometrial	Unknown	White	0.793	0.000	0.207
TCGA-AP-A1E3	Endometrial	Unknown	White	0.403	0.000	0.597
TCGA-AP-A1E4	Endometrial	Unknown	White	0.607	0.083	0.310
TCGA-AW-A1PO	Endometrial	Non-Hispanic	White	0.620	0.121	0.259
TCGA-AX-A05S	Endometrial	Non-Hispanic	White	0.046	0.000	0.954
TCGA-AX-A05T	Endometrial	Unknown	White	0.085	0.000	0.915
TCGA-AX-A05U	Endometrial	Non-Hispanic	American Indian	0.476	0.000	0.523
TCGA-AX-A05W	Endometrial	Non-Hispanic	White	0.000	0.167	0.833
TCGA-AX-A05Y	Endometrial	Non-Hispanic	White	0.250	0.000	0.750
TCGA-AX-A05Z	Endometrial	Hispanic	Unknown	0.000	0.000	1.000
TCGA-AX-A060	Endometrial	Non-Hispanic	American Indian	0.106	0.000	0.894
TCGA-AX-A062	Endometrial	Unknown	White	0.000	0.000	1.000
TCGA-AX-A063	Endometrial	Non-Hispanic	White	0.066	0.000	0.934
TCGA-AX-A064	Endometrial	Unknown	White	0.000	0.000	1.000
TCGA-AX-A06B	Endometrial	Non-Hispanic	White	0.494	0.090	0.416
TCGA-AX-A06D	Endometrial	Unknown	White	0.165	0.135	0.699
TCGA-AX-A06F	Endometrial	Non-Hispanic	White	0.316	0.000	0.684
TCGA-AX-A06H	Endometrial	Non-Hispanic	American Indian	0.233	0.028	0.739
TCGA-AX-A06J	Endometrial	Non-Hispanic	White	0.050	0.000	0.950
TCGA-AX-A06L	Endometrial	Unknown	White	0.000	0.000	1.000
TCGA-AX-A0IS	Endometrial	Non-Hispanic	White	0.526	0.000	0.474
TCGA-AX-A0IZ	Endometrial	Non-Hispanic	White	0.514	0.071	0.415
TCGA-AX-A0J0	Endometrial	Non-Hispanic	White	1.000	0.000	0.000
TCGA-AX-A0J1	Endometrial	Non-Hispanic	White	0.974	0.000	0.026
TCGA-AX-A1C4	Endometrial	Non-Hispanic	White	0.915	0.000	0.085
TCGA-AX-A1C5	Endometrial	Non-Hispanic	White	0.804	0.000	0.196
TCGA-AX-A1C9	Endometrial	Non-Hispanic	White	0.647	0.013	0.340
TCGA-AX-A1CE	Endometrial	Non-Hispanic	White	0.455	0.000	0.545
TCGA-AX-A1CF	Endometrial	Non-Hispanic	White	0.480	0.015	0.505

TCGA-AX-A1CI	Endometrial	Non-Hispanic	White	0.277	0.015	0.708
TCGA-AX-A1CJ	Endometrial	Non-Hispanic	Black	0.000	0.000	1.000
TCGA-AX-A1CK	Endometrial	Non-Hispanic	White	0.251	0.141	0.608
TCGA-AX-A1CN	Endometrial	Non-Hispanic	White	1.000	0.000	0.000
TCGA-AX-A2H7	Endometrial	Non-Hispanic	White	0.453	0.000	0.547
TCGA-AX-A2H8	Endometrial	Non-Hispanic	White	0.529	0.025	0.447
TCGA-AX-A2HA	Endometrial	Unknown	White	0.434	0.000	0.566
TCGA-AX-A2HC	Endometrial	Non-Hispanic	Black	0.583	0.276	0.142
TCGA-AX-A2HD	Endometrial	Non-Hispanic	White	0.906	0.022	0.072
TCGA-AX-A2HG	Endometrial	Hispanic	White	0.508	0.088	0.403
TCGA-AX-A2HH	Endometrial	Non-Hispanic	White	0.152	0.848	0.000
TCGA-AX-A2HJ	Endometrial	Non-Hispanic	Asian	0.508	0.181	0.311
TCGA-AX-A2HK	Endometrial	Unknown	White	0.623	0.115	0.262
TCGA-AX-A2IN	Endometrial	Unknown	White	0.000	0.787	0.213
TCGA-AX-A3FS	Endometrial	Non-Hispanic	White	0.093	0.812	0.095
TCGA-AX-A3FT	Endometrial	Non-Hispanic	White	0.000	1.000	0.000
TCGA-AX-A3FV	Endometrial	Non-Hispanic	White	0.000	0.461	0.539
TCGA-AX-A3FW	Endometrial	Non-Hispanic	White	0.000	0.621	0.379
TCGA-AX-A3FX	Endometrial	Non-Hispanic	White	0.000	0.539	0.461
TCGA-AX-A3FZ	Endometrial	Non-Hispanic	White	0.000	0.810	0.190
TCGA-AX-A3G1	Endometrial	Non-Hispanic	White	0.000	0.545	0.455
TCGA-AX-A3G8	Endometrial	Unknown	White	0.000	0.320	0.680
TCGA-AX-A3G9	Endometrial	Non-Hispanic	White	0.000	0.728	0.272
TCGA-AX-A3GB	Endometrial	Non-Hispanic	White	0.000	0.679	0.321
TCGA-B5-A0JR	Endometrial	Unknown	White	0.144	0.832	0.024
TCGA-B5-A0JS	Endometrial	Unknown	White	0.645	0.000	0.355
TCGA-B5-A0JT	Endometrial	Unknown	White	1.000	0.000	0.000
TCGA-B5-A0JV	Endometrial	Unknown	Black	0.349	0.094	0.557
TCGA-B5-A0JX	Endometrial	Unknown	White	0.831	0.000	0.169
TCGA-B5-A0JY	Endometrial	Unknown	White	0.554	0.000	0.446
TCGA-B5-A0JZ	Endometrial	Unknown	White	0.574	0.011	0.415
TCGA-B5-A0K0	Endometrial	Unknown	White	0.803	0.122	0.075
TCGA-B5-A0K1	Endometrial	Unknown	White	0.602	0.000	0.398
TCGA-B5-A0K2	Endometrial	Unknown	White	0.108	0.000	0.892
TCGA-B5-A0K3	Endometrial	Unknown	Black	0.272	0.000	0.728
TCGA-B5-A0K4	Endometrial	Unknown	Unknown	0.000	0.000	1.000
TCGA-B5-A0K6	Endometrial	Unknown	White	0.000	0.000	1.000
TCGA-B5-A0K7	Endometrial	Unknown	White	0.408	0.000	0.592
TCGA-B5-A0K9	Endometrial	Unknown	White	0.102	0.898	0.000
TCGA-B5-A11E	Endometrial	Unknown	White	0.656	0.000	0.344
TCGA-B5-A11F	Endometrial	Unknown	White	0.609	0.000	0.391
TCGA-B5-A11G	Endometrial	Unknown	Black	0.000	0.086	0.914

TCGA-B5-A11H	Endometrial	Unknown	White	0.705	0.000	0.295
TCGA-B5-A11I	Endometrial	Unknown	Black	0.408	0.173	0.419
TCGA-B5-A11J	Endometrial	Unknown	White	0.873	0.000	0.127
TCGA-B5-A11M	Endometrial	Unknown	White	0.915	0.085	0.000
TCGA-B5-A11N	Endometrial	Unknown	White	0.584	0.000	0.416
TCGA-B5-A11O	Endometrial	Unknown	White	0.812	0.016	0.172
TCGA-B5-A11Q	Endometrial	Unknown	White	0.124	0.036	0.840
TCGA-B5-A11R	Endometrial	Unknown	Black	0.007	0.872	0.121
TCGA-B5-A11S	Endometrial	Unknown	White	0.883	0.046	0.071
TCGA-B5-A11U	Endometrial	Unknown	Black	0.427	0.193	0.380
TCGA-B5-A11V	Endometrial	Unknown	Black	0.511	0.085	0.404
TCGA-B5-A11W	Endometrial	Unknown	White	0.514	0.000	0.486
TCGA-B5-A11X	Endometrial	Unknown	Black	0.384	0.142	0.474
TCGA-B5-A11Y	Endometrial	Unknown	White	0.590	0.000	0.410
TCGA-B5-A11Z	Endometrial	Unknown	White	0.974	0.000	0.026
TCGA-B5-A121	Endometrial	Unknown	White	0.963	0.000	0.037
TCGA-B5-A1MR	Endometrial	Unknown	White	0.093	0.000	0.907
TCGA-B5-A1MV	Endometrial	Unknown	White	0.965	0.000	0.035
TCGA-B5-A1MW	Endometrial	Unknown	White	0.084	0.916	0.000
TCGA-B5-A1MX	Endometrial	Unknown	White	0.929	0.010	0.061
TCGA-B5-A1MZ	Endometrial	Unknown	White	0.758	0.000	0.242
TCGA-B5-A3F9	Endometrial	Unknown	Black	0.000	0.960	0.040
TCGA-B5-A3FA	Endometrial	Unknown	White	0.284	0.706	0.011
TCGA-B5-A3FB	Endometrial	Unknown	Black	0.105	0.847	0.048
TCGA-B5-A3FC	Endometrial	Non-Hispanic	Black	0.000	0.725	0.275
TCGA-B5-A3FD	Endometrial	Unknown	Black	0.000	0.573	0.427
TCGA-B5-A3FH	Endometrial	Unknown	Black	0.000	0.814	0.186
TCGA-B5-A5OC	Endometrial	Unknown	Black	0.000	1.000	0.000
TCGA-BG-A0LW	Endometrial	Non-Hispanic	White	0.746	0.065	0.189
TCGA-BG-A0LX	Endometrial	Non-Hispanic	White	0.754	0.000	0.246
TCGA-BG-A0M0	Endometrial	Non-Hispanic	White	0.419	0.089	0.492
TCGA-BG-A0M2	Endometrial	Non-Hispanic	White	0.313	0.000	0.687
TCGA-BG-A0M3	Endometrial	Non-Hispanic	White	0.671	0.000	0.329
TCGA-BG-A0M4	Endometrial	Non-Hispanic	White	0.335	0.000	0.665
TCGA-BG-A0M7	Endometrial	Non-Hispanic	White	0.226	0.000	0.774
TCGA-BG-A0M8	Endometrial	Non-Hispanic	White	0.201	0.000	0.799
TCGA-BG-A0M9	Endometrial	Non-Hispanic	White	0.131	0.000	0.869
TCGA-BG-A0MA	Endometrial	Non-Hispanic	White	0.933	0.000	0.067
TCGA-BG-A0MC	Endometrial	Non-Hispanic	White	0.000	0.000	1.000
TCGA-BG-A0MG	Endometrial	Non-Hispanic	White	0.414	0.000	0.586
TCGA-BG-A0MH	Endometrial	Unknown	White	0.857	0.000	0.143
TCGA-BG-A0MI	Endometrial	Non-Hispanic	White	0.119	0.000	0.881

TCGA-BG-A0MK	Endometrial	Non-Hispanic	Black	0.000	0.615	0.385
TCGA-BG-A0MO	Endometrial	Non-Hispanic	White	0.351	0.000	0.649
TCGA-BG-A0MQ	Endometrial	Non-Hispanic	White	0.585	0.000	0.415
TCGA-BG-A0MS	Endometrial	Non-Hispanic	White	0.794	0.000	0.206
TCGA-BG-A0MT	Endometrial	Non-Hispanic	White	0.732	0.000	0.268
TCGA-BG-A0MU	Endometrial	Non-Hispanic	White	0.367	0.000	0.633
TCGA-BG-A0RY	Endometrial	Non-Hispanic	White	0.530	0.000	0.470
TCGA-BG-A0VT	Endometrial	Non-Hispanic	White	0.594	0.000	0.406
TCGA-BG-A0VV	Endometrial	Non-Hispanic	White	0.615	0.000	0.385
TCGA-BG-A0VW	Endometrial	Non-Hispanic	White	0.000	0.000	1.000
TCGA-BG-A0VX	Endometrial	Non-Hispanic	White	0.741	0.000	0.259
TCGA-BG-A0VZ	Endometrial	Non-Hispanic	White	0.638	0.034	0.328
TCGA-BG-A0W1	Endometrial	Non-Hispanic	White	0.444	0.018	0.538
TCGA-BG-A0W2	Endometrial	Unknown	White	0.733	0.096	0.171
TCGA-BG-A0YU	Endometrial	Non-Hispanic	White	0.827	0.000	0.173
TCGA-BG-A186	Endometrial	Non-Hispanic	Black	0.443	0.167	0.391
TCGA-BG-A187	Endometrial	Non-Hispanic	White	0.181	0.000	0.819
TCGA-BG-A18A	Endometrial	Non-Hispanic	White	0.304	0.000	0.696
TCGA-BG-A18B	Endometrial	Non-Hispanic	White	0.868	0.000	0.132
TCGA-BG-A18C	Endometrial	Non-Hispanic	White	0.350	0.115	0.535
TCGA-BG-A220	Endometrial	Non-Hispanic	White	1.000	0.000	0.000
TCGA-BG-A221	Endometrial	Non-Hispanic	White	0.752	0.000	0.248
TCGA-BG-A222	Endometrial	Non-Hispanic	White	1.000	0.000	0.000
TCGA-BG-A2AD	Endometrial	Non-Hispanic	White	0.953	0.047	0.000
TCGA-BG-A2AE	Endometrial	Non-Hispanic	White	0.845	0.078	0.077
TCGA-BG-A2L7	Endometrial	Non-Hispanic	White	0.872	0.000	0.128
TCGA-BG-A3EW	Endometrial	Non-Hispanic	White	0.000	1.000	0.000
TCGA-BK-A0C9	Endometrial	Non-Hispanic	White	0.000	0.000	1.000
TCGA-BK-A0CB	Endometrial	Non-Hispanic	White	0.467	0.000	0.533
TCGA-BK-A139	Endometrial	Non-Hispanic	White	0.410	0.103	0.487
TCGA-BK-A13B	Endometrial	Non-Hispanic	Black	0.000	0.813	0.187
TCGA-BK-A13C	Endometrial	Non-Hispanic	Black	0.694	0.000	0.306
TCGA-BK-A4ZD	Endometrial	Non-Hispanic	Black	0.000	0.930	0.070
TCGA-BK-A56F	Endometrial	Non-Hispanic	Black	0.061	0.939	0.000
TCGA-BK-A6W3	Endometrial	Non-Hispanic	Black	0.000	1.000	0.000
TCGA-BK-A6W4	Endometrial	Non-Hispanic	Black	0.000	1.000	0.000
TCGA-BS-A0T9	Endometrial	Non-Hispanic	Pacific	0.210	0.000	0.790
TCGA-BS-A0TA	Endometrial	Hispanic	Pacific	0.106	0.127	0.767
TCGA-BS-A0TC	Endometrial	Non-Hispanic	Asian	0.215	0.000	0.785
TCGA-BS-A0TD	Endometrial	Non-Hispanic	Asian	0.335	0.000	0.665
TCGA-BS-A0TE	Endometrial	Non-Hispanic	Asian	0.000	0.000	1.000
TCGA-BS-A0TG	Endometrial	Non-Hispanic	White	0.362	0.000	0.638

TCGA-BS-A0TI	Endometrial	Non-Hispanic	White	0.000	0.000	1.000
TCGA-BS-A0TJ	Endometrial	Non-Hispanic	Asian	0.679	0.062	0.259
TCGA-BS-A0U5	Endometrial	Non-Hispanic	White	0.681	0.052	0.267
TCGA-BS-A0U7	Endometrial	Non-Hispanic	White	0.873	0.000	0.127
TCGA-BS-A0U8	Endometrial	Non-Hispanic	White	0.545	0.075	0.380
TCGA-BS-A0UA	Endometrial	Non-Hispanic	White	0.432	0.000	0.568
TCGA-BS-A0UF	Endometrial	Non-Hispanic	Asian	0.583	0.109	0.309
TCGA-BS-A0UJ	Endometrial	Non-Hispanic	Asian	0.000	0.000	1.000
TCGA-BS-A0UL	Endometrial	Non-Hispanic	Pacific	0.409	0.152	0.439
TCGA-BS-A0UM	Endometrial	Non-Hispanic	White	0.247	0.000	0.753
TCGA-BS-A0UT	Endometrial	Non-Hispanic	Asian	0.000	0.000	1.000
TCGA-BS-A0UV	Endometrial	Non-Hispanic	Pacific	0.445	0.110	0.445
TCGA-BS-A0V4	Endometrial	Non-Hispanic	White	0.000	0.000	1.000
TCGA-BS-A0V6	Endometrial	Non-Hispanic	Asian	0.401	0.074	0.525
TCGA-BS-A0V7	Endometrial	Non-Hispanic	Pacific	0.000	0.000	1.000
TCGA-BS-A0V8	Endometrial	Non-Hispanic	Pacific	0.000	0.000	1.000
TCGA-BS-A0VI	Endometrial	Non-Hispanic	White	0.000	0.000	1.000
TCGA-BS-A0WQ	Endometrial	Non-Hispanic	Asian	0.423	0.147	0.429
TCGA-D1-A0ZN	Endometrial	Non-Hispanic	White	1.000	0.000	0.000
TCGA-D1-A0ZO	Endometrial	Non-Hispanic	White	0.884	0.000	0.116
TCGA-D1-A0ZQ	Endometrial	Non-Hispanic	White	0.781	0.172	0.047
TCGA-D1-A0ZR	Endometrial	Non-Hispanic	White	0.917	0.083	0.000
TCGA-D1-A0ZS	Endometrial	Non-Hispanic	White	0.962	0.021	0.017
TCGA-D1-A0ZU	Endometrial	Non-Hispanic	White	1.000	0.000	0.000
TCGA-D1-A0ZV	Endometrial	Non-Hispanic	White	0.592	0.027	0.381
TCGA-D1-A101	Endometrial	Non-Hispanic	White	0.217	0.000	0.783
TCGA-D1-A102	Endometrial	Non-Hispanic	White	0.881	0.119	0.000
TCGA-D1-A103	Endometrial	Non-Hispanic	White	0.911	0.089	0.000
TCGA-D1-A15W	Endometrial	Non-Hispanic	White	0.999	0.001	0.000
TCGA-D1-A15Z	Endometrial	Non-Hispanic	White	0.806	0.000	0.194
TCGA-D1-A160	Endometrial	Non-Hispanic	White	0.443	0.000	0.557
TCGA-D1-A161	Endometrial	Non-Hispanic	White	0.670	0.078	0.252
TCGA-D1-A162	Endometrial	Non-Hispanic	White	0.848	0.000	0.152
TCGA-D1-A163	Endometrial	Non-Hispanic	White	0.864	0.000	0.136
TCGA-D1-A165	Endometrial	Non-Hispanic	White	0.906	0.000	0.094
TCGA-D1-A167	Endometrial	Non-Hispanic	White	0.932	0.000	0.068
TCGA-D1-A168	Endometrial	Non-Hispanic	White	0.000	0.000	1.000
TCGA-D1-A169	Endometrial	Non-Hispanic	White	0.027	0.000	0.973
TCGA-D1-A16B	Endometrial	Non-Hispanic	White	0.974	0.000	0.026
TCGA-D1-A16D	Endometrial	Non-Hispanic	White	0.815	0.000	0.185
TCGA-D1-A16E	Endometrial	Non-Hispanic	White	0.776	0.000	0.224
TCGA-D1-A16F	Endometrial	Non-Hispanic	White	0.777	0.021	0.202

TCGA-D1-A16J	Endometrial	Non-Hispanic	White	0.311	0.000	0.689
TCGA-D1-A16N	Endometrial	Non-Hispanic	White	0.016	0.000	0.984
TCGA-D1-A16O	Endometrial	Non-Hispanic	White	0.879	0.000	0.121
TCGA-D1-A16Q	Endometrial	Non-Hispanic	White	0.089	0.000	0.911
TCGA-D1-A16R	Endometrial	Non-Hispanic	White	0.419	0.000	0.581
TCGA-D1-A16V	Endometrial	Non-Hispanic	White	0.369	0.000	0.631
TCGA-D1-A16X	Endometrial	Non-Hispanic	White	0.057	0.000	0.943
TCGA-D1-A16Y	Endometrial	Non-Hispanic	White	0.000	0.000	1.000
TCGA-D1-A174	Endometrial	Non-Hispanic	White	0.000	0.000	1.000
TCGA-D1-A175	Endometrial	Non-Hispanic	White	0.840	0.082	0.078
TCGA-D1-A176	Endometrial	Non-Hispanic	White	0.507	0.000	0.493
TCGA-D1-A177	Endometrial	Non-Hispanic	White	0.459	0.049	0.492
TCGA-D1-A17A	Endometrial	Non-Hispanic	White	0.683	0.000	0.317
TCGA-D1-A17B	Endometrial	Non-Hispanic	White	0.189	0.000	0.811
TCGA-D1-A17C	Endometrial	Non-Hispanic	White	0.068	0.000	0.932
TCGA-D1-A17D	Endometrial	Non-Hispanic	White	0.000	0.000	1.000
TCGA-D1-A17F	Endometrial	Non-Hispanic	White	0.180	0.000	0.820
TCGA-D1-A17H	Endometrial	Non-Hispanic	White	0.445	0.000	0.555
TCGA-D1-A17K	Endometrial	Non-Hispanic	White	0.230	0.000	0.770
TCGA-D1-A17L	Endometrial	Non-Hispanic	White	0.291	0.000	0.709
TCGA-D1-A17M	Endometrial	Non-Hispanic	White	0.186	0.000	0.814
TCGA-D1-A17N	Endometrial	Non-Hispanic	Unknown	0.000	0.000	1.000
TCGA-D1-A17Q	Endometrial	Non-Hispanic	White	0.294	0.000	0.706
TCGA-D1-A17R	Endometrial	Non-Hispanic	White	0.302	0.000	0.698
TCGA-D1-A17S	Endometrial	Non-Hispanic	White	0.060	0.007	0.933
TCGA-D1-A17T	Endometrial	Non-Hispanic	White	0.508	0.000	0.492
TCGA-D1-A17U	Endometrial	Non-Hispanic	White	0.660	0.000	0.340
TCGA-D1-A1NS	Endometrial	Non-Hispanic	White	0.627	0.049	0.324
TCGA-D1-A1NY	Endometrial	Non-Hispanic	White	0.944	0.000	0.056
TCGA-D1-A1NZ	Endometrial	Non-Hispanic	White	0.739	0.064	0.197
TCGA-D1-A1O0	Endometrial	Non-Hispanic	White	0.452	0.173	0.375
TCGA-D1-A1O5	Endometrial	Non-Hispanic	White	0.488	0.091	0.422
TCGA-D1-A1O7	Endometrial	Non-Hispanic	White	0.756	0.025	0.220
TCGA-D1-A1O8	Endometrial	Non-Hispanic	White	0.068	0.106	0.825
TCGA-D1-A2G5	Endometrial	Non-Hispanic	White	0.854	0.124	0.022
TCGA-D1-A2G6	Endometrial	Non-Hispanic	White	1.000	0.000	0.000
TCGA-D1-A3DA	Endometrial	Non-Hispanic	White	0.088	0.912	0.000
TCGA-D1-A3DG	Endometrial	Non-Hispanic	White	0.000	0.687	0.313
TCGA-D1-A3DH	Endometrial	Non-Hispanic	White	0.000	0.575	0.425
TCGA-DF-A2KN	Endometrial	Unknown	Unknown	0.091	0.909	0.000
TCGA-DF-A2KS	Endometrial	Unknown	Unknown	0.470	0.015	0.515
TCGA-DF-A2KU	Endometrial	Unknown	Unknown	0.000	0.330	0.670

TCGA-DF-A2KV	Endometrial	Unknown	Unknown	0.000	0.398	0.602
TCGA-DF-A2KY	Endometrial	Unknown	Unknown	0.000	0.420	0.580
TCGA-DF-A2KZ	Endometrial	Unknown	Unknown	0.057	0.411	0.533
TCGA-DF-A2L0	Endometrial	Unknown	Unknown	0.000	0.000	1.000
TCGA-DI-A0WH	Endometrial	Non-Hispanic	White	0.329	0.065	0.606
TCGA-DI-A1BY	Endometrial	Non-Hispanic	White	0.000	0.000	1.000
TCGA-DI-A1NO	Endometrial	Non-Hispanic	Black	0.287	0.112	0.601
TCGA-DI-A2QU	Endometrial	Non-Hispanic	White	0.275	0.000	0.725
TCGA-E6-A1LX	Endometrial	Non-Hispanic	White	0.215	0.000	0.785
TCGA-E6-A1M0	Endometrial	Non-Hispanic	White	0.949	0.000	0.051
TCGA-E6-A2P9	Endometrial	Non-Hispanic	White	0.198	0.802	0.000
TCGA-EC-A1NJ	Endometrial	Non-Hispanic	White	0.804	0.043	0.153
TCGA-EC-A1QX	Endometrial	Non-Hispanic	White	0.741	0.000	0.259
TCGA-EC-A24G	Endometrial	Non-Hispanic	White	0.897	0.000	0.103
TCGA-EO-A1Y7	Endometrial	Unknown	Unknown	0.000	0.663	0.337
TCGA-EO-A22R	Endometrial	Unknown	Unknown	0.532	0.117	0.350
TCGA-EO-A22S	Endometrial	Unknown	Unknown	0.752	0.000	0.248
TCGA-EO-A22T	Endometrial	Non-Hispanic	Asian	1.000	0.000	0.000
TCGA-EO-A22U	Endometrial	Unknown	White	0.166	0.834	0.000
TCGA-EO-A22X	Endometrial	Unknown	Unknown	0.006	0.773	0.221
TCGA-EO-A22Y	Endometrial	Non-Hispanic	White	0.075	0.919	0.007
TCGA-EO-A3AS	Endometrial	Unknown	Unknown	0.215	0.750	0.035
TCGA-EO-A3AU	Endometrial	Unknown	Unknown	0.200	0.800	0.000
TCGA-EO-A3AY	Endometrial	Unknown	Unknown	0.000	0.159	0.841
TCGA-EO-A3B0	Endometrial	Unknown	Asian	0.000	0.758	0.242
TCGA-EO-A3KX	Endometrial	Hispanic	White	0.000	0.907	0.093
TCGA-EO-A3L0	Endometrial	Non-Hispanic	White	0.000	1.000	0.000
TCGA-EY-A1G7	Endometrial	Non-Hispanic	White	0.870	0.000	0.130
TCGA-EY-A1G8	Endometrial	Non-Hispanic	White	0.394	0.000	0.606
TCGA-EY-A1GC	Endometrial	Non-Hispanic	White	0.222	0.000	0.778
TCGA-EY-A1GD	Endometrial	Non-Hispanic	Black	0.594	0.119	0.287
TCGA-EY-A1GE	Endometrial	Non-Hispanic	White	0.977	0.023	0.000
TCGA-EY-A1GF	Endometrial	Non-Hispanic	Black	0.776	0.224	0.000
TCGA-EY-A1GH	Endometrial	Non-Hispanic	White	0.946	0.054	0.000
TCGA-EY-A1GI	Endometrial	Non-Hispanic	White	0.919	0.000	0.081
TCGA-EY-A1GK	Endometrial	Non-Hispanic	White	0.686	0.090	0.224
TCGA-EY-A1GL	Endometrial	Unknown	White	0.272	0.600	0.128
TCGA-EY-A1GP	Endometrial	Non-Hispanic	Black	0.000	0.906	0.094
TCGA-EY-A1GQ	Endometrial	Non-Hispanic	White	0.303	0.083	0.614
TCGA-EY-A1GR	Endometrial	Non-Hispanic	Black	0.086	0.166	0.747
TCGA-EY-A1GT	Endometrial	Non-Hispanic	White	0.858	0.142	0.000
TCGA-EY-A1GU	Endometrial	Non-Hispanic	Black	0.716	0.284	0.000

TCGA-EY-A1GW	Endometrial	Hispanic	White	0.171	0.006	0.823
TCGA-EY-A1GX	Endometrial	Non-Hispanic	White	0.061	0.939	0.000
TCGA-EY-A1H0	Endometrial	Non-Hispanic	Black	0.501	0.129	0.370
TCGA-EY-A214	Endometrial	Unknown	White	0.460	0.000	0.540
TCGA-EY-A215	Endometrial	Non-Hispanic	White	0.722	0.074	0.204
TCGA-EY-A2OM	Endometrial	Non-Hispanic	White	0.945	0.000	0.055
TCGA-EY-A2OP	Endometrial	Non-Hispanic	White	0.320	0.680	0.000
TCGA-EY-A2OQ	Endometrial	Non-Hispanic	White	0.255	0.745	0.000
TCGA-EY-A548	Endometrial	Non-Hispanic	Black	0.000	1.000	0.000
TCGA-EY-A549	Endometrial	Non-Hispanic	Black	0.000	1.000	0.000
TCGA-EY-A54A	Endometrial	Non-Hispanic	Black	0.000	1.000	0.000
TCGA-EY-A5W2	Endometrial	Non-Hispanic	Black	0.000	0.594	0.406
TCGA-EY-A72D	Endometrial	Non-Hispanic	Black	0.000	0.759	0.241
TCGA-FI-A2CX	Endometrial	Non-Hispanic	White	0.494	0.138	0.367
TCGA-FI-A2D0	Endometrial	Non-Hispanic	White	0.943	0.007	0.050
TCGA-FI-A2D4	Endometrial	Unknown	White	0.525	0.000	0.475
TCGA-FI-A2D5	Endometrial	Non-Hispanic	White	0.893	0.000	0.107
TCGA-FI-A2D6	Endometrial	Non-Hispanic	White	0.922	0.044	0.034
TCGA-FI-A2F4	Endometrial	Non-Hispanic	Black	0.614	0.055	0.331
TCGA-FI-A2F9	Endometrial	Non-Hispanic	White	0.475	0.000	0.525
TCGA-H5-A2HR	Endometrial	Non-Hispanic	Black	0.000	0.009	0.991
TCGA-PG-A916	Endometrial	Non-Hispanic	Black	0.000	1.000	0.000
TCGA-PG-A917	Endometrial	Non-Hispanic	Black	0.000	0.704	0.296
TCGA-QF-A5YT	Endometrial	Non-Hispanic	Black	0.000	0.578	0.422
TCGA-QS-A5YQ	Endometrial	Non-Hispanic	Black	0.000	0.506	0.494
TCGA-QS-A744	Endometrial	Non-Hispanic	Black	0.000	1.000	0.000
TCGA-SJ-A6ZI	Endometrial	Non-Hispanic	Black	0.000	1.000	0.000
TCGA-SJ-A6ZJ	Endometrial	Non-Hispanic	Black	0.000	0.777	0.223
TCGA-SL-A6J9	Endometrial	Non-Hispanic	Black	0.000	0.651	0.349
TCGA-SL-A6JA	Endometrial	Non-Hispanic	Black	0.000	0.853	0.147

Table 4. Q-matrix with 2 cluster solution at the subpopulations structure analysis of endometrial cancer TCGA patients. For each patient there is also information about tumor type and self-declared race and ethnicity.

ID	Tumor type	Ethnicity	Race	Cluster 1	Cluster 2
TCGA-2E-A9G8	Endometrial	Non-Hispanic	Black	0.783	0.217
TCGA-4E-A92E	Endometrial	Non-Hispanic	Black	1.000	0.000
TCGA-5B-A90C	Endometrial	Non-Hispanic	Black	1.000	0.000
TCGA-A5-A0G9	Endometrial	Unknown	White	0.000	1.000
TCGA-A5-A0GA	Endometrial	Non-Hispanic	White	0.255	0.745
TCGA-A5-A0GB	Endometrial	Unknown	White	0.172	0.828
TCGA-A5-A0GD	Endometrial	Unknown	White	0.248	0.752
TCGA-A5-A0GE	Endometrial	Non-Hispanic	Asian	0.154	0.846
TCGA-A5-A0GG	Endometrial	Non-Hispanic	Black	0.261	0.739
TCGA-A5-A0GH	Endometrial	Non-Hispanic	White	0.317	0.683
TCGA-A5-A0GI	Endometrial	Non-Hispanic	White	0.000	1.000
TCGA-A5-A0GJ	Endometrial	Non-Hispanic	White	0.000	1.000
TCGA-A5-A0GM	Endometrial	Non-Hispanic	White	0.020	0.980
TCGA-A5-A0GN	Endometrial	Non-Hispanic	White	0.000	1.000
TCGA-A5-A0GP	Endometrial	Non-Hispanic	Asian	0.050	0.950
TCGA-A5-A0GQ	Endometrial	Non-Hispanic	White	0.130	0.870
TCGA-A5-A0GR	Endometrial	Non-Hispanic	Black	0.000	1.000
TCGA-A5-A0GU	Endometrial	Unknown	White	0.310	0.690
TCGA-A5-A0GV	Endometrial	Non-Hispanic	White	0.090	0.910
TCGA-A5-A0GW	Endometrial	Non-Hispanic	Asian	0.214	0.786
TCGA-A5-A0GX	Endometrial	Unknown	White	0.000	1.000
TCGA-A5-A0R7	Endometrial	Non-Hispanic	White	0.135	0.865
TCGA-A5-A0R8	Endometrial	Non-Hispanic	White	0.000	1.000
TCGA-A5-A0R9	Endometrial	Non-Hispanic	White	0.136	0.864
TCGA-A5-A0RA	Endometrial	Non-Hispanic	White	0.000	1.000
TCGA-A5-A0VO	Endometrial	Non-Hispanic	White	0.189	0.811
TCGA-A5-A0VP	Endometrial	Non-Hispanic	White	0.335	0.665
TCGA-A5-A0VQ	Endometrial	Hispanic	Unknown	0.221	0.779
TCGA-A5-A1OJ	Endometrial	Non-Hispanic	Asian	0.166	0.834
TCGA-A5-A1OK	Endometrial	Non-Hispanic	White	0.382	0.618
TCGA-A5-A2K5	Endometrial	Non-Hispanic	White	0.412	0.588
TCGA-A5-A2K7	Endometrial	Hispanic	Unknown	0.840	0.160
TCGA-A5-A3LO	Endometrial	Non-Hispanic	White	0.905	0.095
TCGA-A5-A7WJ	Endometrial	Non-Hispanic	Black	1.000	0.000
TCGA-A5-AB3J	Endometrial	Non-Hispanic	Black	1.000	0.000
TCGA-AJ-A23O	Endometrial	Non-Hispanic	White	0.237	0.763
TCGA-AJ-A2QK	Endometrial	Hispanic	White	0.225	0.775
TCGA-AJ-A2QL	Endometrial	Non-Hispanic	White	0.212	0.788

TCGA-AJ-A2QN	Endometrial	Unknown	White	0.251	0.749
TCGA-AJ-A2QO	Endometrial	Unknown	White	0.888	0.112
TCGA-AJ-A3BH	Endometrial	Non-Hispanic	White	0.886	0.114
TCGA-AJ-A3BI	Endometrial	Non-Hispanic	White	0.039	0.961
TCGA-AJ-A3BK	Endometrial	Non-Hispanic	White	0.641	0.359
TCGA-AJ-A3EK	Endometrial	Hispanic	White	1.000	0.000
TCGA-AJ-A3EL	Endometrial	Unknown	Pacific	0.632	0.368
TCGA-AJ-A3EM	Endometrial	Unknown	White	0.938	0.062
TCGA-AJ-A3I9	Endometrial	Non-Hispanic	White	1.000	0.000
TCGA-AJ-A3NC	Endometrial	Unknown	White	1.000	0.000
TCGA-AJ-A3NE	Endometrial	Non-Hispanic	White	1.000	0.000
TCGA-AJ-A3OJ	Endometrial	Non-Hispanic	White	0.812	0.188
TCGA-AJ-A3OL	Endometrial	Non-Hispanic	White	1.000	0.000
TCGA-AJ-A5DV	Endometrial	Non-Hispanic	Black	1.000	0.000
TCGA-AJ-A8CT	Endometrial	Unknown	Black	0.944	0.056
TCGA-AJ-A8CV	Endometrial	Unknown	Black	0.990	0.010
TCGA-AJ-A8CW	Endometrial	Non-Hispanic	Black	1.000	0.000
TCGA-AP-A051	Endometrial	Unknown	White	0.373	0.627
TCGA-AP-A053	Endometrial	Non-Hispanic	White	0.128	0.872
TCGA-AP-A054	Endometrial	Non-Hispanic	White	0.485	0.515
TCGA-AP-A056	Endometrial	Unknown	White	0.132	0.868
TCGA-AP-A059	Endometrial	Non-Hispanic	White	0.338	0.662
TCGA-AP-A05N	Endometrial	Unknown	White	0.000	1.000
TCGA-AP-A05O	Endometrial	Unknown	White	0.252	0.748
TCGA-AP-A05P	Endometrial	Non-Hispanic	Asian	0.420	0.580
TCGA-AP-A0LD	Endometrial	Unknown	White	0.051	0.949
TCGA-AP-A0LE	Endometrial	Unknown	White	0.000	1.000
TCGA-AP-A0LF	Endometrial	Non-Hispanic	White	0.000	1.000
TCGA-AP-A0LG	Endometrial	Unknown	White	0.319	0.681
TCGA-AP-A0LJ	Endometrial	Unknown	White	0.000	1.000
TCGA-AP-A0LL	Endometrial	Unknown	White	0.000	1.000
TCGA-AP-A0LM	Endometrial	Unknown	Black	0.163	0.837
TCGA-AP-A0LN	Endometrial	Unknown	Black	0.000	1.000
TCGA-AP-A0LO	Endometrial	Non-Hispanic	White	0.000	1.000
TCGA-AP-A0LP	Endometrial	Unknown	White	0.006	0.994
TCGA-AP-A0LS	Endometrial	Unknown	White	0.165	0.835
TCGA-AP-A0LT	Endometrial	Unknown	White	0.197	0.803
TCGA-AP-A0LV	Endometrial	Unknown	White	0.277	0.723
TCGA-AP-A1DH	Endometrial	Unknown	White	0.116	0.884
TCGA-AP-A1DK	Endometrial	Unknown	White	0.014	0.986
TCGA-AP-A1DM	Endometrial	Unknown	White	0.156	0.844
TCGA-AP-A1DO	Endometrial	Non-Hispanic	White	0.144	0.856

TCGA-AP-A1DP	Endometrial	Unknown	White	0.058	0.942
TCGA-AP-A1DR	Endometrial	Unknown	Black	0.190	0.810
TCGA-AP-A1DV	Endometrial	Non-Hispanic	White	0.284	0.716
TCGA-AP-A1E0	Endometrial	Unknown	White	0.342	0.658
TCGA-AP-A1E1	Endometrial	Unknown	White	0.234	0.766
TCGA-AP-A1E3	Endometrial	Unknown	White	0.102	0.898
TCGA-AP-A1E4	Endometrial	Unknown	White	0.284	0.716
TCGA-AW-A1PO	Endometrial	Non-Hispanic	White	0.365	0.635
TCGA-AX-A05S	Endometrial	Non-Hispanic	White	0.000	1.000
TCGA-AX-A05T	Endometrial	Unknown	White	0.000	1.000
TCGA-AX-A05U	Endometrial	Non-Hispanic	American Indian	0.179	0.821
TCGA-AX-A05W	Endometrial	Non-Hispanic	White	0.157	0.843
TCGA-AX-A05Y	Endometrial	Non-Hispanic	White	0.000	1.000
TCGA-AX-A05Z	Endometrial	Hispanic	Unknown	0.000	1.000
TCGA-AX-A060	Endometrial	Non-Hispanic	American Indian	0.000	1.000
TCGA-AX-A062	Endometrial	Unknown	White	0.000	1.000
TCGA-AX-A063	Endometrial	Non-Hispanic	White	0.000	1.000
TCGA-AX-A064	Endometrial	Unknown	White	0.000	1.000
TCGA-AX-A06B	Endometrial	Non-Hispanic	White	0.262	0.738
TCGA-AX-A06D	Endometrial	Unknown	White	0.164	0.836
TCGA-AX-A06F	Endometrial	Non-Hispanic	White	0.007	0.993
TCGA-AX-A06H	Endometrial	Non-Hispanic	American Indian	0.075	0.925
TCGA-AX-A06J	Endometrial	Non-Hispanic	White	0.000	1.000
TCGA-AX-A06L	Endometrial	Unknown	White	0.000	1.000
TCGA-AX-A0IS	Endometrial	Non-Hispanic	White	0.117	0.883
TCGA-AX-A0IZ	Endometrial	Non-Hispanic	White	0.214	0.786
TCGA-AX-A0J0	Endometrial	Non-Hispanic	White	0.363	0.637
TCGA-AX-A0J1	Endometrial	Non-Hispanic	White	0.307	0.693
TCGA-AX-A1C4	Endometrial	Non-Hispanic	White	0.335	0.665
TCGA-AX-A1C5	Endometrial	Non-Hispanic	White	0.258	0.742
TCGA-AX-A1C9	Endometrial	Non-Hispanic	White	0.242	0.758
TCGA-AX-A1CE	Endometrial	Non-Hispanic	White	0.055	0.945
TCGA-AX-A1CF	Endometrial	Non-Hispanic	White	0.245	0.755
TCGA-AX-A1CI	Endometrial	Non-Hispanic	White	0.092	0.908
TCGA-AX-A1CJ	Endometrial	Non-Hispanic	Black	0.000	1.000
TCGA-AX-A1CK	Endometrial	Non-Hispanic	White	0.195	0.805
TCGA-AX-A1CN	Endometrial	Non-Hispanic	White	0.378	0.622
TCGA-AX-A2H7	Endometrial	Non-Hispanic	White	0.122	0.878
TCGA-AX-A2H8	Endometrial	Non-Hispanic	White	0.264	0.736

TCGA-AX-A2HA	Endometrial	Unknown	White	0.088	0.912
TCGA-AX-A2HC	Endometrial	Non-Hispanic	Black	0.495	0.505
TCGA-AX-A2HD	Endometrial	Non-Hispanic	White	0.378	0.622
TCGA-AX-A2HG	Endometrial	Hispanic	White	0.219	0.781
TCGA-AX-A2HH	Endometrial	Non-Hispanic	White	0.947	0.053
TCGA-AX-A2HJ	Endometrial	Non-Hispanic	Asian	0.363	0.637
TCGA-AX-A2HK	Endometrial	Unknown	White	0.332	0.668
TCGA-AX-A2IN	Endometrial	Unknown	White	0.846	0.154
TCGA-AX-A3FS	Endometrial	Non-Hispanic	White	0.906	0.094
TCGA-AX-A3FT	Endometrial	Non-Hispanic	White	1.000	0.000
TCGA-AX-A3FV	Endometrial	Non-Hispanic	White	0.530	0.470
TCGA-AX-A3FW	Endometrial	Non-Hispanic	White	0.655	0.345
TCGA-AX-A3FX	Endometrial	Non-Hispanic	White	0.575	0.425
TCGA-AX-A3FZ	Endometrial	Non-Hispanic	White	0.905	0.095
TCGA-AX-A3G1	Endometrial	Non-Hispanic	White	0.583	0.417
TCGA-AX-A3G8	Endometrial	Unknown	White	0.354	0.646
TCGA-AX-A3G9	Endometrial	Non-Hispanic	White	0.766	0.234
TCGA-AX-A3GB	Endometrial	Non-Hispanic	White	0.713	0.287
TCGA-B5-A0JR	Endometrial	Unknown	White	0.907	0.093
TCGA-B5-A0JS	Endometrial	Unknown	White	0.131	0.869
TCGA-B5-A0JT	Endometrial	Unknown	White	0.351	0.649
TCGA-B5-A0JV	Endometrial	Unknown	Black	0.218	0.782
TCGA-B5-A0JX	Endometrial	Unknown	White	0.271	0.729
TCGA-B5-A0JY	Endometrial	Unknown	White	0.127	0.873
TCGA-B5-A0JZ	Endometrial	Unknown	White	0.184	0.816
TCGA-B5-A0K0	Endometrial	Unknown	White	0.385	0.615
TCGA-B5-A0K1	Endometrial	Unknown	White	0.129	0.871
TCGA-B5-A0K2	Endometrial	Unknown	White	0.000	1.000
TCGA-B5-A0K3	Endometrial	Unknown	Black	0.055	0.945
TCGA-B5-A0K4	Endometrial	Unknown	Unknown	0.000	1.000
TCGA-B5-A0K6	Endometrial	Unknown	White	0.000	1.000
TCGA-B5-A0K7	Endometrial	Unknown	White	0.116	0.884
TCGA-B5-A0K9	Endometrial	Unknown	White	1.000	0.000
TCGA-B5-A11E	Endometrial	Unknown	White	0.213	0.787
TCGA-B5-A11F	Endometrial	Unknown	White	0.122	0.878
TCGA-B5-A11G	Endometrial	Unknown	Black	0.091	0.909
TCGA-B5-A11H	Endometrial	Unknown	White	0.229	0.771
TCGA-B5-A11I	Endometrial	Unknown	Black	0.348	0.652
TCGA-B5-A11J	Endometrial	Unknown	White	0.263	0.737
TCGA-B5-A11M	Endometrial	Unknown	White	0.455	0.545
TCGA-B5-A11N	Endometrial	Unknown	White	0.177	0.823
TCGA-B5-A11O	Endometrial	Unknown	White	0.285	0.715

TCGA-B5-A11Q	Endometrial	Unknown	White	0.058	0.942
TCGA-B5-A11R	Endometrial	Unknown	Black	0.939	0.061
TCGA-B5-A11S	Endometrial	Unknown	White	0.349	0.651
TCGA-B5-A11U	Endometrial	Unknown	Black	0.347	0.653
TCGA-B5-A11V	Endometrial	Unknown	Black	0.235	0.765
TCGA-B5-A11W	Endometrial	Unknown	White	0.130	0.870
TCGA-B5-A11X	Endometrial	Unknown	Black	0.267	0.733
TCGA-B5-A11Y	Endometrial	Unknown	White	0.163	0.837
TCGA-B5-A11Z	Endometrial	Unknown	White	0.351	0.649
TCGA-B5-A121	Endometrial	Unknown	White	0.261	0.739
TCGA-B5-A1MR	Endometrial	Unknown	White	0.000	1.000
TCGA-B5-A1MV	Endometrial	Unknown	White	0.344	0.656
TCGA-B5-A1MW	Endometrial	Unknown	White	1.000	0.000
TCGA-B5-A1MX	Endometrial	Unknown	White	0.366	0.634
TCGA-B5-A1MZ	Endometrial	Unknown	White	0.231	0.769
TCGA-B5-A3F9	Endometrial	Unknown	Black	1.000	0.000
TCGA-B5-A3FA	Endometrial	Unknown	White	0.851	0.149
TCGA-B5-A3FB	Endometrial	Unknown	Black	0.948	0.052
TCGA-B5-A3FC	Endometrial	Non-Hispanic	Black	0.793	0.207
TCGA-B5-A3FD	Endometrial	Unknown	Black	0.651	0.349
TCGA-B5-A3FH	Endometrial	Unknown	Black	0.882	0.118
TCGA-B5-A5OC	Endometrial	Unknown	Black	1.000	0.000
TCGA-BG-A0LW	Endometrial	Non-Hispanic	White	0.302	0.698
TCGA-BG-A0LX	Endometrial	Non-Hispanic	White	0.290	0.710
TCGA-BG-A0M0	Endometrial	Non-Hispanic	White	0.182	0.818
TCGA-BG-A0M2	Endometrial	Non-Hispanic	White	0.000	1.000
TCGA-BG-A0M3	Endometrial	Non-Hispanic	White	0.155	0.845
TCGA-BG-A0M4	Endometrial	Non-Hispanic	White	0.000	1.000
TCGA-BG-A0M7	Endometrial	Non-Hispanic	White	0.000	1.000
TCGA-BG-A0M8	Endometrial	Non-Hispanic	White	0.000	1.000
TCGA-BG-A0M9	Endometrial	Non-Hispanic	White	0.000	1.000
TCGA-BG-A0MA	Endometrial	Non-Hispanic	White	0.307	0.693
TCGA-BG-A0MC	Endometrial	Non-Hispanic	White	0.000	1.000
TCGA-BG-A0MG	Endometrial	Non-Hispanic	White	0.003	0.997
TCGA-BG-A0MH	Endometrial	Unknown	White	0.239	0.761
TCGA-BG-A0MI	Endometrial	Non-Hispanic	White	0.000	1.000
TCGA-BG-A0MK	Endometrial	Non-Hispanic	Black	0.662	0.338
TCGA-BG-A0MO	Endometrial	Non-Hispanic	White	0.029	0.971
TCGA-BG-A0MQ	Endometrial	Non-Hispanic	White	0.173	0.827
TCGA-BG-A0MS	Endometrial	Non-Hispanic	White	0.244	0.756
TCGA-BG-A0MT	Endometrial	Non-Hispanic	White	0.253	0.747
TCGA-BG-A0MU	Endometrial	Non-Hispanic	White	0.000	1.000

TCGA-BG-A0RY	Endometrial	Non-Hispanic	White	0.076	0.924
TCGA-BG-A0VT	Endometrial	Non-Hispanic	White	0.201	0.799
TCGA-BG-A0VV	Endometrial	Non-Hispanic	White	0.164	0.836
TCGA-BG-A0VW	Endometrial	Non-Hispanic	White	0.000	1.000
TCGA-BG-A0VX	Endometrial	Non-Hispanic	White	0.187	0.813
TCGA-BG-A0VZ	Endometrial	Non-Hispanic	White	0.250	0.750
TCGA-BG-A0W1	Endometrial	Non-Hispanic	White	0.128	0.872
TCGA-BG-A0W2	Endometrial	Unknown	White	0.309	0.691
TCGA-BG-A0YU	Endometrial	Non-Hispanic	White	0.303	0.697
TCGA-BG-A186	Endometrial	Non-Hispanic	Black	0.339	0.661
TCGA-BG-A187	Endometrial	Non-Hispanic	White	0.000	1.000
TCGA-BG-A18A	Endometrial	Non-Hispanic	White	0.000	1.000
TCGA-BG-A18B	Endometrial	Non-Hispanic	White	0.281	0.719
TCGA-BG-A18C	Endometrial	Non-Hispanic	White	0.268	0.732
TCGA-BG-A220	Endometrial	Non-Hispanic	White	0.434	0.566
TCGA-BG-A221	Endometrial	Non-Hispanic	White	0.207	0.793
TCGA-BG-A222	Endometrial	Non-Hispanic	White	0.472	0.528
TCGA-BG-A2AD	Endometrial	Non-Hispanic	White	0.527	0.473
TCGA-BG-A2AE	Endometrial	Non-Hispanic	White	0.363	0.637
TCGA-BG-A2L7	Endometrial	Non-Hispanic	White	0.323	0.677
TCGA-BG-A3EW	Endometrial	Non-Hispanic	White	1.000	0.000
TCGA-BK-A0C9	Endometrial	Non-Hispanic	White	0.000	1.000
TCGA-BK-A0CB	Endometrial	Non-Hispanic	White	0.072	0.928
TCGA-BK-A139	Endometrial	Non-Hispanic	White	0.276	0.724
TCGA-BK-A13B	Endometrial	Non-Hispanic	Black	0.906	0.094
TCGA-BK-A13C	Endometrial	Non-Hispanic	Black	0.255	0.745
TCGA-BK-A4ZD	Endometrial	Non-Hispanic	Black	1.000	0.000
TCGA-BK-A56F	Endometrial	Non-Hispanic	Black	1.000	0.000
TCGA-BK-A6W3	Endometrial	Non-Hispanic	Black	1.000	0.000
TCGA-BK-A6W4	Endometrial	Non-Hispanic	Black	1.000	0.000
TCGA-BS-A0T9	Endometrial	Non-Hispanic	Pacific	0.000	1.000
TCGA-BS-A0TA	Endometrial	Hispanic	Pacific	0.146	0.854
TCGA-BS-A0TC	Endometrial	Non-Hispanic	Asian	0.000	1.000
TCGA-BS-A0TD	Endometrial	Non-Hispanic	Asian	0.039	0.961
TCGA-BS-A0TE	Endometrial	Non-Hispanic	Asian	0.000	1.000
TCGA-BS-A0TG	Endometrial	Non-Hispanic	White	0.043	0.957
TCGA-BS-A0TI	Endometrial	Non-Hispanic	White	0.000	1.000
TCGA-BS-A0TJ	Endometrial	Non-Hispanic	Asian	0.308	0.692
TCGA-BS-A0U5	Endometrial	Non-Hispanic	White	0.284	0.716
TCGA-BS-A0U7	Endometrial	Non-Hispanic	White	0.312	0.688
TCGA-BS-A0U8	Endometrial	Non-Hispanic	White	0.227	0.773
TCGA-BS-A0UA	Endometrial	Non-Hispanic	White	0.092	0.908

TCGA-BS-A0UF	Endometrial	Non-Hispanic	Asian	0.291	0.709
TCGA-BS-A0UJ	Endometrial	Non-Hispanic	Asian	0.000	1.000
TCGA-BS-A0UL	Endometrial	Non-Hispanic	Pacific	0.280	0.720
TCGA-BS-A0UM	Endometrial	Non-Hispanic	White	0.000	1.000
TCGA-BS-A0UT	Endometrial	Non-Hispanic	Asian	0.000	1.000
TCGA-BS-A0UV	Endometrial	Non-Hispanic	Pacific	0.230	0.770
TCGA-BS-A0V4	Endometrial	Non-Hispanic	White	0.000	1.000
TCGA-BS-A0V6	Endometrial	Non-Hispanic	Asian	0.195	0.805
TCGA-BS-A0V7	Endometrial	Non-Hispanic	Pacific	0.000	1.000
TCGA-BS-A0V8	Endometrial	Non-Hispanic	Pacific	0.000	1.000
TCGA-BS-A0VI	Endometrial	Non-Hispanic	White	0.000	1.000
TCGA-BS-A0WQ	Endometrial	Non-Hispanic	Asian	0.262	0.738
TCGA-D1-A0ZN	Endometrial	Non-Hispanic	White	0.402	0.598
TCGA-D1-A0ZO	Endometrial	Non-Hispanic	White	0.338	0.662
TCGA-D1-A0ZQ	Endometrial	Non-Hispanic	White	0.404	0.596
TCGA-D1-A0ZR	Endometrial	Non-Hispanic	White	0.425	0.575
TCGA-D1-A0ZS	Endometrial	Non-Hispanic	White	0.415	0.585
TCGA-D1-A0ZU	Endometrial	Non-Hispanic	White	0.382	0.618
TCGA-D1-A0ZV	Endometrial	Non-Hispanic	White	0.201	0.799
TCGA-D1-A101	Endometrial	Non-Hispanic	White	0.040	0.960
TCGA-D1-A102	Endometrial	Non-Hispanic	White	0.444	0.556
TCGA-D1-A103	Endometrial	Non-Hispanic	White	0.439	0.561
TCGA-D1-A15W	Endometrial	Non-Hispanic	White	0.422	0.578
TCGA-D1-A15Z	Endometrial	Non-Hispanic	White	0.271	0.729
TCGA-D1-A160	Endometrial	Non-Hispanic	White	0.132	0.868
TCGA-D1-A161	Endometrial	Non-Hispanic	White	0.286	0.714
TCGA-D1-A162	Endometrial	Non-Hispanic	White	0.292	0.708
TCGA-D1-A163	Endometrial	Non-Hispanic	White	0.283	0.717
TCGA-D1-A165	Endometrial	Non-Hispanic	White	0.261	0.739
TCGA-D1-A167	Endometrial	Non-Hispanic	White	0.321	0.679
TCGA-D1-A168	Endometrial	Non-Hispanic	White	0.000	1.000
TCGA-D1-A169	Endometrial	Non-Hispanic	White	0.000	1.000
TCGA-D1-A16B	Endometrial	Non-Hispanic	White	0.306	0.694
TCGA-D1-A16D	Endometrial	Non-Hispanic	White	0.291	0.709
TCGA-D1-A16E	Endometrial	Non-Hispanic	White	0.267	0.733
TCGA-D1-A16F	Endometrial	Non-Hispanic	White	0.292	0.708
TCGA-D1-A16J	Endometrial	Non-Hispanic	White	0.074	0.926
TCGA-D1-A16N	Endometrial	Non-Hispanic	White	0.000	1.000
TCGA-D1-A16O	Endometrial	Non-Hispanic	White	0.241	0.759
TCGA-D1-A16Q	Endometrial	Non-Hispanic	White	0.000	1.000
TCGA-D1-A16R	Endometrial	Non-Hispanic	White	0.000	1.000
TCGA-D1-A16V	Endometrial	Non-Hispanic	White	0.117	0.883

TCGA-D1-A16X	Endometrial	Non-Hispanic	White	0.000	1.000
TCGA-D1-A16Y	Endometrial	Non-Hispanic	White	0.000	1.000
TCGA-D1-A174	Endometrial	Non-Hispanic	White	0.000	1.000
TCGA-D1-A175	Endometrial	Non-Hispanic	White	0.427	0.573
TCGA-D1-A176	Endometrial	Non-Hispanic	White	0.113	0.887
TCGA-D1-A177	Endometrial	Non-Hispanic	White	0.191	0.809
TCGA-D1-A17A	Endometrial	Non-Hispanic	White	0.224	0.776
TCGA-D1-A17B	Endometrial	Non-Hispanic	White	0.000	1.000
TCGA-D1-A17C	Endometrial	Non-Hispanic	White	0.000	1.000
TCGA-D1-A17D	Endometrial	Non-Hispanic	White	0.000	1.000
TCGA-D1-A17F	Endometrial	Non-Hispanic	White	0.000	1.000
TCGA-D1-A17H	Endometrial	Non-Hispanic	White	0.093	0.907
TCGA-D1-A17K	Endometrial	Non-Hispanic	White	0.000	1.000
TCGA-D1-A17L	Endometrial	Non-Hispanic	White	0.028	0.972
TCGA-D1-A17M	Endometrial	Non-Hispanic	White	0.000	1.000
TCGA-D1-A17N	Endometrial	Non-Hispanic	Unknown	0.000	1.000
TCGA-D1-A17Q	Endometrial	Non-Hispanic	White	0.000	1.000
TCGA-D1-A17R	Endometrial	Non-Hispanic	White	0.000	1.000
TCGA-D1-A17S	Endometrial	Non-Hispanic	White	0.000	1.000
TCGA-D1-A17T	Endometrial	Non-Hispanic	White	0.051	0.949
TCGA-D1-A17U	Endometrial	Non-Hispanic	White	0.151	0.849
TCGA-D1-A1NS	Endometrial	Non-Hispanic	White	0.221	0.779
TCGA-D1-A1NY	Endometrial	Non-Hispanic	White	0.266	0.734
TCGA-D1-A1NZ	Endometrial	Non-Hispanic	White	0.270	0.730
TCGA-D1-A1O0	Endometrial	Non-Hispanic	White	0.307	0.693
TCGA-D1-A1O5	Endometrial	Non-Hispanic	White	0.240	0.760
TCGA-D1-A1O7	Endometrial	Non-Hispanic	White	0.250	0.750
TCGA-D1-A1O8	Endometrial	Non-Hispanic	White	0.105	0.895
TCGA-D1-A2G5	Endometrial	Non-Hispanic	White	0.418	0.582
TCGA-D1-A2G6	Endometrial	Non-Hispanic	White	0.290	0.710
TCGA-D1-A3DA	Endometrial	Non-Hispanic	White	1.000	0.000
TCGA-D1-A3DG	Endometrial	Non-Hispanic	White	0.745	0.255
TCGA-D1-A3DH	Endometrial	Non-Hispanic	White	0.618	0.382
TCGA-DF-A2KN	Endometrial	Unknown	Unknown	1.000	0.000
TCGA-DF-A2KS	Endometrial	Unknown	Unknown	0.239	0.761
TCGA-DF-A2KU	Endometrial	Unknown	Unknown	0.365	0.635
TCGA-DF-A2KV	Endometrial	Unknown	Unknown	0.399	0.601
TCGA-DF-A2KY	Endometrial	Unknown	Unknown	0.480	0.520
TCGA-DF-A2KZ	Endometrial	Unknown	Unknown	0.440	0.560
TCGA-DF-A2L0	Endometrial	Unknown	Unknown	0.000	1.000
TCGA-DI-A0WH	Endometrial	Non-Hispanic	White	0.125	0.875
TCGA-DI-A1BY	Endometrial	Non-Hispanic	White	0.000	1.000

TCGA-DI-A1NO	Endometrial	Non-Hispanic	Black	0.200	0.800
TCGA-DI-A2QU	Endometrial	Non-Hispanic	White	0.020	0.980
TCGA-E6-A1LX	Endometrial	Non-Hispanic	White	0.000	1.000
TCGA-E6-A1M0	Endometrial	Non-Hispanic	White	0.357	0.643
TCGA-E6-A2P9	Endometrial	Non-Hispanic	White	0.956	0.044
TCGA-EC-A1NJ	Endometrial	Non-Hispanic	White	0.336	0.664
TCGA-EC-A1QX	Endometrial	Non-Hispanic	White	0.219	0.781
TCGA-EC-A24G	Endometrial	Non-Hispanic	White	0.311	0.689
TCGA-EO-A1Y7	Endometrial	Unknown	Unknown	0.741	0.259
TCGA-EO-A22R	Endometrial	Unknown	Unknown	0.333	0.667
TCGA-EO-A22S	Endometrial	Unknown	Unknown	0.287	0.713
TCGA-EO-A22T	Endometrial	Non-Hispanic	Asian	0.433	0.567
TCGA-EO-A22U	Endometrial	Unknown	White	0.966	0.034
TCGA-EO-A22X	Endometrial	Unknown	Unknown	0.819	0.181
TCGA-EO-A22Y	Endometrial	Non-Hispanic	White	0.982	0.018
TCGA-EO-A3AS	Endometrial	Unknown	Unknown	0.887	0.113
TCGA-EO-A3AU	Endometrial	Unknown	Unknown	1.000	0.000
TCGA-EO-A3AY	Endometrial	Unknown	Unknown	0.182	0.818
TCGA-EO-A3B0	Endometrial	Unknown	Asian	0.819	0.181
TCGA-EO-A3KX	Endometrial	Hispanic	White	1.000	0.000
TCGA-EO-A3L0	Endometrial	Non-Hispanic	White	1.000	0.000
TCGA-EY-A1G7	Endometrial	Non-Hispanic	White	0.367	0.633
TCGA-EY-A1G8	Endometrial	Non-Hispanic	White	0.097	0.903
TCGA-EY-A1GC	Endometrial	Non-Hispanic	White	0.068	0.932
TCGA-EY-A1GD	Endometrial	Non-Hispanic	Black	0.340	0.660
TCGA-EY-A1GE	Endometrial	Non-Hispanic	White	0.408	0.592
TCGA-EY-A1GF	Endometrial	Non-Hispanic	Black	0.512	0.488
TCGA-EY-A1GH	Endometrial	Non-Hispanic	White	0.423	0.577
TCGA-EY-A1GI	Endometrial	Non-Hispanic	White	0.391	0.609
TCGA-EY-A1GK	Endometrial	Non-Hispanic	White	0.279	0.721
TCGA-EY-A1GL	Endometrial	Unknown	White	0.738	0.262
TCGA-EY-A1GP	Endometrial	Non-Hispanic	Black	1.000	0.000
TCGA-EY-A1GQ	Endometrial	Non-Hispanic	White	0.143	0.857
TCGA-EY-A1GR	Endometrial	Non-Hispanic	Black	0.194	0.806
TCGA-EY-A1GT	Endometrial	Non-Hispanic	White	0.469	0.531
TCGA-EY-A1GU	Endometrial	Non-Hispanic	Black	0.537	0.463
TCGA-EY-A1GW	Endometrial	Hispanic	White	0.037	0.963
TCGA-EY-A1GX	Endometrial	Non-Hispanic	White	1.000	0.000
TCGA-EY-A1H0	Endometrial	Non-Hispanic	Black	0.317	0.683
TCGA-EY-A214	Endometrial	Unknown	White	0.199	0.801
TCGA-EY-A215	Endometrial	Non-Hispanic	White	0.338	0.662
TCGA-EY-A2OM	Endometrial	Non-Hispanic	White	0.345	0.655

TCGA-EY-A2OP	Endometrial	Non-Hispanic	White	0.893	0.107
TCGA-EY-A2OQ	Endometrial	Non-Hispanic	White	0.983	0.017
TCGA-EY-A548	Endometrial	Non-Hispanic	Black	1.000	0.000
TCGA-EY-A549	Endometrial	Non-Hispanic	Black	1.000	0.000
TCGA-EY-A54A	Endometrial	Non-Hispanic	Black	1.000	0.000
TCGA-EY-A5W2	Endometrial	Non-Hispanic	Black	0.668	0.332
TCGA-EY-A72D	Endometrial	Non-Hispanic	Black	0.856	0.144
TCGA-FI-A2CX	Endometrial	Non-Hispanic	White	0.324	0.676
TCGA-FI-A2D0	Endometrial	Non-Hispanic	White	0.330	0.670
TCGA-FI-A2D4	Endometrial	Unknown	White	0.102	0.898
TCGA-FI-A2D5	Endometrial	Non-Hispanic	White	0.363	0.637
TCGA-FI-A2D6	Endometrial	Non-Hispanic	White	0.379	0.621
TCGA-FI-A2F4	Endometrial	Non-Hispanic	Black	0.266	0.734
TCGA-FI-A2F9	Endometrial	Non-Hispanic	White	0.187	0.813
TCGA-H5-A2HR	Endometrial	Non-Hispanic	Black	0.031	0.969
TCGA-PG-A916	Endometrial	Non-Hispanic	Black	1.000	0.000
TCGA-PG-A917	Endometrial	Non-Hispanic	Black	0.781	0.219
TCGA-QF-A5YT	Endometrial	Non-Hispanic	Black	0.638	0.362
TCGA-QS-A5YQ	Endometrial	Non-Hispanic	Black	0.576	0.424
TCGA-QS-A744	Endometrial	Non-Hispanic	Black	1.000	0.000
TCGA-SJ-A6ZI	Endometrial	Non-Hispanic	Black	1.000	0.000
TCGA-SJ-A6ZJ	Endometrial	Non-Hispanic	Black	0.880	0.120
TCGA-SL-A6J9	Endometrial	Non-Hispanic	Black	0.735	0.265
TCGA-SL-A6JA	Endometrial	Non-Hispanic	Black	0.939	0.061

Table 5. Q-matrix with 2 cluster solution at the subpopulations structure analysis of ovarian cancer TCGA patients. For each patient there is also information about tumor type and self-declared race and ethnicity.

ID	Tumor type	Ethnicity	Race	Cluster 1	Cluster 2
TCGA-04-1331	Ovary	Non-Hispanic	White	0.742	0.258
TCGA-04-1332	Ovary	Non-Hispanic	White	0.843	0.157
TCGA-04-1338	Ovary	Non-Hispanic	White	1.000	0.000
TCGA-04-1341	Ovary	Non-Hispanic	White	1.000	0.000
TCGA-04-1343	Ovary	Non-Hispanic	White	0.832	0.168
TCGA-04-1347	Ovary	Non-Hispanic	White	1.000	0.000
TCGA-04-1350	Ovary	Unknown	White	1.000	0.000
TCGA-04-1356	Ovary	Hispanic	White	0.671	0.329
TCGA-04-1357	Ovary	Unknown	Unknown	0.945	0.055
TCGA-04-1361	Ovary	Unknown	White	0.777	0.223
TCGA-04-1362	Ovary	Unknown	White	0.301	0.699
TCGA-04-1364	Ovary	Non-Hispanic	White	0.899	0.101
TCGA-04-1365	Ovary	Non-Hispanic	White	0.733	0.267

TCGA-04-1514	Ovary	Non-Hispanic	White	0.329	0.671
TCGA-04-1519	Ovary	Unknown	Unknown	0.640	0.360
TCGA-04-1530	Ovary	Non-Hispanic	White	0.643	0.357
TCGA-04-1536	Ovary	Unknown	Black	0.903	0.097
TCGA-04-1648	Ovary	Unknown	White	0.439	0.561
TCGA-04-1651	Ovary	Non-Hispanic	White	0.627	0.373
TCGA-04-1655	Ovary	Unknown	White	0.808	0.192
TCGA-09-0364	Ovary	Non-Hispanic	White	0.565	0.435
TCGA-09-0366	Ovary	Unknown	White	0.230	0.770
TCGA-09-0367	Ovary	Non-Hispanic	Pacific	1.000	0.000
TCGA-09-0369	Ovary	Non-Hispanic	White	1.000	0.000
TCGA-09-1659	Ovary	Non-Hispanic	White	0.851	0.149
TCGA-09-1661	Ovary	Non-Hispanic	White	1.000	0.000
TCGA-09-1662	Ovary	Non-Hispanic	White	0.274	0.726
TCGA-09-1665	Ovary	Non-Hispanic	White	0.522	0.478
TCGA-09-1666	Ovary	Non-Hispanic	White	0.735	0.265
TCGA-09-1667	Ovary	Non-Hispanic	White	0.943	0.057
TCGA-09-1668	Ovary	Non-Hispanic	White	0.723	0.277
TCGA-09-1669	Ovary	Non-Hispanic	White	0.542	0.458
TCGA-09-1670	Ovary	Unknown	White	0.953	0.047
TCGA-09-1673	Ovary	Non-Hispanic	White	0.723	0.277
TCGA-09-2045	Ovary	Non-Hispanic	Asian	0.777	0.223
TCGA-09-2048	Ovary	Non-Hispanic	White	0.669	0.331
TCGA-09-2053	Ovary	Non-Hispanic	White	0.808	0.192
TCGA-09-2054	Ovary	Non-Hispanic	Black	1.000	0.000
TCGA-09-2056	Ovary	Hispanic	White	0.911	0.089
TCGA-10-0927	Ovary	Hispanic	Unknown	1.000	0.000
TCGA-10-0928	Ovary	Unknown	White	0.846	0.154
TCGA-10-0931	Ovary	Unknown	White	0.777	0.223
TCGA-10-0933	Ovary	Non-Hispanic	White	1.000	0.000
TCGA-10-0936	Ovary	Non-Hispanic	Black	0.802	0.198
TCGA-10-0937	Ovary	Unknown	White	0.990	0.010
TCGA-10-0938	Ovary	Unknown	White	0.719	0.281
TCGA-13-0714	Ovary	Non-Hispanic	White	1.000	0.000
TCGA-13-0720	Ovary	Non-Hispanic	White	0.877	0.123
TCGA-13-0724	Ovary	Hispanic	White	1.000	0.000
TCGA-13-0725	Ovary	Hispanic	White	0.932	0.068
TCGA-13-0726	Ovary	Non-Hispanic	White	0.889	0.111
TCGA-13-0727	Ovary	Non-Hispanic	White	0.930	0.070
TCGA-13-0730	Ovary	Non-Hispanic	White	1.000	0.000
TCGA-13-0762	Ovary	Non-Hispanic	White	0.583	0.417
TCGA-13-0765	Ovary	Non-Hispanic	White	0.876	0.124

TCGA-13-0766	Ovary	Non-Hispanic	White	1.000	0.000
TCGA-13-0768	Ovary	Non-Hispanic	White	0.717	0.283
TCGA-13-0795	Ovary	Non-Hispanic	White	1.000	0.000
TCGA-13-0797	Ovary	Non-Hispanic	White	1.000	0.000
TCGA-13-0800	Ovary	Non-Hispanic	White	0.850	0.150
TCGA-13-0804	Ovary	Non-Hispanic	White	1.000	0.000
TCGA-13-0883	Ovary	Non-Hispanic	White	0.644	0.356
TCGA-13-0884	Ovary	Non-Hispanic	White	0.532	0.468
TCGA-13-0885	Ovary	Non-Hispanic	White	1.000	0.000
TCGA-13-0886	Ovary	Non-Hispanic	White	0.678	0.322
TCGA-13-0887	Ovary	Non-Hispanic	White	1.000	0.000
TCGA-13-0888	Ovary	Non-Hispanic	White	1.000	0.000
TCGA-13-0893	Ovary	Non-Hispanic	Black	0.974	0.026
TCGA-13-0897	Ovary	Non-Hispanic	White	1.000	0.000
TCGA-13-0900	Ovary	Non-Hispanic	White	0.944	0.056
TCGA-13-0901	Ovary	Non-Hispanic	Asian	1.000	0.000
TCGA-13-0905	Ovary	Non-Hispanic	White	0.267	0.733
TCGA-13-0906	Ovary	Non-Hispanic	White	0.836	0.164
TCGA-13-0908	Ovary	Non-Hispanic	White	0.120	0.880
TCGA-13-0911	Ovary	Non-Hispanic	White	1.000	0.000
TCGA-13-0913	Ovary	Non-Hispanic	White	1.000	0.000
TCGA-13-0916	Ovary	Non-Hispanic	White	0.656	0.344
TCGA-13-0920	Ovary	Non-Hispanic	White	0.925	0.075
TCGA-13-0923	Ovary	Non-Hispanic	White	1.000	0.000
TCGA-13-0924	Ovary	Non-Hispanic	White	0.954	0.046
TCGA-13-1403	Ovary	Non-Hispanic	White	0.771	0.229
TCGA-13-1404	Ovary	Non-Hispanic	White	0.675	0.325
TCGA-13-1405	Ovary	Non-Hispanic	White	0.868	0.132
TCGA-13-1407	Ovary	Non-Hispanic	White	0.682	0.318
TCGA-13-1408	Ovary	Non-Hispanic	White	0.000	1.000
TCGA-13-1409	Ovary	Non-Hispanic	White	0.440	0.560
TCGA-13-1410	Ovary	Non-Hispanic	White	0.941	0.059
TCGA-13-1411	Ovary	Non-Hispanic	White	0.444	0.556
TCGA-13-1477	Ovary	Non-Hispanic	White	0.571	0.429
TCGA-13-1483	Ovary	Non-Hispanic	White	0.843	0.157
TCGA-13-1485	Ovary	Non-Hispanic	White	0.601	0.399
TCGA-13-1487	Ovary	Non-Hispanic	White	1.000	0.000
TCGA-13-1488	Ovary	Non-Hispanic	White	0.931	0.069
TCGA-13-1489	Ovary	Non-Hispanic	White	0.722	0.278
TCGA-13-1492	Ovary	Non-Hispanic	White	0.782	0.218
TCGA-13-1495	Ovary	Non-Hispanic	White	0.644	0.356
TCGA-13-1496	Ovary	Non-Hispanic	White	0.931	0.069

TCGA-13-1497	Ovary	Non-Hispanic	White	1.000	0.000
TCGA-13-1498	Ovary	Non-Hispanic	White	0.433	0.567
TCGA-13-1499	Ovary	Non-Hispanic	White	0.614	0.386
TCGA-13-1501	Ovary	Non-Hispanic	White	1.000	0.000
TCGA-13-1505	Ovary	Non-Hispanic	White	0.819	0.181
TCGA-13-1506	Ovary	Non-Hispanic	White	0.794	0.206
TCGA-13-1509	Ovary	Non-Hispanic	White	0.627	0.373
TCGA-13-1510	Ovary	Non-Hispanic	White	0.784	0.216
TCGA-13-1511	Ovary	Non-Hispanic	Asian	1.000	0.000
TCGA-13-1512	Ovary	Non-Hispanic	White	1.000	0.000
TCGA-13-2060	Ovary	Unknown	White	0.920	0.080
TCGA-13-A5FT	Ovary	Unknown	Black	0.743	0.257
TCGA-20-0987	Ovary	Non-Hispanic	White	1.000	0.000
TCGA-20-0991	Ovary	Non-Hispanic	White	1.000	0.000
TCGA-20-1682	Ovary	Non-Hispanic	White	1.000	0.000
TCGA-20-1683	Ovary	Non-Hispanic	White	0.612	0.388
TCGA-20-1686	Ovary	Non-Hispanic	White	1.000	0.000
TCGA-20-1687	Ovary	Non-Hispanic	White	1.000	0.000
TCGA-23-1021	Ovary	Non-Hispanic	White	1.000	0.000
TCGA-23-1022	Ovary	Non-Hispanic	White	1.000	0.000
TCGA-23-1023	Ovary	Non-Hispanic	White	0.878	0.122
TCGA-23-1024	Ovary	Non-Hispanic	White	0.533	0.467
TCGA-23-1026	Ovary	Non-Hispanic	White	0.831	0.169
TCGA-23-1027	Ovary	Non-Hispanic	White	0.724	0.276
TCGA-23-1028	Ovary	Hispanic	White	0.997	0.003
TCGA-23-1029	Ovary	Non-Hispanic	White	0.858	0.142
TCGA-23-1030	Ovary	Non-Hispanic	White	0.978	0.022
TCGA-23-1107	Ovary	Non-Hispanic	White	1.000	0.000
TCGA-23-1109	Ovary	Non-Hispanic	White	0.652	0.348
TCGA-23-1110	Ovary	Hispanic	White	1.000	0.000
TCGA-23-1111	Ovary	Unknown	White	0.715	0.285
TCGA-23-1113	Ovary	Non-Hispanic	White	1.000	0.000
TCGA-23-1114	Ovary	Non-Hispanic	White	0.154	0.846
TCGA-23-1116	Ovary	Non-Hispanic	White	1.000	0.000
TCGA-23-1118	Ovary	Non-Hispanic	White	0.465	0.535
TCGA-23-1119	Ovary	Non-Hispanic	White	1.000	0.000
TCGA-23-1120	Ovary	Non-Hispanic	White	0.386	0.614
TCGA-23-1123	Ovary	Non-Hispanic	White	0.885	0.115
TCGA-23-1809	Ovary	Non-Hispanic	White	0.459	0.541
TCGA-23-2077	Ovary	Non-Hispanic	White	0.893	0.107
TCGA-23-2078	Ovary	Non-Hispanic	White	0.459	0.541
TCGA-23-2084	Ovary	Non-Hispanic	White	0.840	0.160

TCGA-24-0966	Ovary	Non-Hispanic	Black	1.000	0.000
TCGA-24-0968	Ovary	Unknown	White	1.000	0.000
TCGA-24-0970	Ovary	Unknown	White	1.000	0.000
TCGA-24-0979	Ovary	Unknown	White	0.970	0.030
TCGA-24-0982	Ovary	Unknown	White	0.768	0.232
TCGA-24-1103	Ovary	Unknown	Black	1.000	0.000
TCGA-24-1104	Ovary	Unknown	White	0.759	0.241
TCGA-24-1105	Ovary	Unknown	White	0.624	0.376
TCGA-24-1413	Ovary	Unknown	White	0.651	0.349
TCGA-24-1416	Ovary	Unknown	White	1.000	0.000
TCGA-24-1417	Ovary	Unknown	White	0.831	0.169
TCGA-24-1418	Ovary	Unknown	White	0.557	0.443
TCGA-24-1419	Ovary	Unknown	White	0.942	0.058
TCGA-24-1422	Ovary	Unknown	Black	0.588	0.412
TCGA-24-1423	Ovary	Unknown	White	0.790	0.210
TCGA-24-1425	Ovary	Unknown	White	0.524	0.476
TCGA-24-1426	Ovary	Unknown	White	0.000	1.000
TCGA-24-1427	Ovary	Unknown	Unknown	0.323	0.677
TCGA-24-1428	Ovary	Unknown	White	0.396	0.604
TCGA-24-1430	Ovary	Unknown	White	0.032	0.968
TCGA-24-1431	Ovary	Unknown	White	1.000	0.000
TCGA-24-1434	Ovary	Unknown	White	0.839	0.161
TCGA-24-1464	Ovary	Unknown	White	1.000	0.000
TCGA-24-1467	Ovary	Unknown	White	0.870	0.130
TCGA-24-1469	Ovary	Unknown	White	0.380	0.620
TCGA-24-1470	Ovary	Unknown	White	0.000	1.000
TCGA-24-1471	Ovary	Unknown	White	1.000	0.000
TCGA-24-1474	Ovary	Unknown	Black	0.782	0.218
TCGA-24-1544	Ovary	Unknown	Black	1.000	0.000
TCGA-24-1546	Ovary	Unknown	White	0.627	0.373
TCGA-24-1550	Ovary	Unknown	White	0.785	0.215
TCGA-24-1551	Ovary	Unknown	White	0.833	0.167
TCGA-24-1552	Ovary	Unknown	White	0.984	0.016
TCGA-24-1553	Ovary	Unknown	White	0.755	0.245
TCGA-24-1557	Ovary	Unknown	White	1.000	0.000
TCGA-24-1560	Ovary	Unknown	White	0.571	0.429
TCGA-24-1562	Ovary	Unknown	White	1.000	0.000
TCGA-24-1563	Ovary	Unknown	Black	1.000	0.000
TCGA-24-1565	Ovary	Unknown	White	0.364	0.636
TCGA-24-1567	Ovary	Unknown	White	0.954	0.046
TCGA-24-1603	Ovary	Unknown	White	0.756	0.244
TCGA-24-1604	Ovary	Unknown	White	0.280	0.720

TCGA-24-1616	Ovary	Unknown	White	1.000	0.000
TCGA-24-1842	Ovary	Unknown	White	0.735	0.265
TCGA-24-1843	Ovary	Unknown	White	0.726	0.274
TCGA-24-1844	Ovary	Unknown	White	0.700	0.300
TCGA-24-1846	Ovary	Unknown	Unknown	0.684	0.316
TCGA-24-1847	Ovary	Unknown	White	0.854	0.146
TCGA-24-1850	Ovary	Unknown	White	1.000	0.000
TCGA-24-1923	Ovary	Unknown	White	0.733	0.267
TCGA-24-1924	Ovary	Unknown	White	0.928	0.072
TCGA-24-1928	Ovary	Unknown	White	0.718	0.282
TCGA-24-1930	Ovary	Unknown	White	0.971	0.029
TCGA-24-2020	Ovary	Unknown	White	0.879	0.121
TCGA-24-2023	Ovary	Unknown	White	0.862	0.138
TCGA-24-2024	Ovary	Unknown	Black	0.028	0.972
TCGA-24-2026	Ovary	Unknown	White	0.634	0.366
TCGA-24-2027	Ovary	Unknown	White	0.289	0.711
TCGA-24-2033	Ovary	Unknown	White	0.868	0.132
TCGA-24-2035	Ovary	Unknown	White	0.944	0.056
TCGA-24-2036	Ovary	Unknown	White	0.000	1.000
TCGA-24-2038	Ovary	Unknown	White	0.528	0.472
TCGA-24-2254	Ovary	Unknown	White	0.722	0.278
TCGA-24-2267	Ovary	Unknown	White	0.815	0.185
TCGA-24-2271	Ovary	Unknown	Asian	0.985	0.015
TCGA-24-2280	Ovary	Unknown	White	0.491	0.509
TCGA-24-2288	Ovary	Unknown	White	0.926	0.074
TCGA-24-2289	Ovary	Unknown	White	0.633	0.367
TCGA-24-2290	Ovary	Unknown	White	0.877	0.123
TCGA-24-2293	Ovary	Unknown	White	0.325	0.675
TCGA-24-2297	Ovary	Unknown	White	0.348	0.652
TCGA-24-2298	Ovary	Unknown	White	0.382	0.618
TCGA-25-1312	Ovary	Non-Hispanic	White	0.896	0.104
TCGA-25-1313	Ovary	Non-Hispanic	White	0.901	0.099
TCGA-25-1315	Ovary	Non-Hispanic	White	0.262	0.738
TCGA-25-1316	Ovary	Non-Hispanic	White	0.772	0.228
TCGA-25-1318	Ovary	Non-Hispanic	White	0.884	0.116
TCGA-25-1319	Ovary	Non-Hispanic	White	1.000	0.000
TCGA-25-1320	Ovary	Non-Hispanic	White	0.262	0.738
TCGA-25-1321	Ovary	Non-Hispanic	White	0.444	0.556
TCGA-25-1322	Ovary	Non-Hispanic	White	0.948	0.052
TCGA-25-1323	Ovary	Non-Hispanic	White	0.257	0.743
TCGA-25-1326	Ovary	Non-Hispanic	White	0.647	0.353
TCGA-25-1328	Ovary	Non-Hispanic	White	0.479	0.521

TCGA-25-1329	Ovary	Non-Hispanic	White	0.285	0.715
TCGA-25-1623	Ovary	Non-Hispanic	White	0.847	0.153
TCGA-25-1626	Ovary	Non-Hispanic	White	0.353	0.647
TCGA-25-1627	Ovary	Non-Hispanic	White	1.000	0.000
TCGA-25-1628	Ovary	Non-Hispanic	White	0.311	0.689
TCGA-25-1630	Ovary	Non-Hispanic	White	0.965	0.035
TCGA-25-1631	Ovary	Non-Hispanic	White	0.697	0.303
TCGA-25-1633	Ovary	Non-Hispanic	White	0.863	0.137
TCGA-25-1634	Ovary	Non-Hispanic	White	1.000	0.000
TCGA-25-1635	Ovary	Non-Hispanic	White	0.635	0.365
TCGA-25-1870	Ovary	Non-Hispanic	White	0.429	0.571
TCGA-25-1877	Ovary	Non-Hispanic	White	0.884	0.116
TCGA-25-2042	Ovary	Non-Hispanic	American Indian	0.921	0.079
TCGA-25-2391	Ovary	Non-Hispanic	White	0.745	0.255
TCGA-25-2392	Ovary	Non-Hispanic	White	0.956	0.044
TCGA-25-2393	Ovary	Non-Hispanic	White	1.000	0.000
TCGA-25-2396	Ovary	Non-Hispanic	White	0.949	0.051
TCGA-25-2398	Ovary	Non-Hispanic	White	0.824	0.176
TCGA-25-2399	Ovary	Non-Hispanic	White	0.550	0.451
TCGA-25-2400	Ovary	Non-Hispanic	White	1.000	0.000
TCGA-25-2401	Ovary	Non-Hispanic	White	1.000	0.000
TCGA-25-2404	Ovary	Non-Hispanic	American Indian	0.816	0.184
TCGA-29-1688	Ovary	Unknown	White	0.690	0.310
TCGA-29-1690	Ovary	Unknown	White	0.871	0.129
TCGA-29-1691	Ovary	Unknown	White	0.891	0.109
TCGA-29-1693	Ovary	Unknown	White	0.907	0.093
TCGA-29-1694	Ovary	Unknown	White	0.405	0.595
TCGA-29-1695	Ovary	Unknown	White	0.833	0.167
TCGA-29-1696	Ovary	Unknown	White	0.937	0.063
TCGA-29-1697	Ovary	Unknown	White	0.711	0.289
TCGA-29-1701	Ovary	Unknown	White	1.000	0.000
TCGA-29-1703	Ovary	Unknown	Black	1.000	0.000
TCGA-29-1705	Ovary	Unknown	White	0.911	0.089
TCGA-29-1707	Ovary	Unknown	White	0.848	0.152
TCGA-29-1710	Ovary	Unknown	White	0.850	0.150
TCGA-29-1711	Ovary	Unknown	Black	1.000	0.000
TCGA-29-1761	Ovary	Unknown	Asian	1.000	0.000
TCGA-29-1762	Ovary	Unknown	White	0.720	0.280
TCGA-29-1763	Ovary	Unknown	Black	0.377	0.623
TCGA-29-1766	Ovary	Unknown	White	1.000	0.000

TCGA-29-1768	Ovary	Unknown	White	0.952	0.048
TCGA-29-1769	Ovary	Unknown	White	1.000	0.000
TCGA-29-1770	Ovary	Unknown	White	1.000	0.000
TCGA-29-1774	Ovary	Non-Hispanic	White	1.000	0.000
TCGA-29-1776	Ovary	Unknown	Black	1.000	0.000
TCGA-29-1777	Ovary	Unknown	White	1.000	0.000
TCGA-29-1778	Ovary	Unknown	White	0.699	0.301
TCGA-29-1781	Ovary	Unknown	White	0.985	0.015
TCGA-29-1783	Ovary	Unknown	White	0.957	0.043
TCGA-29-1784	Ovary	Unknown	White	1.000	0.000
TCGA-29-1785	Ovary	Unknown	White	1.000	0.000
TCGA-29-2414	Ovary	Unknown	White	0.420	0.580
TCGA-29-2425	Ovary	Unknown	White	0.984	0.016
TCGA-29-2428	Ovary	Unknown	White	1.000	0.000
TCGA-29-A5NZ	Ovary	Unknown	Black	0.276	0.724
TCGA-30-1714	Ovary	Non-Hispanic	White	1.000	0.000
TCGA-30-1718	Ovary	Non-Hispanic	White	1.000	0.000
TCGA-30-1853	Ovary	Non-Hispanic	White	0.956	0.044
TCGA-30-1857	Ovary	Non-Hispanic	White	0.965	0.035
TCGA-30-1860	Ovary	Hispanic	White	0.953	0.047
TCGA-30-1861	Ovary	Non-Hispanic	White	0.790	0.210
TCGA-30-1862	Ovary	Non-Hispanic	White	0.574	0.426
TCGA-30-1866	Ovary	Non-Hispanic	White	0.842	0.158
TCGA-30-1891	Ovary	Non-Hispanic	White	1.000	0.000
TCGA-30-1892	Ovary	Non-Hispanic	White	1.000	0.000
TCGA-31-1944	Ovary	Non-Hispanic	White	1.000	0.000
TCGA-31-1946	Ovary	Unknown	Unknown	1.000	0.000
TCGA-31-1950	Ovary	Non-Hispanic	White	0.777	0.223
TCGA-31-1951	Ovary	Non-Hispanic	White	0.591	0.409
TCGA-31-1953	Ovary	Non-Hispanic	Asian	0.809	0.191
TCGA-31-1956	Ovary	Non-Hispanic	White	1.000	0.000
TCGA-31-1959	Ovary	Non-Hispanic	White	1.000	0.000
TCGA-36-1568	Ovary	Unknown	Unknown	0.803	0.197
TCGA-36-1569	Ovary	Unknown	White	0.848	0.152
TCGA-36-1570	Ovary	Unknown	White	0.451	0.549
TCGA-36-1571	Ovary	Unknown	White	0.872	0.128
TCGA-36-1574	Ovary	Unknown	Asian	0.540	0.460
TCGA-36-1576	Ovary	Unknown	Unknown	1.000	0.000
TCGA-36-1577	Ovary	Unknown	Asian	0.529	0.471
TCGA-36-1580	Ovary	Unknown	Unknown	0.812	0.188
TCGA-36-1581	Ovary	Unknown	White	0.679	0.321
TCGA-57-1582	Ovary	Unknown	White	0.834	0.166

TCGA-57-1583	Ovary	Unknown	Black	0.921	0.079
TCGA-57-1584	Ovary	Non-Hispanic	White	0.924	0.076
TCGA-57-1585	Ovary	Non-Hispanic	White	0.674	0.326
TCGA-57-1586	Ovary	Non-Hispanic	White	0.964	0.036
TCGA-57-1994	Ovary	Unknown	White	0.579	0.421
TCGA-59-2350	Ovary	Unknown	Unknown	0.378	0.622
TCGA-59-2351	Ovary	Non-Hispanic	White	0.780	0.220
TCGA-59-2354	Ovary	Non-Hispanic	White	0.887	0.113
TCGA-59-2355	Ovary	Non-Hispanic	White	0.862	0.138
TCGA-59-2363	Ovary	Non-Hispanic	Asian	0.733	0.267
TCGA-59-A5PD	Ovary	Non-Hispanic	Black	0.834	0.166
TCGA-5X-AA5U	Ovary	Unknown	Black	0.596	0.404
TCGA-61-1721	Ovary	Non-Hispanic	White	1.000	0.000
TCGA-61-1724	Ovary	Non-Hispanic	White	0.731	0.269
TCGA-61-1725	Ovary	Non-Hispanic	White	0.960	0.040
TCGA-61-1728	Ovary	Non-Hispanic	White	0.951	0.049
TCGA-61-1736	Ovary	Non-Hispanic	White	0.776	0.224
TCGA-61-1737	Ovary	Non-Hispanic	White	0.726	0.274
TCGA-61-1738	Ovary	Unknown	Black	1.000	0.000
TCGA-61-1741	Ovary	Non-Hispanic	White	0.365	0.635
TCGA-61-1900	Ovary	Non-Hispanic	Black	0.559	0.441
TCGA-61-1907	Ovary	Non-Hispanic	White	0.898	0.102
TCGA-61-1910	Ovary	Non-Hispanic	White	0.874	0.126
TCGA-61-1911	Ovary	Non-Hispanic	White	1.000	0.000
TCGA-61-1914	Ovary	Unknown	Unknown	1.000	0.000
TCGA-61-1918	Ovary	Non-Hispanic	White	0.918	0.082
TCGA-61-1919	Ovary	Non-Hispanic	White	1.000	0.000
TCGA-61-1995	Ovary	Non-Hispanic	White	0.763	0.237
TCGA-61-1998	Ovary	Non-Hispanic	White	0.942	0.058
TCGA-61-2000	Ovary	Non-Hispanic	White	1.000	0.000
TCGA-61-2002	Ovary	Non-Hispanic	White	0.964	0.036
TCGA-61-2003	Ovary	Non-Hispanic	White	0.747	0.253
TCGA-61-2008	Ovary	Non-Hispanic	Asian	0.577	0.423
TCGA-61-2009	Ovary	Non-Hispanic	White	1.000	0.000
TCGA-61-2012	Ovary	Non-Hispanic	White	0.830	0.170
TCGA-61-2088	Ovary	Non-Hispanic	White	1.000	0.000
TCGA-61-2092	Ovary	Unknown	White	1.000	0.000
TCGA-61-2097	Ovary	Unknown	White	0.783	0.217
TCGA-61-2098	Ovary	Unknown	White	0.373	0.627
TCGA-61-2101	Ovary	Non-Hispanic	White	0.821	0.179
TCGA-61-2102	Ovary	Unknown	White	1.000	0.000
TCGA-61-2104	Ovary	Unknown	White	0.794	0.206

TCGA-61-2113	Ovary	Non-Hispanic	White	0.718	0.282
TCGA-OY-A56Q	Ovary	Non-Hispanic	Black	0.786	0.214
TCGA-VG-A8LO	Ovary	Non-Hispanic	Black	0.563	0.437
TCGA-WR-A838	Ovary	Non-Hispanic	Black	0.303	0.697

Table 6. Q-matrix with 4 cluster solution at the subpopulations structure analysis of ovarian cancer TCGA patients. For each patient there is also information about tumor type and self-declared race and ethnicity.

ID	Tumor type	Ethnicity	Race	Cluster 1	Cluster 2	Cluster 3	Cluster 4
TCGA-04-1331	Ovary	Non-Hispanic	White	0.176	0.643	0.000	0.181
TCGA-04-1332	Ovary	Non-Hispanic	White	0.185	0.743	0.012	0.060
TCGA-04-1338	Ovary	Non-Hispanic	White	0.032	0.954	0.014	0.000
TCGA-04-1341	Ovary	Non-Hispanic	White	0.123	0.877	0.000	0.000
TCGA-04-1343	Ovary	Non-Hispanic	White	0.349	0.612	0.013	0.027
TCGA-04-1347	Ovary	Non-Hispanic	White	0.111	0.833	0.056	0.000
TCGA-04-1350	Ovary	Unknown	White	0.000	0.892	0.108	0.000
TCGA-04-1356	Ovary	Hispanic	White	0.000	0.641	0.000	0.359
TCGA-04-1357	Ovary	Unknown	Unknown	0.033	0.878	0.056	0.033
TCGA-04-1361	Ovary	Unknown	White	0.217	0.615	0.021	0.147
TCGA-04-1362	Ovary	Unknown	White	0.000	0.068	0.617	0.315
TCGA-04-1364	Ovary	Non-Hispanic	White	0.305	0.695	0.000	0.000
TCGA-04-1365	Ovary	Non-Hispanic	White	0.293	0.496	0.211	0.000
TCGA-04-1514	Ovary	Non-Hispanic	White	0.000	0.323	0.000	0.677
TCGA-04-1519	Ovary	Unknown	Unknown	0.000	0.617	0.000	0.383
TCGA-04-1530	Ovary	Non-Hispanic	White	0.000	0.438	0.517	0.045
TCGA-04-1536	Ovary	Unknown	Black	0.000	0.796	0.204	0.000
TCGA-04-1648	Ovary	Unknown	White	0.000	0.340	0.233	0.427
TCGA-04-1651	Ovary	Non-Hispanic	White	0.000	0.594	0.000	0.406
TCGA-04-1655	Ovary	Unknown	White	0.000	0.597	0.403	0.000
TCGA-09-0364	Ovary	Non-Hispanic	White	0.045	0.434	0.287	0.235
TCGA-09-0366	Ovary	Unknown	White	0.623	0.000	0.000	0.377
TCGA-09-0367	Ovary	Non-Hispanic	Pacific	0.000	1.000	0.000	0.000
TCGA-09-0369	Ovary	Non-Hispanic	White	0.074	0.880	0.046	0.000
TCGA-09-1659	Ovary	Non-Hispanic	White	0.272	0.728	0.000	0.000
TCGA-09-1661	Ovary	Non-Hispanic	White	0.000	0.918	0.082	0.000
TCGA-09-1662	Ovary	Non-Hispanic	White	0.000	0.268	0.000	0.732
TCGA-09-1665	Ovary	Non-Hispanic	White	0.927	0.073	0.000	0.000
TCGA-09-1666	Ovary	Non-Hispanic	White	0.000	0.698	0.000	0.302
TCGA-09-1667	Ovary	Non-Hispanic	White	0.000	0.864	0.017	0.119
TCGA-09-1668	Ovary	Non-Hispanic	White	0.000	0.706	0.000	0.294
TCGA-09-1669	Ovary	Non-Hispanic	White	0.086	0.310	0.559	0.045
TCGA-09-1670	Ovary	Unknown	White	0.019	0.850	0.130	0.000
TCGA-09-1673	Ovary	Non-Hispanic	White	0.000	0.704	0.000	0.296
TCGA-09-2045	Ovary	Non-Hispanic	Asian	0.000	0.745	0.000	0.255
TCGA-09-2048	Ovary	Non-Hispanic	White	0.614	0.386	0.000	0.000
TCGA-09-2053	Ovary	Non-Hispanic	White	0.064	0.731	0.000	0.205
TCGA-09-2054	Ovary	Non-Hispanic	Black	0.119	0.881	0.000	0.000

TCGA-09-2056	Ovary	Hispanic	White	0.000	0.767	0.233	0.000
TCGA-10-0927	Ovary	Hispanic	Unknown	0.000	1.000	0.000	0.000
TCGA-10-0928	Ovary	Unknown	White	0.000	0.818	0.000	0.182
TCGA-10-0931	Ovary	Unknown	White	0.031	0.716	0.000	0.254
TCGA-10-0933	Ovary	Non-Hispanic	White	0.164	0.836	0.000	0.000
TCGA-10-0936	Ovary	Non-Hispanic	Black	0.387	0.613	0.000	0.000
TCGA-10-0937	Ovary	Unknown	White	0.000	0.914	0.086	0.000
TCGA-10-0938	Ovary	Unknown	White	0.000	0.567	0.300	0.133
TCGA-13-0714	Ovary	Non-Hispanic	White	0.000	1.000	0.000	0.000
TCGA-13-0720	Ovary	Non-Hispanic	White	0.000	0.843	0.000	0.157
TCGA-13-0724	Ovary	Hispanic	White	0.233	0.767	0.000	0.000
TCGA-13-0725	Ovary	Hispanic	White	0.000	0.861	0.052	0.088
TCGA-13-0726	Ovary	Non-Hispanic	White	0.000	0.751	0.249	0.000
TCGA-13-0727	Ovary	Non-Hispanic	White	0.000	0.862	0.114	0.024
TCGA-13-0730	Ovary	Non-Hispanic	White	0.000	1.000	0.000	0.000
TCGA-13-0762	Ovary	Non-Hispanic	White	0.000	0.570	0.000	0.430
TCGA-13-0765	Ovary	Non-Hispanic	White	0.162	0.703	0.123	0.012
TCGA-13-0766	Ovary	Non-Hispanic	White	0.000	1.000	0.000	0.000
TCGA-13-0768	Ovary	Non-Hispanic	White	0.094	0.409	0.497	0.000
TCGA-13-0795	Ovary	Non-Hispanic	White	0.105	0.895	0.000	0.000
TCGA-13-0797	Ovary	Non-Hispanic	White	0.000	1.000	0.000	0.000
TCGA-13-0800	Ovary	Non-Hispanic	White	0.356	0.644	0.000	0.000
TCGA-13-0804	Ovary	Non-Hispanic	White	0.069	0.913	0.000	0.019
TCGA-13-0883	Ovary	Non-Hispanic	White	0.000	0.455	0.457	0.088
TCGA-13-0884	Ovary	Non-Hispanic	White	0.000	0.334	0.519	0.147
TCGA-13-0885	Ovary	Non-Hispanic	White	0.000	0.945	0.055	0.000
TCGA-13-0886	Ovary	Non-Hispanic	White	0.000	0.415	0.585	0.000
TCGA-13-0887	Ovary	Non-Hispanic	White	0.000	1.000	0.000	0.000
TCGA-13-0888	Ovary	Non-Hispanic	White	0.092	0.908	0.000	0.000
TCGA-13-0893	Ovary	Non-Hispanic	Black	0.000	0.872	0.128	0.000
TCGA-13-0897	Ovary	Non-Hispanic	White	0.000	0.978	0.000	0.022
TCGA-13-0900	Ovary	Non-Hispanic	White	0.099	0.852	0.000	0.049
TCGA-13-0901	Ovary	Non-Hispanic	Asian	0.147	0.837	0.000	0.016
TCGA-13-0905	Ovary	Non-Hispanic	White	0.000	0.158	0.320	0.522
TCGA-13-0906	Ovary	Non-Hispanic	White	0.000	0.812	0.000	0.188
TCGA-13-0908	Ovary	Non-Hispanic	White	0.000	0.000	0.684	0.316
TCGA-13-0911	Ovary	Non-Hispanic	White	0.000	0.987	0.000	0.013
TCGA-13-0913	Ovary	Non-Hispanic	White	0.000	1.000	0.000	0.000
TCGA-13-0916	Ovary	Non-Hispanic	White	0.020	0.606	0.000	0.374
TCGA-13-0920	Ovary	Non-Hispanic	White	0.171	0.695	0.135	0.000
TCGA-13-0923	Ovary	Non-Hispanic	White	0.000	1.000	0.000	0.000
TCGA-13-0924	Ovary	Non-Hispanic	White	0.000	0.925	0.000	0.075

TCGA-13-1403	Ovary	Non-Hispanic	White	0.007	0.734	0.000	0.258
TCGA-13-1404	Ovary	Non-Hispanic	White	0.026	0.549	0.290	0.135
TCGA-13-1405	Ovary	Non-Hispanic	White	0.000	0.791	0.034	0.175
TCGA-13-1407	Ovary	Non-Hispanic	White	0.825	0.175	0.000	0.000
TCGA-13-1408	Ovary	Non-Hispanic	White	0.543	0.000	0.372	0.085
TCGA-13-1409	Ovary	Non-Hispanic	White	0.000	0.145	0.795	0.060
TCGA-13-1410	Ovary	Non-Hispanic	White	0.081	0.784	0.136	0.000
TCGA-13-1411	Ovary	Non-Hispanic	White	0.000	0.361	0.159	0.481
TCGA-13-1477	Ovary	Non-Hispanic	White	0.000	0.273	0.727	0.000
TCGA-13-1483	Ovary	Non-Hispanic	White	0.005	0.814	0.000	0.181
TCGA-13-1485	Ovary	Non-Hispanic	White	0.000	0.576	0.000	0.424
TCGA-13-1487	Ovary	Non-Hispanic	White	0.292	0.708	0.000	0.000
TCGA-13-1488	Ovary	Non-Hispanic	White	0.000	0.868	0.034	0.098
TCGA-13-1489	Ovary	Non-Hispanic	White	0.155	0.632	0.000	0.212
TCGA-13-1492	Ovary	Non-Hispanic	White	0.000	0.749	0.000	0.251
TCGA-13-1495	Ovary	Non-Hispanic	White	0.000	0.444	0.499	0.058
TCGA-13-1496	Ovary	Non-Hispanic	White	0.156	0.745	0.054	0.046
TCGA-13-1497	Ovary	Non-Hispanic	White	0.000	1.000	0.000	0.000
TCGA-13-1498	Ovary	Non-Hispanic	White	0.295	0.201	0.289	0.215
TCGA-13-1499	Ovary	Non-Hispanic	White	0.280	0.416	0.107	0.196
TCGA-13-1501	Ovary	Non-Hispanic	White	0.167	0.833	0.000	0.000
TCGA-13-1505	Ovary	Non-Hispanic	White	0.000	0.789	0.000	0.211
TCGA-13-1506	Ovary	Non-Hispanic	White	0.210	0.648	0.000	0.142
TCGA-13-1509	Ovary	Non-Hispanic	White	0.000	0.421	0.493	0.086
TCGA-13-1510	Ovary	Non-Hispanic	White	0.000	0.764	0.000	0.236
TCGA-13-1511	Ovary	Non-Hispanic	Asian	0.000	0.982	0.018	0.000
TCGA-13-1512	Ovary	Non-Hispanic	White	0.021	0.937	0.041	0.000
TCGA-13-2060	Ovary	Unknown	White	0.230	0.676	0.094	0.000
TCGA-13-A5FT	Ovary	Unknown	Black	0.448	0.481	0.070	0.000
TCGA-20-0987	Ovary	Non-Hispanic	White	0.000	1.000	0.000	0.000
TCGA-20-0991	Ovary	Non-Hispanic	White	0.043	0.957	0.000	0.000
TCGA-20-1682	Ovary	Non-Hispanic	White	0.000	0.988	0.000	0.012
TCGA-20-1683	Ovary	Non-Hispanic	White	0.000	0.314	0.686	0.000
TCGA-20-1686	Ovary	Non-Hispanic	White	0.031	0.955	0.014	0.000
TCGA-20-1687	Ovary	Non-Hispanic	White	0.000	0.989	0.011	0.000
TCGA-23-1021	Ovary	Non-Hispanic	White	0.000	1.000	0.000	0.000
TCGA-23-1022	Ovary	Non-Hispanic	White	0.000	1.000	0.000	0.000
TCGA-23-1023	Ovary	Non-Hispanic	White	0.069	0.769	0.162	0.000
TCGA-23-1024	Ovary	Non-Hispanic	White	0.335	0.071	0.594	0.000
TCGA-23-1026	Ovary	Non-Hispanic	White	0.000	0.731	0.181	0.088
TCGA-23-1027	Ovary	Non-Hispanic	White	0.000	0.600	0.230	0.170
TCGA-23-1028	Ovary	Hispanic	White	0.000	0.955	0.020	0.025

TCGA-23-1029	Ovary	Non-Hispanic	White	0.000	0.818	0.000	0.182
TCGA-23-1030	Ovary	Non-Hispanic	White	0.000	0.880	0.007	0.113
TCGA-23-1107	Ovary	Non-Hispanic	White	0.000	1.000	0.000	0.000
TCGA-23-1109	Ovary	Non-Hispanic	White	0.032	0.311	0.657	0.000
TCGA-23-1110	Ovary	Hispanic	White	0.050	0.950	0.000	0.000
TCGA-23-1111	Ovary	Unknown	White	0.000	0.681	0.000	0.319
TCGA-23-1113	Ovary	Non-Hispanic	White	0.000	0.986	0.014	0.000
TCGA-23-1114	Ovary	Non-Hispanic	White	1.000	0.000	0.000	0.000
TCGA-23-1116	Ovary	Non-Hispanic	White	0.000	1.000	0.000	0.000
TCGA-23-1118	Ovary	Non-Hispanic	White	0.266	0.332	0.000	0.402
TCGA-23-1119	Ovary	Non-Hispanic	White	0.138	0.862	0.000	0.000
TCGA-23-1120	Ovary	Non-Hispanic	White	0.175	0.322	0.000	0.503
TCGA-23-1123	Ovary	Non-Hispanic	White	0.000	0.847	0.000	0.153
TCGA-23-1809	Ovary	Non-Hispanic	White	0.546	0.252	0.035	0.166
TCGA-23-2077	Ovary	Non-Hispanic	White	0.347	0.653	0.000	0.000
TCGA-23-2078	Ovary	Non-Hispanic	White	0.000	0.339	0.286	0.375
TCGA-23-2084	Ovary	Non-Hispanic	White	0.016	0.712	0.272	0.000
TCGA-24-0966	Ovary	Non-Hispanic	Black	0.005	0.995	0.000	0.000
TCGA-24-0968	Ovary	Unknown	White	0.247	0.753	0.000	0.000
TCGA-24-0970	Ovary	Unknown	White	0.128	0.872	0.000	0.000
TCGA-24-0979	Ovary	Unknown	White	0.182	0.795	0.000	0.023
TCGA-24-0982	Ovary	Unknown	White	0.000	0.576	0.424	0.000
TCGA-24-1103	Ovary	Unknown	Black	0.085	0.915	0.000	0.000
TCGA-24-1104	Ovary	Unknown	White	0.238	0.646	0.000	0.116
TCGA-24-1105	Ovary	Unknown	White	0.699	0.301	0.000	0.000
TCGA-24-1413	Ovary	Unknown	White	0.140	0.388	0.472	0.000
TCGA-24-1416	Ovary	Unknown	White	0.155	0.845	0.000	0.000
TCGA-24-1417	Ovary	Unknown	White	0.000	0.797	0.011	0.192
TCGA-24-1418	Ovary	Unknown	White	0.000	0.466	0.174	0.360
TCGA-24-1419	Ovary	Unknown	White	0.062	0.824	0.114	0.000
TCGA-24-1422	Ovary	Unknown	Black	0.109	0.526	0.000	0.365
TCGA-24-1423	Ovary	Unknown	White	0.336	0.664	0.000	0.000
TCGA-24-1425	Ovary	Unknown	White	0.000	0.498	0.000	0.502
TCGA-24-1426	Ovary	Unknown	White	0.000	0.000	0.629	0.371
TCGA-24-1427	Ovary	Unknown	Unknown	0.000	0.028	0.798	0.174
TCGA-24-1428	Ovary	Unknown	White	0.042	0.373	0.055	0.529
TCGA-24-1430	Ovary	Unknown	White	0.000	0.000	0.490	0.510
TCGA-24-1431	Ovary	Unknown	White	0.000	1.000	0.000	0.000
TCGA-24-1434	Ovary	Unknown	White	0.371	0.629	0.000	0.000
TCGA-24-1464	Ovary	Unknown	White	0.000	1.000	0.000	0.000
TCGA-24-1467	Ovary	Unknown	White	0.000	0.806	0.000	0.194
TCGA-24-1469	Ovary	Unknown	White	0.436	0.000	0.564	0.000

TCGA-24-1470	Ovary	Unknown	White	0.077	0.000	0.364	0.559
TCGA-24-1471	Ovary	Unknown	White	0.000	0.982	0.000	0.018
TCGA-24-1474	Ovary	Unknown	Black	0.133	0.696	0.000	0.170
TCGA-24-1544	Ovary	Unknown	Black	0.000	0.987	0.013	0.000
TCGA-24-1546	Ovary	Unknown	White	0.000	0.554	0.201	0.245
TCGA-24-1550	Ovary	Unknown	White	0.519	0.481	0.000	0.000
TCGA-24-1551	Ovary	Unknown	White	0.000	0.740	0.191	0.069
TCGA-24-1552	Ovary	Unknown	White	0.000	0.939	0.061	0.000
TCGA-24-1553	Ovary	Unknown	White	0.179	0.630	0.000	0.192
TCGA-24-1557	Ovary	Unknown	White	0.000	0.972	0.000	0.028
TCGA-24-1560	Ovary	Unknown	White	0.000	0.552	0.000	0.448
TCGA-24-1562	Ovary	Unknown	White	0.000	1.000	0.000	0.000
TCGA-24-1563	Ovary	Unknown	Black	0.142	0.857	0.000	0.000
TCGA-24-1565	Ovary	Unknown	White	0.422	0.011	0.558	0.008
TCGA-24-1567	Ovary	Unknown	White	0.000	0.893	0.000	0.107
TCGA-24-1603	Ovary	Unknown	White	0.000	0.740	0.000	0.260
TCGA-24-1604	Ovary	Unknown	White	0.000	0.292	0.000	0.708
TCGA-24-1616	Ovary	Unknown	White	0.000	1.000	0.000	0.000
TCGA-24-1842	Ovary	Unknown	White	0.000	0.687	0.000	0.313
TCGA-24-1843	Ovary	Unknown	White	0.667	0.333	0.000	0.000
TCGA-24-1844	Ovary	Unknown	White	0.453	0.473	0.000	0.074
TCGA-24-1846	Ovary	Unknown	Unknown	0.000	0.667	0.000	0.333
TCGA-24-1847	Ovary	Unknown	White	0.000	0.813	0.000	0.187
TCGA-24-1850	Ovary	Unknown	White	0.113	0.887	0.000	0.000
TCGA-24-1923	Ovary	Unknown	White	0.130	0.660	0.000	0.210
TCGA-24-1924	Ovary	Unknown	White	0.000	0.884	0.072	0.044
TCGA-24-1928	Ovary	Unknown	White	0.000	0.499	0.501	0.000
TCGA-24-1930	Ovary	Unknown	White	0.157	0.843	0.000	0.000
TCGA-24-2020	Ovary	Unknown	White	0.214	0.786	0.000	0.000
TCGA-24-2023	Ovary	Unknown	White	0.057	0.804	0.000	0.139
TCGA-24-2024	Ovary	Unknown	Black	0.361	0.000	0.000	0.639
TCGA-24-2026	Ovary	Unknown	White	0.757	0.243	0.000	0.000
TCGA-24-2027	Ovary	Unknown	White	0.000	0.106	0.552	0.342
TCGA-24-2033	Ovary	Unknown	White	0.000	0.833	0.040	0.127
TCGA-24-2035	Ovary	Unknown	White	0.104	0.896	0.000	0.000
TCGA-24-2036	Ovary	Unknown	White	0.000	0.000	0.294	0.706
TCGA-24-2038	Ovary	Unknown	White	0.000	0.466	0.085	0.449
TCGA-24-2254	Ovary	Unknown	White	0.000	0.689	0.000	0.311
TCGA-24-2267	Ovary	Unknown	White	0.000	0.794	0.000	0.206
TCGA-24-2271	Ovary	Unknown	Asian	0.000	0.978	0.006	0.016
TCGA-24-2280	Ovary	Unknown	White	0.412	0.336	0.000	0.253
TCGA-24-2288	Ovary	Unknown	White	0.146	0.837	0.000	0.017

TCGA-24-2289	Ovary	Unknown	White	0.314	0.489	0.000	0.198
TCGA-24-2290	Ovary	Unknown	White	0.000	0.831	0.000	0.169
TCGA-24-2293	Ovary	Unknown	White	0.655	0.092	0.000	0.253
TCGA-24-2297	Ovary	Unknown	White	0.138	0.292	0.000	0.569
TCGA-24-2298	Ovary	Unknown	White	0.000	0.065	0.935	0.000
TCGA-25-1312	Ovary	Non-Hispanic	White	0.091	0.800	0.000	0.109
TCGA-25-1313	Ovary	Non-Hispanic	White	0.000	0.828	0.142	0.029
TCGA-25-1315	Ovary	Non-Hispanic	White	0.237	0.000	0.588	0.174
TCGA-25-1316	Ovary	Non-Hispanic	White	0.000	0.681	0.170	0.149
TCGA-25-1318	Ovary	Non-Hispanic	White	0.000	0.816	0.122	0.062
TCGA-25-1319	Ovary	Non-Hispanic	White	0.000	1.000	0.000	0.000
TCGA-25-1320	Ovary	Non-Hispanic	White	0.261	0.171	0.000	0.569
TCGA-25-1321	Ovary	Non-Hispanic	White	0.231	0.204	0.411	0.153
TCGA-25-1322	Ovary	Non-Hispanic	White	0.000	0.859	0.030	0.112
TCGA-25-1323	Ovary	Non-Hispanic	White	0.649	0.000	0.000	0.351
TCGA-25-1326	Ovary	Non-Hispanic	White	0.042	0.383	0.575	0.000
TCGA-25-1328	Ovary	Non-Hispanic	White	0.000	0.462	0.000	0.538
TCGA-25-1329	Ovary	Non-Hispanic	White	0.221	0.097	0.341	0.340
TCGA-25-1623	Ovary	Non-Hispanic	White	0.000	0.666	0.334	0.000
TCGA-25-1626	Ovary	Non-Hispanic	White	0.252	0.228	0.000	0.521
TCGA-25-1627	Ovary	Non-Hispanic	White	0.000	1.000	0.000	0.000
TCGA-25-1628	Ovary	Non-Hispanic	White	0.000	0.000	1.000	0.000
TCGA-25-1630	Ovary	Non-Hispanic	White	0.000	0.914	0.000	0.086
TCGA-25-1631	Ovary	Non-Hispanic	White	0.038	0.478	0.484	0.000
TCGA-25-1633	Ovary	Non-Hispanic	White	0.097	0.772	0.000	0.132
TCGA-25-1634	Ovary	Non-Hispanic	White	0.000	0.944	0.000	0.056
TCGA-25-1635	Ovary	Non-Hispanic	White	0.152	0.429	0.273	0.146
TCGA-25-1870	Ovary	Non-Hispanic	White	0.000	0.449	0.000	0.551
TCGA-25-1877	Ovary	Non-Hispanic	White	0.241	0.651	0.099	0.009
TCGA-25-2042	Ovary	Non-Hispanic	American Indian	0.000	0.893	0.000	0.107
TCGA-25-2391	Ovary	Non-Hispanic	White	0.094	0.680	0.023	0.203
TCGA-25-2392	Ovary	Non-Hispanic	White	0.000	0.920	0.000	0.080
TCGA-25-2393	Ovary	Non-Hispanic	White	0.220	0.758	0.021	0.000
TCGA-25-2396	Ovary	Non-Hispanic	White	0.000	0.925	0.000	0.075
TCGA-25-2398	Ovary	Non-Hispanic	White	0.000	0.658	0.342	0.000
TCGA-25-2399	Ovary	Non-Hispanic	White	0.206	0.164	0.631	0.000
TCGA-25-2400	Ovary	Non-Hispanic	White	0.000	0.986	0.000	0.014
TCGA-25-2401	Ovary	Non-Hispanic	White	0.000	1.000	0.000	0.000
TCGA-25-2404	Ovary	Non-Hispanic	American Indian	0.000	0.778	0.000	0.222
TCGA-29-1688	Ovary	Unknown	White	0.000	0.444	0.556	0.000
TCGA-29-1690	Ovary	Unknown	White	0.000	0.829	0.000	0.171
TCGA-29-1691	Ovary	Unknown	White	0.123	0.811	0.014	0.053

TCGA-29-1693	Ovary	Unknown	White	0.000	0.865	0.000	0.134
TCGA-29-1694	Ovary	Unknown	White	0.000	0.395	0.000	0.605
TCGA-29-1695	Ovary	Unknown	White	0.000	0.720	0.280	0.000
TCGA-29-1696	Ovary	Unknown	White	0.000	0.894	0.000	0.106
TCGA-29-1697	Ovary	Unknown	White	0.000	0.674	0.000	0.326
TCGA-29-1701	Ovary	Unknown	White	0.055	0.893	0.053	0.000
TCGA-29-1703	Ovary	Unknown	Black	0.000	1.000	0.000	0.000
TCGA-29-1705	Ovary	Unknown	White	0.000	0.872	0.000	0.127
TCGA-29-1707	Ovary	Unknown	White	0.000	0.814	0.000	0.186
TCGA-29-1710	Ovary	Unknown	White	0.280	0.622	0.099	0.000
TCGA-29-1711	Ovary	Unknown	Black	0.000	0.921	0.000	0.079
TCGA-29-1761	Ovary	Unknown	Asian	0.026	0.974	0.000	0.000
TCGA-29-1762	Ovary	Unknown	White	0.000	0.629	0.196	0.175
TCGA-29-1763	Ovary	Unknown	Black	0.205	0.016	0.734	0.045
TCGA-29-1766	Ovary	Unknown	White	0.137	0.863	0.000	0.000
TCGA-29-1768	Ovary	Unknown	White	0.000	0.921	0.000	0.079
TCGA-29-1769	Ovary	Unknown	White	0.000	1.000	0.000	0.000
TCGA-29-1770	Ovary	Unknown	White	0.000	1.000	0.000	0.000
TCGA-29-1774	Ovary	Non-Hispanic	White	0.055	0.851	0.094	0.000
TCGA-29-1776	Ovary	Unknown	Black	0.000	1.000	0.000	0.000
TCGA-29-1777	Ovary	Unknown	White	0.000	0.981	0.019	0.000
TCGA-29-1778	Ovary	Unknown	White	0.000	0.493	0.507	0.000
TCGA-29-1781	Ovary	Unknown	White	0.000	0.902	0.098	0.000
TCGA-29-1783	Ovary	Unknown	White	0.078	0.797	0.125	0.000
TCGA-29-1784	Ovary	Unknown	White	0.113	0.887	0.000	0.000
TCGA-29-1785	Ovary	Unknown	White	0.031	0.963	0.000	0.006
TCGA-29-2414	Ovary	Unknown	White	0.649	0.000	0.351	0.000
TCGA-29-2425	Ovary	Unknown	White	0.000	0.943	0.000	0.057
TCGA-29-2428	Ovary	Unknown	White	0.131	0.869	0.000	0.000
TCGA-29-A5NZ	Ovary	Unknown	Black	0.309	0.171	0.000	0.520
TCGA-30-1714	Ovary	Non-Hispanic	White	0.117	0.883	0.000	0.000
TCGA-30-1718	Ovary	Non-Hispanic	White	0.018	0.947	0.035	0.000
TCGA-30-1853	Ovary	Non-Hispanic	White	0.000	0.938	0.000	0.062
TCGA-30-1857	Ovary	Non-Hispanic	White	0.000	0.870	0.130	0.000
TCGA-30-1860	Ovary	Hispanic	White	0.000	0.852	0.148	0.000
TCGA-30-1861	Ovary	Non-Hispanic	White	0.274	0.609	0.116	0.000
TCGA-30-1862	Ovary	Non-Hispanic	White	0.000	0.267	0.733	0.000
TCGA-30-1866	Ovary	Non-Hispanic	White	0.308	0.482	0.210	0.000
TCGA-30-1891	Ovary	Non-Hispanic	White	0.000	0.929	0.071	0.000
TCGA-30-1892	Ovary	Non-Hispanic	White	0.000	1.000	0.000	0.000
TCGA-31-1944	Ovary	Non-Hispanic	White	0.000	1.000	0.000	0.000
TCGA-31-1946	Ovary	Unknown	Unknown	0.104	0.896	0.000	0.000

TCGA-31-1950	Ovary	Non-Hispanic	White	0.444	0.556	0.000	0.000
TCGA-31-1951	Ovary	Non-Hispanic	White	0.000	0.331	0.669	0.000
TCGA-31-1953	Ovary	Non-Hispanic	Asian	0.000	0.762	0.000	0.238
TCGA-31-1956	Ovary	Non-Hispanic	White	0.000	0.966	0.000	0.034
TCGA-31-1959	Ovary	Non-Hispanic	White	0.000	0.988	0.000	0.012
TCGA-36-1568	Ovary	Unknown	Unknown	0.156	0.751	0.000	0.093
TCGA-36-1569	Ovary	Unknown	White	0.096	0.760	0.000	0.144
TCGA-36-1570	Ovary	Unknown	White	0.126	0.253	0.366	0.255
TCGA-36-1571	Ovary	Unknown	White	0.000	0.830	0.000	0.170
TCGA-36-1574	Ovary	Unknown	Asian	0.000	0.405	0.336	0.259
TCGA-36-1576	Ovary	Unknown	Unknown	0.000	0.970	0.000	0.030
TCGA-36-1577	Ovary	Unknown	Asian	0.000	0.190	0.810	0.000
TCGA-36-1580	Ovary	Unknown	Unknown	0.000	0.793	0.000	0.207
TCGA-36-1581	Ovary	Unknown	White	0.520	0.405	0.000	0.075
TCGA-57-1582	Ovary	Unknown	White	0.000	0.688	0.312	0.000
TCGA-57-1583	Ovary	Unknown	Black	0.000	0.908	0.000	0.092
TCGA-57-1584	Ovary	Non-Hispanic	White	0.000	0.859	0.057	0.084
TCGA-57-1585	Ovary	Non-Hispanic	White	0.129	0.587	0.056	0.228
TCGA-57-1586	Ovary	Non-Hispanic	White	0.234	0.747	0.019	0.000
TCGA-57-1994	Ovary	Unknown	White	0.395	0.428	0.000	0.176
TCGA-59-2350	Ovary	Unknown	Unknown	0.000	0.233	0.406	0.361
TCGA-59-2351	Ovary	Non-Hispanic	White	0.241	0.559	0.200	0.000
TCGA-59-2354	Ovary	Non-Hispanic	White	0.000	0.835	0.000	0.165
TCGA-59-2355	Ovary	Non-Hispanic	White	0.062	0.791	0.000	0.146
TCGA-59-2363	Ovary	Non-Hispanic	Asian	0.000	0.699	0.020	0.281
TCGA-59-A5PD	Ovary	Non-Hispanic	Black	0.000	0.777	0.016	0.208
TCGA-5X-AA5U	Ovary	Unknown	Black	0.685	0.315	0.000	0.000
TCGA-61-1721	Ovary	Non-Hispanic	White	0.000	1.000	0.000	0.000
TCGA-61-1724	Ovary	Non-Hispanic	White	0.082	0.544	0.375	0.000
TCGA-61-1725	Ovary	Non-Hispanic	White	0.000	0.934	0.000	0.066
TCGA-61-1728	Ovary	Non-Hispanic	White	0.153	0.808	0.039	0.000
TCGA-61-1736	Ovary	Non-Hispanic	White	0.000	0.564	0.436	0.000
TCGA-61-1737	Ovary	Non-Hispanic	White	0.000	0.495	0.505	0.000
TCGA-61-1738	Ovary	Unknown	Black	0.200	0.800	0.000	0.000
TCGA-61-1741	Ovary	Non-Hispanic	White	0.000	0.124	0.604	0.273
TCGA-61-1900	Ovary	Non-Hispanic	Black	0.260	0.303	0.310	0.127
TCGA-61-1907	Ovary	Non-Hispanic	White	0.000	0.784	0.216	0.000
TCGA-61-1910	Ovary	Non-Hispanic	White	0.000	0.837	0.000	0.163
TCGA-61-1911	Ovary	Non-Hispanic	White	0.000	1.000	0.000	0.000
TCGA-61-1914	Ovary	Unknown	Unknown	0.000	1.000	0.000	0.000
TCGA-61-1918	Ovary	Non-Hispanic	White	0.000	0.910	0.000	0.090
TCGA-61-1919	Ovary	Non-Hispanic	White	0.051	0.949	0.000	0.000

TCGA-61-1995	Ovary	Non-Hispanic	White	0.000	0.714	0.000	0.286
TCGA-61-1998	Ovary	Non-Hispanic	White	0.000	0.875	0.125	0.000
TCGA-61-2000	Ovary	Non-Hispanic	White	0.015	0.985	0.000	0.000
TCGA-61-2002	Ovary	Non-Hispanic	White	0.000	0.909	0.000	0.091
TCGA-61-2003	Ovary	Non-Hispanic	White	0.000	0.660	0.167	0.173
TCGA-61-2008	Ovary	Non-Hispanic	Asian	0.000	0.408	0.373	0.219
TCGA-61-2009	Ovary	Non-Hispanic	White	0.000	0.894	0.106	0.000
TCGA-61-2012	Ovary	Non-Hispanic	White	0.516	0.484	0.000	0.000
TCGA-61-2088	Ovary	Non-Hispanic	White	0.172	0.828	0.000	0.000
TCGA-61-2092	Ovary	Unknown	White	0.000	0.964	0.000	0.036
TCGA-61-2097	Ovary	Unknown	White	0.000	0.685	0.195	0.119
TCGA-61-2098	Ovary	Unknown	White	0.974	0.026	0.000	0.000
TCGA-61-2101	Ovary	Non-Hispanic	White	0.159	0.709	0.000	0.132
TCGA-61-2102	Ovary	Unknown	White	0.000	1.000	0.000	0.000
TCGA-61-2104	Ovary	Unknown	White	0.386	0.614	0.000	0.000
TCGA-61-2113	Ovary	Non-Hispanic	White	0.383	0.546	0.000	0.071
TCGA-OY-A56Q	Ovary	Non-Hispanic	Black	0.000	0.675	0.164	0.161
TCGA-VG-A8LO	Ovary	Non-Hispanic	Black	0.169	0.490	0.000	0.341
TCGA-WR-A838	Ovary	Non-Hispanic	Black	0.000	0.308	0.035	0.657

As with STRUCTURE and ADMIXTURE, we took VCF files from UIHC and TCGA and merged them for analysis. We then used PLINK software to filter the VCF files by minor allele frequency at q > 0.05 and by linkage disequilibrium (LD) < 0.1 r2 for pairs of markers inside 200kb [1]. The filtering process was performed to identify independent markers within the sequenced samples and resulted in 9,611 markers in both cohorts. Then, we imputed missing genotypes for all samples utilizing BEAGLE 4.1 to obtain genotype coverage of >99% for all markers [2].

Analysis of the data with the *SNPRelate* package showed the presence of some differences in the genetic substructure of both cohorts, UIHC and TCGA, in the principal component analysis (PCA, **Supplementary Figures B1, and B2**).

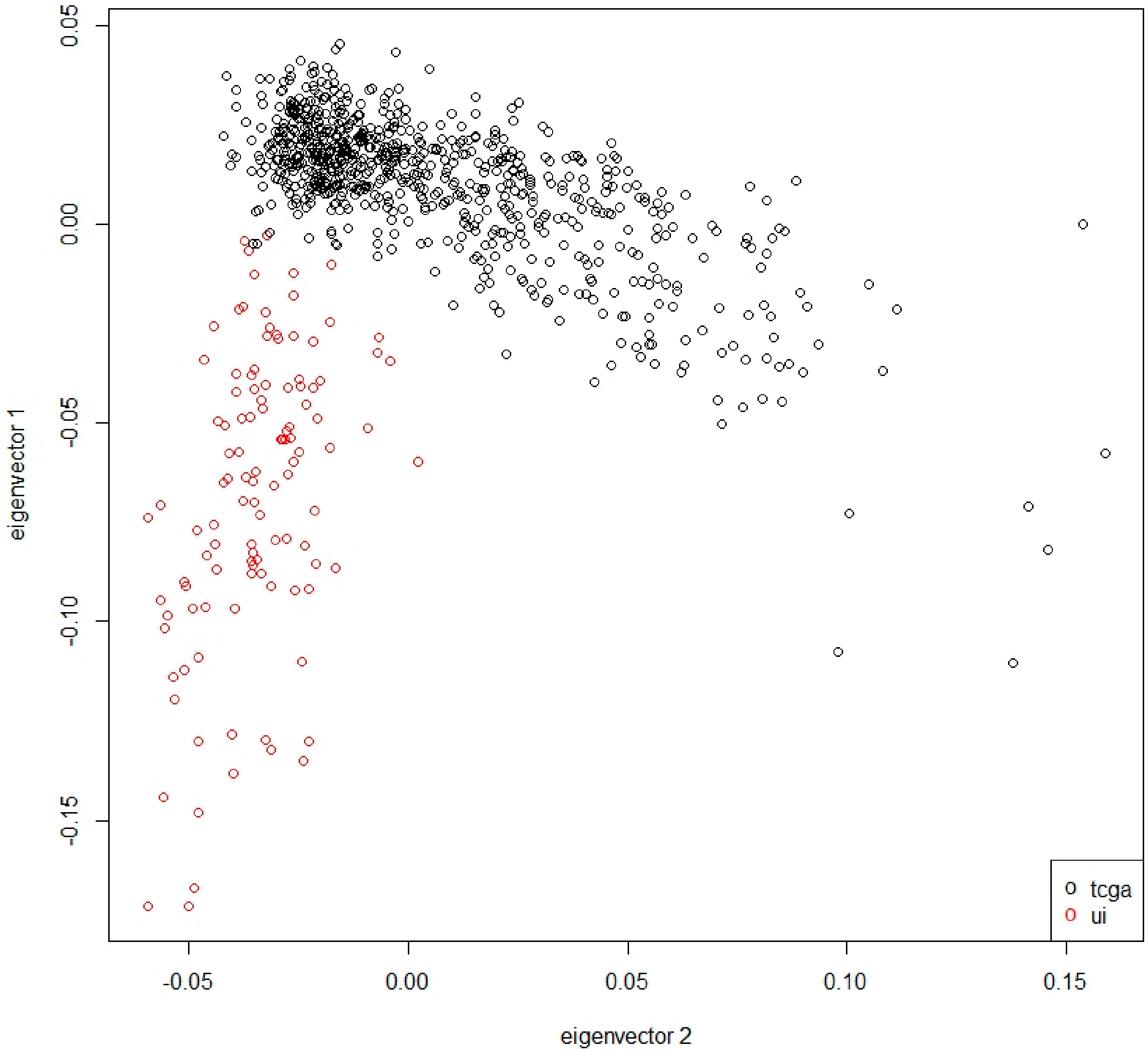

Figure SB1: Representation of the first two eigenvectors of the PCA analysis between UIHC and TCGA cohorts. The first two eigenvectors hold the largest percentage of variance between both cohorts and the total variance accounted for is over two-thirds of the total. ui: UIHC cohort; tcga: TCGA cohort.

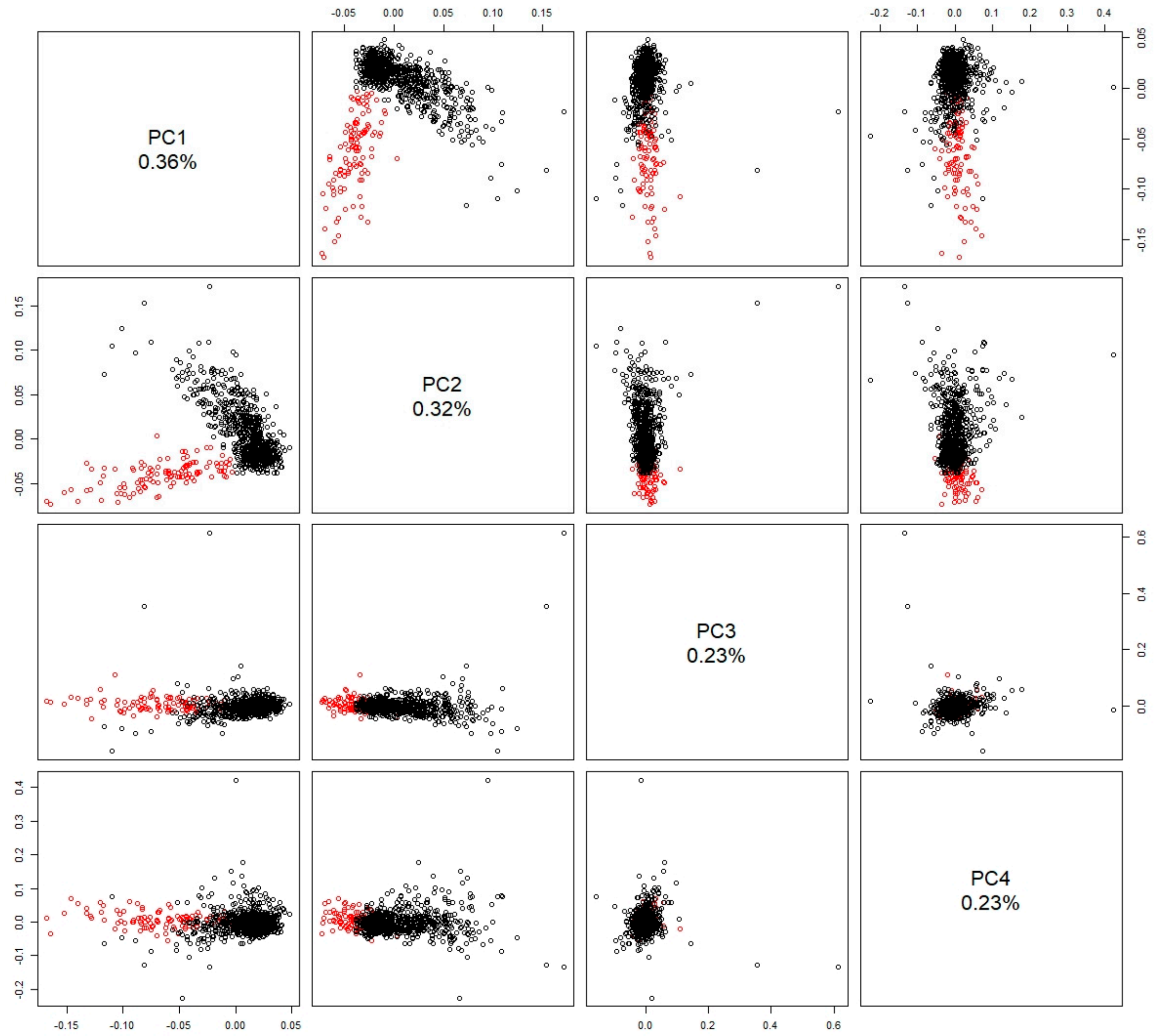

Figure SB2: Representation of the principal component pairs for the first four **principal components.**

The mean F_{ST} estimate between UIHC and TCGA was 0.015. Similar F_{ST} estimates have been observed when comparing populations from the Middle East, Europe, and Central/South Asia. However, F_{ST} estimates also depend on the number of populations compared as well as on their sizes [3], making a finer interpretation difficult.

References

1. Purcell, S.; Neale, B.; Todd-Brown, K.; Thomas, L.; Ferreira, M.A.; Bender, D.; Maller, J.; Sklar, P.; de Bakker, P.I.; Daly, M.J.; et al. PLINK: A tool set for whole-genome association and population-based linkage analyses. *Am. J. Hum. Genet.* **2007**, *81*, 559–575.
2. Browning, B.L.; Browning, S.R. Genotype Imputation with Millions of Reference Samples. *Am. J. Hum. Genet.* **2016**, *98*, 116–126.
3. Alcala, N.; Rosenberg, N.A. Mathematical Constraints on FST: Biallelic Markers in Arbitrarily Many Populations. *Genetics* **2017**, *206*, 1581–1600.
